# Ancient *Yersinia pestis* genomes from across Western Europe reveal early diversification during the First Pandemic (541–750)

**DOI:** 10.1073/pnas.1820447116

**Published:** 2019-06-04

**Authors:** Marcel Keller, Maria A. Spyrou, Christiana L. Scheib, Gunnar U. Neumann, Andreas Kröpelin, Brigitte Haas-Gebhard, Bernd Päffgen, Jochen Haberstroh, Albert Ribera i Lacomba, Claude Raynaud, Craig Cessford, Raphaël Durand, Peter Stadler, Kathrin Nägele, Jessica S. Bates, Bernd Trautmann, Sarah A. Inskip, Joris Peters, John E. Robb, Toomas Kivisild, Dominique Castex, Michael McCormick, Kirsten I. Bos, Michaela Harbeck, Alexander Herbig, Johannes Krause

**Affiliations:** ^a^Department of Archaeogenetics, Max Planck Institute for the Science of Human History, 07745 Jena, Germany;; ^b^State Collection of Anthropology and Palaeoanatomy Munich, Staatliche Naturwissenschaftliche Sammlungen Bayerns, 80333 Munich, Germany;; ^c^Department of Archaeology, University of Cambridge, Cambridge CB2 3ER, United Kingdom;; ^d^Institute of Genomics, University of Tartu, 51010 Tartu, Estonia;; ^e^Friedrich Schiller University Jena, 07743 Jena, Germany;; ^f^Archaeological Collection of the Bavarian State, 80538 Munich, Germany;; ^g^Institute for Pre- and Protohistoric Archaeology and Archaeology of the Roman Provinces, Ludwig Maximilian University Munich, 80799 Munich, Germany;; ^h^Bavarian State Department of Monuments and Sites, 80539 Munich, Germany;; ^i^Department for Municipal Archaeology, Valencia City Council, 46014 Valencia, Spain;; ^j^CNRS, UMR5140, Archéologie des Sociétés Méditerranéennes, 34199 Montpellier, France;; ^k^Service d’Archéologie Préventive de l’Agglomération de Bourges Plus, 18023 Bourges Cedex, France;; ^l^Department of Pre- and Protohistory, University of Vienna, 1190 Vienna, Austria;; ^m^McDonald Institute for Archaeological Research, University of Cambridge, Cambridge CB2 3ER, United Kingdom;; ^n^ArchaeoBioCenter, Ludwig Maximilian University Munich, 80539 Munich, Germany;; ^o^Department of Veterinary Sciences, Institute of Palaeoanatomy, Domestication Research and the History of Veterinary Medicine, Ludwig Maximilian University Munich, 80539 Munich, Germany;; ^p^Department of Human Genetics, Katholieke Universiteit Leuven, 3000 Leuven, Belgium;; ^q^UMR 5199, PACEA, CNRS Institute, 33615 Pessac Cedex, France;; ^r^Initiative for the Science of the Human Past, Department of History, Harvard University, Cambridge, MA 02138;; ^s^Max Planck–Harvard Research Center for the Archaeoscience of the Ancient Mediterranean, 07745 Jena, Germany

**Keywords:** Justinianic Plague, ancient DNA, bacterial evolution, Anglo-Saxons, Merovingians

## Abstract

The first historically documented pandemic caused by *Yersinia pestis* began as the Justinianic Plague in 541 within the Roman Empire and continued as the so-called First Pandemic until 750. Although paleogenomic studies have previously identified the causative agent as *Y. pestis*, little is known about the bacterium’s spread, diversity, and genetic history over the course of the pandemic. To elucidate the microevolution of the bacterium during this time period, we screened human remains from 21 sites in Austria, Britain, Germany, France, and Spain for *Y. pestis* DNA and reconstructed eight genomes. We present a methodological approach assessing single-nucleotide polymorphisms (SNPs) in ancient bacterial genomes, facilitating qualitative analyses of low coverage genomes from a metagenomic background. Phylogenetic analysis on the eight reconstructed genomes reveals the existence of previously undocumented *Y. pestis* diversity during the sixth to eighth centuries, and provides evidence for the presence of multiple distinct *Y. pestis* strains in Europe. We offer genetic evidence for the presence of the Justinianic Plague in the British Isles, previously only hypothesized from ambiguous documentary accounts, as well as the parallel occurrence of multiple derived strains in central and southern France, Spain, and southern Germany. Four of the reported strains form a polytomy similar to others seen across the *Y. pestis* phylogeny, associated with the Second and Third Pandemics. We identified a deletion of a 45-kb genomic region in the most recent First Pandemic strains affecting two virulence factors, intriguingly overlapping with a deletion found in 17th- to 18th-century genomes of the Second Pandemic.

## Significance

The first historically reported pandemic attributed to *Yersinia pestis* started with the Justinianic Plague (541–544) and continued for around 200 y as the so-called First Pandemic. To date, only one *Y. pestis* strain from this pandemic has been reconstructed using ancient DNA. In this study, we present eight genomes from Britain, France, Germany, and Spain, demonstrating the geographic range of plague during the First Pandemic and showing microdiversity in the Early Medieval Period. Moreover, we detect similar genome decay during the First and Second Pandemics (14th to 18th century) that includes the same two virulence factors, thus providing an example of potential convergent evolution of *Y. pestis* during large-scale epidemics.

*Yersinia pestis*, the causative agent of plague, is a Gram-negative bacterium that predominantly infects rodents and is transmitted by their ectoparasites such as fleas. As a zoonosis, it is also able to infect humans with a mortality rate of 50–100% without antibiotic treatment (1), manifesting as bubonic, septicemic, or pneumonic plague. In addition to the ancient foci that exist in Central and East Asia, the pathogen spread worldwide at the end of the 19th century in the so-called Third Pandemic that started in 1855 in Yunnan, China, establishing new local foci in Africa and the Americas. Today, *Y. pestis* causes sporadic infections annually and occasional local recurrent epidemics such as that documented in 2017 in Madagascar (2).

Although recent paleogenetic analyses have reconstructed an ancient form of *Y. pestis* that infected humans as early as in the prehistoric period [2,900–1,700 BCE (3–6)], the First Pandemic (541–750) is the earliest historically recorded pandemic clearly attributed to *Y. pestis* (7, 8), starting with the fulminant Justinianic Plague (541–544). It was later followed by the Second Pandemic, which started with the Black Death of 1346–1353 (9, 10) and persisted in Europe until the 18th century (11–13).

The 2000s saw first attempts to amplify *Y. pestis-*specific DNA fragments from burials of the sixth century (14–16). Although early studies on two French sites (15, 16) are controversial due to methodological limitations (17) and proved inconsistent with a later genotyping study (18), more recent studies have been successful in authenticating the latter and reconstructing whole *Y. pestis* genomes from two early medieval burial sites in modern-day Bavaria, Germany (7, 8).

These genomic investigations identified a previously unknown lineage associated with the First Pandemic that was found to be genetically identical at both sites and falls within the modern diversity of *Y. pestis*. Moreover, this lineage is distinct from those associated with the Second Pandemic that started ∼800 y later, indicating two independent emergence events.

Although these studies have unequivocally demonstrated the involvement of *Y. pestis* in the First Pandemic, the published genomes represent a single outbreak, leaving the genetic diversity of that time entirely unexplored. Here, we assess the diversity and microevolution of *Y. pestis* during that time by analyzing multiple and mass burials from a broader temporal and spatial scope than previously attempted. After screening 183 samples from 21 archaeological sites, we were able to reconstruct eight genomes with higher than 4.5-fold mean coverage from Britain, France, Germany, and Spain. Furthermore, we identified a large deletion in the most recent First Pandemic strains that affects the same region as a deletion observed in late Second Pandemic strains, suggesting similar mechanism of pathogen adaptation in the waning period of the two separate pandemics.

## Results

### Screening and Capture.

We used a previously described qPCR assay (19) that targets the *Y. pestis*-specific *pla* gene on the pPCP1 plasmid to test 171 teeth from a minimum of 122 individuals from 20 sites, spanning from ∼300 to 900 CE (*SI Appendix*, Table S1). For the remaining site, Edix Hill, Britain, the 22 samples only had shotgun sequencing data available, and therefore, pathogen DNA screening was performed using the metagenomic tool MALT (20). This analysis revealed six putatively *Y. pestis*-positive samples after visual inspection of aligned reads in MEGAN (21) (*SI Appendix*, Table S4). All 30 PCR-positive extracts and 5 of the Edix Hill samples were subsequently turned into double-stranded, double-indexed, and UDG-treated DNA libraries and were enriched for the *Y. pestis* genome following an in-solution capture approach (20).

Whereas some samples reached up to 38.1-fold chromosomal mean coverage after whole-genome capture, nine of the PCR-positive samples yielded a coverage of lower than 0.1-fold. Since the qPCR assay can amplify nonspecific products and subsequent capture can enrich for environmental DNA that sporadically maps to the *Y. pestis* reference, it is crucial to differentiate between samples that show low DNA preservation and those that are false positives.

**Fig. 1.**
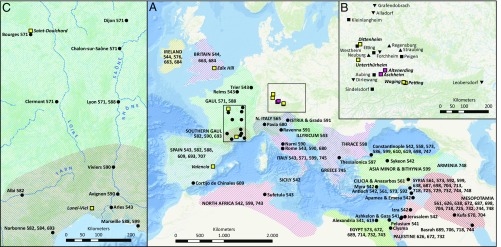
Geographic extent of the First Pandemic and sampled sites. (*A*) Map of historically documented occurrences of plague (regions shaded, cities depicted by circles, both with respective years of occurrence) between 541 and 750 in Europe and the Mediterranean basin. All sources are given in *SI Appendix*. Sites with genomic evidence for *Y. pestis* are shown as pink (previously published) and yellow squares (presented here). (*B*) Enlarged rectangular space of *A* (*Right*) showing all sites in Germany and Austria that were included in this study. Sites tested negative are depicted in black upward-pointing triangles (burials dating before 541), squares (dating around 541–544), and downward-pointing triangles (dating after 544). (*C*) Enlarged *Inset* of *A* (*Left*) shows reported occurrences in France and main rivers.

False-positive samples are unlikely to show similar mapping success on all genetic elements compared with true-positive samples. Therefore, mapping to all three plasmids was used in combination with a statistical outlier detection for the verification of low coverage genomes. Ratios of reads mapping to the *Y. pestis* chromosome and the three individual plasmids were determined to normalize for the variable coverage between samples. Since the samples were captured with the same probe set and assuming no vast differences in plasmid copy number, the ratios should be consistent over all positive samples independent of their genomic coverage. This is, however, not expected for false-positive samples. Therefore, we calculated the Mahalanobis distance (22), a standard method for outlier detection in multivariate datasets, to find false-positive and authenticate low coverage true-positive samples (χ^2^ = 5.991, df = 2, *P* = 0.05; *SI Appendix*, Table S2). Five samples, EDI002.A, DIR002.A, LVC001.B, LVC001.C, and PEI001.A, were classified as outliers. Despite having chromosomal coverage, DIR002.A, EDI002.A, and PEI001.A had no or only a few reads mapping to the plasmids and were therefore considered as *Y. pestis* negative. LVC001.B and LVC001.C had an exceptionally high ratio of reads mapping to pPCP1 and are still considered positive. The remaining 33 samples come from four sites in Germany (Dittenheim [DIT], *n* = 3; Petting [PET], *n* = 3; Waging [WAG], *n* = 1; Unterthürheim [UNT], *n* = 5), two in France (Lunel-Viel [LVC], *n* = 6; Saint-Doulchard [LSD], *n* = 11), one in Britain (Edix Hill [EDI], *n* = 4), and one in Spain (Valencia [VAL], *n* = 1; Table 1 and Fig. 1).

**Table 1.** List of all sites that were tested with country in brackets (AUS = Austria, DEU = Germany, ESP = Spain, FRA = France, GBR = Great Britain)

Site	Lab ID	Context	Graves in total	Multiple burials	Time frame	Positive/total samples
Alladorf (DEU)	ALL	Separate burial area (*Hofgrablege*)	163	5×2	630–720	0/6
Dirlewang (DEU)	DIR	Early medieval cemetery	40	2×2	650–700	0/2
Dittenheim (DEU)	DIT	Early medieval cemetery	238, 10 crem.	4×2	550–700	3/9
Edix Hill (GBR)	EDI	Early medieval cemetery	115	1×4, 9×2	500–650	4/22
Forchheim (DEU)	FOR	Special burial	1	1×4	650–700	0/3
Grafendobrach (DEU)	GRA	Settlement burials (*Hofgrablege*)	85	1×3, 1×2+1	850–930	0/3
Kleinlangheim (DEU)	KLH	Early medieval cemetery	244, 56 crem.	8×2, 1×3	470–720	0/5
Leobersdorf (AUS)	LEO	Early medieval cemetery	154	16×2, 4×3, 2×4, 1×5	640–800	0/3
Lunel-Viel Les Horts (FRA)	LVH	Early medieval cemetery	140	1×2	475–700	0/5
Lunel-Viel Quartier centrale (FRA)	LVC	Demolition trench inhumations	—	6+2 individuals in 2 trenches	400–600	6/16
München-Aubing (DEU)	AUB	Early medieval cemetery	896	4×2	400–700	0/8
Neuburg an der Donau (DEU)	NEU	Late Roman cemetery	130	3×2, 1×3	300–400	0/2
Peigen (DEU)	PEI	Early medieval cemetery	274	3×2	450–700	0/5
Petting (DEU)	PET	Early medieval cemetery	721	min. 1×3, 2×2, 1×2+1	530–730	3/7
Regensburg Fritz-Fend-Str. (DEU)	RFF	Late Roman cemetery	115, 48 crem.	2×2	350–450	0/3
Saint-Doulchard Le Pressoir (FRA)	LSD	Early medieval cemetery	175	12×2, 2×3	530–1200	11/26
Sindelsdorf (DEU)	SIN	Early medieval cemetery	331	3×2, 1×3+1	500–720	0/5
Straubing Azlburg I/II (DEU)	SAZ	Late Roman cemetery	541, 1 crem.	2×2, 1×3	300–450	0/3
Unterthürheim (DEU)	UNT	Early medieval cemetery	256	14×2, 2×3, 1×4	525–680	5/7
Valencia, Plaça de Almoina (ESP)	VAL	Visigothic intramural cemetery	67	3×2, 3×3, 4×4, 2×5, 15×5+	500–700	1/36
Waging (DEU)	WAG	Early medieval cemetery	239	min. 2×2, 1×2+1	530–700	1/12
Westheim (DEU)	WES	Early medieval cemetery	228	5×2, 1×3	500–650	0/3

The number of graves is counting multiple burials as single graves; cremations are counted separately. Multiple burials are listed as number of graves times number of individuals (5x2 translates to five double burials, and 1x2+1 to one double burial associated with a single burial). Detailed site descriptions are given in *SI Appendix*, and a table of all screened samples in *SI Appendix*, Table S1.

After mapping to the chromosome, 10 genomes showed a higher than 4.5-fold mean coverage and were used for downstream analyses. These were DIT003.B (9.4-fold), EDI001.A (38.1-fold), EDI003.A (5.2-fold), EDI004.A (7.5-fold), LSD001.A (4.8-fold), LSD023.A (7.2-fold), PET004.A (5.6-fold), VAL001.B (9.6-fold), as well as UNT003.A and UNT004.A (7.6-fold and 5.2-fold, respectively) (*SI Appendix*, Table S3). The raw reads of six positive samples of the individuals LVC001, LVC005, and LVC006 were combined to yield a single genome with a mean coverage of 6.7-fold for the site of Lunel-Viel after assuring that they represent an identical strain. From each site, only the genome with the highest coverage was used for phylogenetic analyses when multiple genomes were available but shown to be identical in the evaluation of their single-nucleotide polymorphism (SNP) profiles. As such, the genomes of EDI003.A, EDI004.A, and UNT004.A were omitted.

### SNP Evaluation.

Phylogenetic analyses based on SNP alignments are prone to wrong topologies and artificial branches introduced by false-positive SNPs. This is especially true for low-coverage genomes that derive from metagenomic specimens. In the context of ancient pathogen DNA, there are three main sources for false-positive SNPs: First, DNA damage such as deamination of cytosine to uracil can lead to misincorporation of nucleotides during sample processing (23). Second, the mapping of closely related environmental species to the reference sequence of the target organism is likely, especially for conserved regions of the genome (24). Third, mapping of short reads is more prone to mismapping and calling of false-positive SNPs generated at sites of genome rearrangement. Whereas the first source can be circumvented via in vitro protocols like UDG treatment (25), the latter two can be reduced but not eliminated with strict mapping parameters and exclusion of problematic regions (26) as applied here. A fourth source for false SNP assignments could result from multiple genetically distinct strains that would lead to a chimeric sequence. The latter was not observed in our data (*SI Appendix*, Fig. S1) and this phenomenon might be limited to chronic infections with pathogens such as *Mycobacterium tuberculosis*, where mixed infections have been previously documented (27). The introduction of false-positive SNPs by sequencing errors is stochastically negligible as shown in simulated datasets (*SI Appendix*).

The retrieval of genomes that span a wide geographic area gives us the opportunity to assess *Y. pestis* microdiversity present in Europe during the First Pandemic. Given that our genomes are of relatively low genomic coverage, we critically evaluated uniquely called and shared SNPs among the First Pandemic genomes to accurately determine their phylogenetic position. This analysis was performed for all genomes retrieved from UDG-treated libraries with higher than 4.5-fold mean coverage, including the previously published high-quality Altenerding genome (17.2-fold mean coverage).

For this, we developed the tool “SNPEvaluation” and defined three different criteria, all applying for a 50-bp window surrounding the SNP: (A) Comparing the mean coverage after BWA mapping with high and low stringency and excluding all SNPs that showed a higher coverage under low stringent mapping than in high stringent mapping. In metagenomic datasets, reads of related species map frequently to conserved regions in the reference genome. When the position is not covered by reads from the target organism (*Y. pestis*) but the genomic region is similar enough in other environmental organisms so that their reads can map, they might mimic a SNP in *Y. pestis* when the contaminant species carries a different allele in that position. (B) Excluding all SNPs for which heterozygous calls were identified in the surrounding regions. Heterozygous calls accumulate in conserved regions due to the above-described effect. (C) Excluding all SNPs within regions that include positions that lack genomic coverage. Variants in genome architecture often appear as gaps in mapped data and are likely to cause mapping errors, potentially resulting in false-positive SNPs.

The performance of the presented tool and the validity of the selected criteria were assessed using simulated datasets, where each dataset consisted of both reads of a known *Y. pestis* genotype (CO92) with different coverages and reads from a captured sample previously determined as *Y. pestis*-negative (DIR002.A) for representation of an environmental background that is typical for ancient DNA datasets. The SNP evaluation on the artificial datasets—applied with the same criteria as for the First Pandemic genomes presented in this study—showed a maximum sensitivity (all false-positive CO92 SNPs detected) and a high specificity (3.49–8.57% of true-positive CO92 positions erroneously filtered out; *SI Appendix*, Figs. S2 and S3 and Tables S5, S6, and S7).

**Fig. 2.**
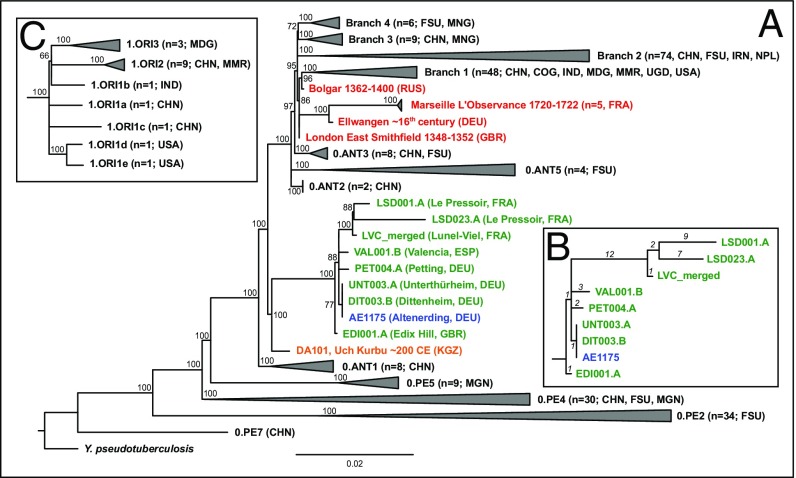
Phylogenetic tree. (*A*) Maximum-likelihood tree with full SNP alignment (6,580 positions) of 233 modern *Y. pestis* and one *Y. pseudotuberculosis* genome, 10 published (second- to third-century Tian Shan in orange; Altenerding in blue; Second Pandemic in red) and eight genomes presented here (green) with country given in brackets (DEU = Germany, ESP = Spain, FRA = France, GBR = Great Britain, RUS = Russia). Numbers and origins of modern genomes are given in brackets (CHN = China, COG = Congo, FSU = Former Soviet Union, IND = India, IRN = Iran, MDG = Madagascar, MMR = Myanmar, MNG = Mongolia, NPL = Nepal, UGA = Uganda). Numbers on nodes are showing bootstrap values (1,000 iterations). (*B*) Detailed, manually drawn tree of the First Pandemic genomes showing all remaining SNP positions after SNP evaluation (number of SNPs given in italics). (*C*) Detailed tree of the 1.ORI clade within branch 1, showing the polytomy.

This evaluation was applied to all nonshared SNPs within the First Pandemic lineage, totaling between 1 and 87 chromosomal SNPs per genome (*SI Appendix*, Table S8). Forty-four chromosomal SNPs, three SNPs on the pCD1 plasmids, and two on the pMT1 plasmid were classified as true positive across all 11 genomes (*SI Appendix*, Table S10). The following 39 chromosomal SNPs appear unambiguous and phylogenetically informative (see “Tree” in *SI Appendix*, Table S10 and Fig. 2*B*): The Edix Hill genomes (EDI001.A, EDI003.A, and EDI004.A) share one unique SNP, and they are missing one SNP previously identified in Altenerding and shared in all other genomes. The Altenerding genome (AE1175) as well as the genomes of Unterthürheim (UNT003.A, UNT004.A) and Dittenheim (DIT003.B) appear identical after SNP evaluation at all positions. The genomes from Petting (PET004.A) and Valencia (VAL001.B) appear distinct, each occupying a unique branch composed of two and three unique SNPs, respectively (Fig. 2*B* and *SI Appendix*, Table S10). The genomes of Lunel-Viel (LVC_merged) and Saint-Doulchard (LSD001.A, LSD023.A) share in total 12 SNPs and Lunel-Viel has one more unique derived SNP. The two genomes of Saint-Doulchard share two additional SNPs but are separated by branches of nine (LSD001.A) and seven SNPs (LSD023.A). Three SNPs, two of which were previously called in Altenerding (*SI Appendix*, Table S5), were identified as potentially shared among all First Pandemic genomes and therefore classified as uninformative for the microdiversity. One SNP might be shared among all genomes except Edix Hill, but cannot be reliably classified due to low coverage in multiple samples. Another SNP appears to be homoplastic since it appears in the Altenerding cluster and Saint-Doulchard, but not in Lunel-Viel. Whereas the low-coverage genomes of LSD007.A, LSD019.A, LSD020.A, LSD021.A, LSD022.A, and LSD024.A appear identical with LSD023.A by full or partial coverage of its unique SNPs, the genomes of LSD002.A, LSD013.A, and LSD026.A cannot be assigned to the genotype of LSD023.A or LSD001.A, since none of their unique SNPs is covered in these genomes.

On the pCD1 plasmid, one SNP was identified as missing in the Edix Hill genomes (EDI001.A, EDI003.A, EDI004.A), one as shared between both Saint-Doulchard genomes (LSD001.A, LSD023.A), and one as unique to the genome LSD001.A. One additional SNP was found on the pMT1 plasmid in the Valencia genome (VAL001.B). An analysis of the Aschheim genome as well as a SNP effect analysis is presented in *SI Appendix*, Tables S7 and S14.

SNPs shared by at least two genomes without a conflicting call in any other genome were evaluated as potentially shared SNPs among the First Pandemic lineage. We applied the exact same parameters as for the nonshared SNPs, but also considered positions with less than threefold coverage (*SI Appendix*, Table S11). Only SNPs that pass all three criteria of our SNP evaluation in at least half of the analyzed genomes (i.e., 6 out of 12) were accepted as true shared SNPs, reducing the number from 50 SNPs identified in a previous study (7)—after removal of nonshared and ambiguous SNPs—to 45.

The Waging sample (WAG001.A) had a genomic coverage too low for inclusion in our phylogenetic analysis. Since it was the only sample giving evidence for *Y. pestis* presence at this site, it was assessed for all SNPs that were either shared or unique in the other First Pandemic genomes. Visual inspection revealed 7 of the 43 shared SNPs to be present in the WAG001.A genome at low coverage (less than threefold) and one SNP absent in Edix Hill but potentially present in all other genomes. For both shared and unique SNPs, no conflicting positions were found. This strain could, therefore, be attributed to the First Pandemic lineage without, however, resolving its exact phylogenetic position (*SI Appendix*, Table S11).

### Phylogenetic Analysis.

A set of 233 modern *Y. pestis* genomes (*SI Appendix*, Table S12) as well as 7 Second Pandemic genomes, including a representative of the Black Death strain (London) and 7 post-Black Death genomes [14th-century Bolgar, 16th-century Ellwangen (12); 18th-century Marseille (13)], and an ancient genome from Tian Shan [DA101, second to third century (28)] were used for phylogenetic analyses alongside our First Pandemic genomes presented here (*SI Appendix*, Table S3) and the previously published genome of Altenerding. The *Y. pseudotuberculosis* isolate IP32953 (29) was used as an outgroup.

Our maximum-likelihood tree (30) constructed from the full SNP alignment reveals that all of the genomes presented here occupy positions on the same lineage (Fig. 2*A* and *SI Appendix*, Fig. S4). This confirms their authenticity and is congruent with previous association of this lineage to the First Pandemic (541–750). In addition, the previously reported genome from Altenerding (2148) is identical to the genomes from Dittenheim (DIT003.B) and Unterthürheim (UNT003.A) presented here. Moreover, the genomes of Petting (PET004.A), Valencia (VAL001.B), and the clade giving rise to the French genomes of Lunel-Viel (LVC_merged) and Saint-Doulchard (LSD001.A, LSD023.A) seem to diverge from the Altenerding cluster through a polytomy (Fig. 2*B*; 88% bootstrap support). The French clade further diversifies into two branches, one giving rise to Lunel-Viel (LVC_merged, 100% bootstrap support), and a second one splitting into the two genomes from Saint-Doulchard (LSD001.A, LSD023.A; 88% bootstrap support). The British genome of EDI001, however, branches off one SNP ancestral to this polytomy (100% bootstrap support) and possesses one unique SNP. This is remarkable, since the British Isles are one of the most remote places where the First Pandemic was suspected of reaching in relation to its presumed starting point in Egypt.

### Virulence Factor and Deletion Analysis.

**Fig. 3.**
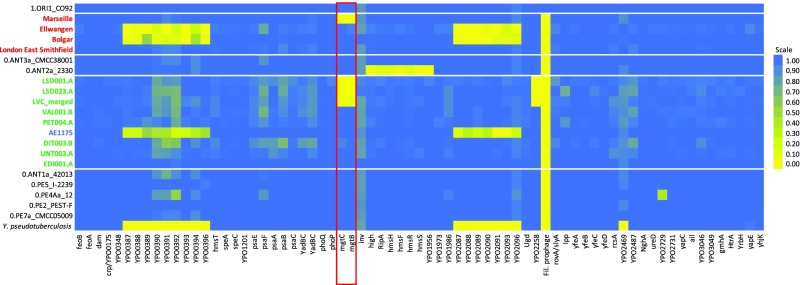
Heatmap showing the percentage of coverage of chromosomal virulence factors. First Pandemic genomes (blue and green) and Second Pandemic genomes (red) are shown in combination with selected strains of main clades of modern *Y. pestis* diversity on branch 0 as well as the reference genomes of *Y. pseudotuberculosis* and *Y. pestis* (CO92).

We screened for the presence/absence of 80 chromosomal and 42 plasmid-associated virulence genes (31, 32) in all First Pandemic genomes with higher than 4.5-fold coverage (Fig. 3 and *SI Appendix*, Fig. S5). Only the filamentous prophage was consistently found absent in all presented genomes. This is expected, since it has integrated into the genome of only a number of modern branch 1 genomes (33). Reduced coverages for a set of virulence factors can be seen in the Altenerding (AE1175), Bolgar, and Ellwangen genomes due to a capture bias, since the capture probe set in the respective studies was designed on the basis of *Y. pseudotuberculosis* rather than of *Y. pestis* (7, 12).

Intriguingly, the most derived First Pandemic genomes from Lunel-Viel (LVC_merged) and Saint-Doulchard (LSD001.A, LSD023.A) show a deletion of two chromosomal virulence-associated genes, *mgtB* and *mgtC* (Fig. 3). These magnesium transporters are part of the PhoPQ regulon, which is important for survival of *Y. pestis* in the magnesium-deficient environment of macrophages. However, functional studies on *mgtB* hint at an important role during macrophage invasion rather than intracellular survival (34).

A second deletion was observed for the gene YPO2258, categorized as a potential virulence factor based on the presence of a frameshift mutation in the avirulent 0.PE2_Microtus91001 strain (32). Its inactivation in the 2.ANT1_Nepal516 strain, isolated from a human patient, nevertheless indicates that this gene is not essential for virulence in humans (35).

Further exploration of the deletion of the two neighboring genes *mgtB* and *mgtC* revealed that they are part of a ∼45-kb deletion (positions 1,883,402–1,928,869 in the CO92 reference), affecting 34 genes including multiple motility (*motA*, *motB*) and chemotaxis genes (*cheA*, *cheB*, *cheD*, *cheR*, *cheW*, *cheY*, *cheZ*) (*SI Appendix*, Fig. S6). On the downstream end, the deletion is flanked by an IS100 insertion element. A potential upstream insertion element might be undetectable at our current resolution due to a genome rearrangement in the reference genome CO92. This is in agreement with previous findings concerning the highly abundant IS100 element in *Y. pestis*, responsible not only for disruptions of multiple genes caused by homologous recombination (29), but also for the loss of the 102-kb-long *pgm* locus containing a high-pathogenicity island in several strains (36). To address the specificity of this deletion to the sixth- to eighth-century strains from France, we also investigated the presence of the two virulence factors in all other modern and ancient strains in this study. Intriguingly, a similar deletion affecting the same region including *mgtB* and *mgtC* was observed in the late Second Pandemic genomes from London New Churchyard [1560–1635 (37)] and Marseille L’Observance [1720–1722 (13)]. However, a full deletion of this 45-kb region was not found in any of the other ancient or modern genomes. Therefore, the deletion appeared independently in the later course of both the First and Second Pandemics. A second but smaller chromosomal deletion and a homoplastic deletion on the pMT1 plasmid are presented in *SI Appendix*, Fig. S7.

## Discussion

### Identifying *Y. pestis* DNA in Low-Complexity Specimens.

In total, we screened 171 samples from 20 sites in France, Germany, and Spain for *Y. pestis* with a qPCR assay (19) and 22 additional samples from Edix Hill, Britain, with the metagenomic tool MALT (20). All putatively positive samples were turned into UDG libraries and subsequently enriched for *Y. pestis*, resulting in mean coverages ranging from 0.01- to 38.1-fold.

The validation of genomic data with relatively low amounts of mapping reads as presented here is challenging; DNA extracted from archaeological remains results in metagenomic data, and differentiating between target organism DNA and environmental background can be difficult.

The identification of *Y. pestis* DNA based on PCR targeting the *pla* locus on the pPCP1 plasmid has theoretically been shown to be problematic (38), leading to discussions about false-positive results (17). However, assignment to *Y. pestis* based on reads retrieved from shotgun sequencing and mapping to a reference genome also can be challenging in case of extremely low genomic coverage (3, 4). Since all of the presented genomes are derived from DNA libraries specifically enriched for *Y. pestis* DNA and are thus biased toward the target organism, a previously suggested competitive mapping approach (3) would not be suitable. Instead, we considered the relative number of mapping reads to the plasmids and chromosome to identify false-positive samples from captured data. We were able to verify that 30 out of 33 samples were positive for *Y. pestis* with as few as 2,000 reads mapping to the chromosome. Since the three plasmids pCD1, pMT1, and pPCP1 were already present in the early divergent Neolithic and Bronze Age strains (3, 4) and loss of plasmids has only been observed sporadically in attenuated strains (39), this method could be reliably applied to data stemming from other branches in the *Y. pestis* phylogeny.

### Analyzing Microdiversity with Low-Coverage Genomes.

Reliable SNP calling is crucial for the phylogenetic analysis of verified low-coverage genomes and can be challenging when dealing with ancient pathogen DNA stemming from metagenomic contexts. This has been demonstrated on *Y. pestis* genomes (7), but previously applied visual inspections are time-consuming and not easily reproducible.

Here, we present an approach for SNP authentication using a semiautomated SNP evaluation. We selected three criteria for our evaluation to assess the likelihood of mismapping. We excluded all SNPs that (A) had higher coverage when mapped with less strict parameters, (B) had “heterozygous” positions in close proximity, or (C) were flanked by gaps. With these filters, we tolerate a loss of specificity (3.59–8.57% true positive erroneously filtered) to reach a maximum sensitivity (100% false positives filtered), as shown with simulated data. Our method is therefore tailored for the reliable characterization of microdiversity. Moreover, the tool “SNPEvaluation” that was newly developed for this analysis offers a highly flexible framework for the assessment of VCF files and can be utilized also for a variety of analyses on different organisms.

### Phylogenetic Analysis.

We were able to confidently reconstruct eight genomes associated with the First Pandemic from Britain, France, Germany, and Spain, providing insights into the microdiversity and persistence of *Y. pestis* in Europe between the sixth and eighth centuries.

Our presented genomes add diversity to a phylogenetic lineage that was previously shown to contain two identical sixth-century genomes from southern Germany [Aschheim and Altenerding (7, 8)]. It diverges between the 0.ANT1, 0.ANT2, and 0.ANT5 clades in the main *Y. pestis* phylogeny and shares a short branch with a second- to third-century genome from the Tian Shan mountains (28). Intriguingly, a single diversification event gave rise to the published as well as three of the presented additional branches, two composed of single genomes with two to three derived SNPs and one branch diversifying into three distinct strains. Similar polytomies can be detected in other parts of the phylogeny of *Y. pestis* that have been related to human epidemics (40): one gave rise to branches 1–4 (including ancient Second Pandemic genomes, Fig. 2*A*) and is dated to 1142–1339 (40), shortly before the European Black Death. To date, it is unknown whether this event was restricted to a rodent reservoir, or if it was already associated with a human epidemic. A second polytomy gave rise to the 1.ORI clade, which includes strains related to the worldwide spread of plague during the Third Pandemic in the 19th century (Fig. 2*C*).

Within the First Pandemic lineage, the genomes that derive from this polytomy display variable terminal branch lengths (1–23 SNPs), which are likely concurrent with their different ages (see below). Given that *Y. pestis* is a pathogen that can cover large geographic distances without accumulating genetic diversity (12), it is challenging to elucidate the geographic origin for this diversification event. A first hypothesis suggests an origin of this diversification event within the historically recorded geographic range of the First Pandemic, i.e., either in Europe, the Mediterranean basin, or the Middle East. Our current data may lend some credibility to this scenario for two reasons: First, we identify four European strains with short genetic distances from this polytomy, the shortest of which is identified in three locations in rural Bavaria, and second, we identify an almost direct ancestor of this polytomy to be present in Europe during the sixth century, represented by a genome from Britain. Alternatively, the bacterium may have been recurrently introduced to the affected regions from a single remote reservoir.

The hypothesis of a single introduction would require the establishment of a local reservoir, since the genomes recovered from Lunel-Viel and Saint-Doulchard are clearly not associated with the initial outbreak in 541–544 but rather with subsequent ones (see below). The establishment of a local reservoir is further substantiated by two diversification events in the French clade, one giving rise to the genome of Lunel-Viel with only one unique SNP and a second event only two SNPs derived, giving rise to both Saint-Doulchard genomes. Possible locations for reservoirs during the First Pandemic have been suggested in the Iberian Peninsula and the Levant (41). There is also a growing body of evidence for the presence of black rats (*Rattus rattus)* in Europe in late Antiquity and the Early Medieval Period (42, 43), suspected to represent the main reservoir species during the Second Pandemic (42).

Such a scenario would be congruent with the Second Pandemic, where the phylogeny of ancient genomes is in line with a single introduction and subsequent persistence in a local host species (12, 37, 44), although this hypothesis was challenged by an alternative scenario claiming multiple introductions on the basis of climatic data (45). Similar to the European Second Pandemic lineage (12, 13), strains emerging from the First Pandemic lineage have so far been recovered solely from ancient DNA of European plague burials, suggesting that the lineage either went extinct or persists in a yet-unsampled reservoir.

### Origin of the Justinianic Plague.

Based on available data, it has been suggested that the most parsimonious location for the divergence event that gave rise to the First Pandemic lineage is Central Asia (28). All published genomes of the branches 0.ANT1, 0.ANT2, and 0.ANT5 that frame the First Pandemic lineage in the phylogenetic tree were sampled in the autonomous Xingjiang region in northwestern China or in Kyrgyzstan (40, 46). In addition, an ancient second- to third-century *Y. pestis* genome from the Tian Shan mountains in Central Asia (28) branches off basal to all the First Pandemic genomes. The resulting claim that the Huns might have brought plague to Europe is, however, unsubstantiated due to the gap of more than three centuries before the onset of the First Pandemic.

Since the long shared branch of the First Pandemic genomes (45 SNPs) does not have any known extant descendants, this strain might have been maintained in a now extinct reservoir after its emergence in Central Asia. The first outbreak is reported in Pelusium, Egypt; an introduction from either Africa or Asia was presumed, given the sudden and dramatic onset of the pandemic. Previous assumptions of an African origin were mainly based on a single deeply diverging 0.PE strain “Angola” (47) and the reports of the Byzantine historian Evagrius Scholasticus, who wrote in his *Ecclesiastical History* that the plague began in “Ethiopia.” However, there are legitimate doubts about the characterization of the “Angola” genome as a genuine African strain (26, 48) and the account of Evagrius has been assessed critically with historical and philological methods (49, 50). For an Asian origin, the sea route via the Red Sea and the Indian Ocean is a plausible scenario since India was well connected by marine traffic with the early Byzantine Empire (41). A suggested alternative scenario would require overland transport from the Eurasian Steppe via Iran to the Red Sea that is, so far, not supported by any data (51). In conclusion, we interpret the current data as insufficient to resolve the origin of the Justinianic Plague as a human epidemic.

### Archaeological and Historical Context.

Here, we present genomic evidence for the First Pandemic reaching the British Isles in the sixth century. This genome was recovered from a burial on the site of Edix Hill, close to Cambridge (Roman *Duroliponte*) and near a Roman road running north from London (*Londinium*) toward Lincoln (*Lindum Colonia*) via Braughing, all of which were Roman settlements. Based on archaeological dating in combination with its rather basal position within the clade, this genome is likely related to the very first occurrence of plague in Britain suggested for 544 (*SI Appendix*), potentially introduced via sea communications with Brittany following the outbreak in central Gaul in 543 (Fig. 1, ref. 52). Interestingly, the genome was recovered from a single burial, underlining that, in small settlements, plague-induced mortality crises need not always involve a radical change in mortuary practice toward multiple or mass burials. The fact that two of the four additional Edix Hill individuals that appeared positive for plague in the MALT screening were buried in two simultaneous double burials nevertheless suggests that otherwise broadly typical cemeteries where multiple burials are relatively frequent are indeed a good indicator for epidemic events (18).

In addition, we were able to reconstruct four genomes from the Mediterranean basin and central France, where the historical records are more explicit about the presence of plague during the First Pandemic. Regarding Spain, the radiocarbon dating of the *Y. pestis*-positive individual from Valencia (432–610) would include the first outbreak reported for Spain in 543 in a contemporary chronicle (*SI Appendix*). The three unique SNPs identified in this genome, which separate it from the identified polytomy, however, may suggest its association with a later outbreak. Intriguingly, a canon of a church council held in 546 in Valencia dealing with burial practices for bishops in case of sudden death was recently connected with plague by philological and contextual analysis (53). Later outbreaks within the relevant time frame are documented in Spain’s Visigothic kingdom, e.g., in 584 and 588 by Gregory of Tours, and by a funerary inscription dated 609 at Cortijo de Chinales 35 km northeast of Malaga (*SI Appendix*).

The second Mediterranean genome from Lunel-Viel in southern France represents another and significantly younger outbreak, since it belongs to an independent clade that derives from the same polytomy as the Spanish and German genomes, sharing 12 SNPs with the genomes of Saint-Doulchard and possessing 1 unique SNP. The radiocarbon dates for the inhumations give an interval of at least 567–618 (youngest lower and oldest upper boundary; *SI Appendix*, Table S13) overlapping with documented outbreaks in 571, 582, 588, 590, and possibly 599–600 in southern France (Fig. 1 *A* and *C*). Lunel-Viel’s broader vicinity includes Arles, the seaport city of Marseille, and the Rhône mouth. Close to important coastal and fluvial shipping routes as well as Roman roads that facilitated the spread of plague (41), Lunel-Viel could have been affected by all five recorded epidemics. The initial outbreak, documented for Arles ca. 543, falls outside of some of the radiocarbon intervals. This is consistent with the phylogenetic analysis that shows a higher accumulation of SNPs in this genome. Thus, the victims at Lunel-Viel can most likely be attributed to an outbreak in the last quarter of the sixth century.

Within the site of Saint-Doulchard, two distinct genomes were found, one of which is represented by only one sample (LSD001.A), and the other is present in seven, including the sample LSD023.A with the highest coverage. The presence of two independent genomes in the same site, i.e., without one of them being directly ancestral to the other, has so far not been reported for the First or the Second Pandemic. Furthermore, the similar branch lengths of seven and nine SNPs derived from a common node do not allow for a clear temporal distinction. Also based on the stratigraphy of the site, the temporal structure cannot be fully resolved: all 11 plague-positive burials are dug into a trench that must have been open over the whole course of these inhumations. However, since the individuals were buried in distinct graves, they cannot be clearly assigned to a single event. Therefore, this finding can be explained by two hypotheses: First, the two strains might have struck the local population at the same time in a single outbreak; therefore, the victims were buried indiscriminately. This, however, would have implications for the epidemiology of *Y. pestis*, showing the parallel presence of different strains in a single outbreak. Second, the two strains could belong to two independent outbreaks within a shorter period of time, so the local community returned to the same structure, i.e., the trench, for emergency burials. Regarding the radiocarbon dating of adjacent burials within the trench, ca. 650–880, the closest historically reported outbreak is in Narbonne in 693. This would correspond with the relatively derived state of the two Saint-Doulchard strains compared with all other First Pandemic genomes. Other outbreaks in the West such as 663–666 and 684–687 in the British Isles, 707–709 in Spain, or 680 and 745–746 in Italy, might have been spatially limited and might not have spread to central France. Finally, an anecdote by Gregory of Tours in his sixth *Liber Historiarum* reports how the city of Narbonne was struck by plague repeatedly between 582 and 584, claiming the lives of those who fled the city when they returned to it. Although this episode is too early to account for the two strains in Saint-Doulchard, it showcases how a city was struck by plague multiple times over a short interval, as proposed in our second hypothesis.

In Bavaria, Germany, we detected *Y. pestis* in four sites (Dittenheim, Petting, Unterthürheim, Waging) in addition to the two previously published sites [Altenerding (7), Aschheim (8)]. Two of the reconstructed genomes were identical to Altenerding and Aschheim, suggesting that these four can be attributed to the same epidemic event. Some of the radiocarbon intervals of these sites fall even slightly before the onset of the First Pandemic, suggesting an association of this outbreak directly with the Justinianic Plague. Regarding the Edix Hill genome, this would in turn necessitate the accumulation of one (Edix Hill) to two (Altenerding cluster) SNPs within the onset of the First Pandemic between 541 and 544.

Intriguingly, the genome of Petting, Bavaria, falls not with the Altenerding cluster but in a distinct phylogenetic position. Since this strain also branches off from the common node with the other Bavarian strain as well as the French and Spanish genomes, this shows the presence of two independent strains and, therefore, presumably two independent epidemic events in early medieval Bavaria. This is striking, since we lack any historical records of the First Pandemic affecting southern Germany. The radiocarbon dates for the Bavarian sites are inconclusive and do not allow for a clear temporal separation of the two events. The higher number of accumulated SNPs nevertheless suggests a younger date for the epidemic represented by Petting. Further phylogeographic analyses are presented in *SI Appendix*.

### Deletion Analysis.

The analysis of virulence factors revealed a deletion of a ∼45-kb region in the most derived and most recent genomes thus far identified for the First Pandemic. This deletion contained two previously described virulence factors involved in host cell invasion and intracellular growth (*mgtB* and *mgtC*). Intriguingly, a similar deletion covering the same genomic region was detected in the most derived available Second Pandemic genomes from London New Churchyard (1560–1635) and Marseille (1720–1722). Genome decay by deletion or pseudogenization is a well-known trait of *Y. pestis* and has contributed to its distinct ecology and pathogenicity (54). Both deletions from the First and Second Pandemics are observed in genomes recovered from human victims. Therefore, it is reasonable to assume that the deletion may not have reduced the bacterium’s virulence. Moreover, it affects a number of cell surface proteins—remnants of the motile lifestyle of nonpestis *Yersiniae* (55)—so the deletion might have even facilitated immune evasion.

Because none of the investigated modern strains harbored this specific deletion, this possible case of convergent evolution might be an adaptation to a distinct ecological niche in Europe or the Mediterranean basin since an ancient local reservoir is the most parsimonious hypothesis for both historical pandemics (13, 44).

### Concluding Remarks.

Our study offers insights into the first historically documented plague pandemic, complementing the limited power of conventional historical, archaeological, or paleoepidemiological research. Moreover, we show the potential of paleogenomic research for understanding historical and modern pandemics by a comparative approach on genomic features across millennia. Facing the problem of low-coverage genomic data with a high environmental background—a notorious challenge in ancient DNA research—we have developed approaches to facilitate the authentication and confident phylogenetic placement of such genomes.

In the future, more extensive sampling of putative plague burials will help to draw a more comprehensive picture of the onset and persistence of the First Pandemic, especially on sites in the eastern Mediterranean basin, where not only is the Justinianic Plague reported to have started, but where also the eighth century outbreaks clustered according to the written records presently available. This will contribute to the comparative exploration of *Y. pestis*’ microevolution and human impact in the course of past and present pandemics.

## Materials and Methods

### Sites and Samples.

The acquisition and selection of samples followed two approaches: Focusing on Bavaria, we concentrated on one region, where the two previously reconstructed *Y. pestis* genomes attributed to the Justinianic Plague had been found (7, 8). Additionally, given the absence of robust genetic evidence from Gaul and the Mediterranean basin, which the surviving historical records depict as the epicenter of the pandemic, and the controversial presence of plague on the British Isles during the Justinianic Plague, we extended our screening to four sites with multiple burials in a broader geographical scope on the Mediterranean coast in France and Spain, central France, and inland Britain. Table 1 gives an overview of all tested sites.

For the first focus, we collected samples of 79 individuals from 46 burials belonging to 16 archaeological sites in Bavaria, Germany, and one site in Austria (Fig. 1*B*). Importantly, the dating of the burials spans the 4th to 10th century, including also burials dating before (8 individuals on three sites) and after (17 individuals on five sites) the Justinianic Plague (541–544). Since mass graves that could be indicative of an epidemic are unsurprisingly rare for the small settlements associated with early medieval cemeteries in Bavaria, we followed the approach of the previous successful studies (7, 8, 18): we systematically screened multiple burials, i.e., where two or more individuals were found in a context indicating a simultaneous burial, such as a common grave pit and articulated remains on the same level. Single burials were sporadically tested, if the context suggested a close connection to a multiple burial. Burials with indications of a violent death of the interred were excluded, since a coincidental acute infection with *Y. pestis* seems unlikely.

Within the Mediterranean basin, we tested inhumations from Valencia, Spain, and Lunel-Viel (Hérault), France. A contemporary chronicler records that bubonic infection devastated Spain during the first phase of the Justinianic Plague (541–544), and a recently published interpretation of a contemporary record argues that it reached Valencia presumably before 546 (53). Further textual references, including an epitaph dating to 609, document later Iberian outbreaks (56) (Fig. 1). In the Visigothic levels of the *Plaça de l’Almoina* in Valencia, several collective burials in an intramural cemetery were interpreted as possible plague burials (56, 57).

The historical evidence for the First Pandemic in France is more substantial, mainly based on the contemporary bishop and historian Gregory of Tours (58). He reports several plague outbreaks spanning from ca. 543 in the province of Arles through 588 in Marseille to 590 in Avignon (Fig. 1*C*). The site of Lunel-Viel, around 30 km southwest of the ancient Roman city of Nîmes and less than 100 km from the mentioned cities, revealed eight exceptional inhumations in demolition trenches unrelated to the nearby contemporary cemeteries (59).

In central France, we screened material from the site Le Pressoir in Saint-Doulchard, close to Bourges. Gregory of Tours (d. 594), explicitly mentions an outbreak at Bourges only in 571. Surviving written records are scarce leaving it undocumented whether other outbreaks in southern Gaul such as those just mentioned or the 693 outbreak in Narbonne reached Bourges (Fig. 1*C* and *SI Appendix*). The use of an existing ditch, most likely intended as an enclosure for the cemetery, as funerary space, gave however a first indication of a local mortality crisis, which was further substantiated by the presence of multiple burials and the demographic profile (60). From the 48 burials within the trench, 26 samples were selected mainly based on preservation, including 9 samples of multiple burials.

For the British Isles, the historical evidence for plague presence in the sixth century is controversial. Unlike later outbreaks in seventh-century Britain that are reported, e.g., by Bede, the identification of a disease occurring in the 540s and called *blefed* in Irish chronicles as bubonic plague, is mainly based on the coincidence with the Continental European outbreaks and thus uncertain. The same is true for Britain, where a great mortality (*mortalitas magna*) is reported in the *Annales Cambriae* (*SI Appendix*). For this study, we screened 22 individuals from the Anglo-Saxon cemetery of Edix Hill, well-connected to the Roman road network and Roman towns, and characterized by a number of multiple burials.

For the screening, one tooth (preferentially molar) per individual was used for every individual of a multiple burial, if available. For a number of individuals, additional teeth were tested, if sequencing the first gave a weak positive. For the collective burials from Valencia, a clear attribution to individuals was not assured, so multiple teeth were sampled per feature number, where possible. Detailed site descriptions can be found in *SI Appendix*, including a table with all screened samples (*SI Appendix*, Table S1).

Details on the radiocarbon dating and the cartography of the presented maps are described in separate sections of the *SI Appendix*.

### Sample Preparation, DNA Extraction, qPCR, and MALT Screening.

The sample preparation and DNA extraction for samples from Austria, France, Germany, and Spain were done in the ancient DNA facilities of the ArchaeoBioCenter of the Ludwig Maximilian University Munich, Germany, and the Max Planck Institute for the Science of Human History in Jena, Germany.

All teeth were cut along the cementoenamel junction, and the surface of the pulp chamber was drilled out with a dental drill from the crown and in some cases the root, aiming for 30–50 mg of bone powder. DNA was extracted based on the protocol published in ref. 61: The powder was suspended in 1 mL of extraction buffer (0.45 M EDTA pH 8.0, and 0.25 mg/mL proteinase K in UV-irradiated HPLC water) and incubated at 37 °C overnight on a rotor. After centrifugation, the supernatant was mixed with 10 mL of binding buffer (5 M guanidinium hydrochlorid, 40% isopropanol, and 90 mM sodium acetate) to bind the DNA on a silica column of either the MinElute purification kit (Qiagen) or the High Pure Viral Nucleic Acid Kit (Roche). After purification with washing buffer of the respective kit, the DNA was eluted in 100 µL of TET buffer (10 mM Tris⋅HCl, 1 mM EDTA, pH 8.0, 0.05% Tween 20).

All extracts were tested with the qPCR assay targeting a 52-bp region on the pPCP1 plasmid published in ref. 19 with minor changes (0.75 mg/mL BSA, additional 5% DMSO, EVA green instead of SYBR green, annealing for 30 s, elongation for 30 s, gradient from 60 to 90 °C). All samples showing an amplification with a melting peak between 74 and 80 °C were captured for *Y. pestis*.

The samples of Edix Hill, Britain, were prepared in the ancient DNA facility of the University of Cambridge, Department of Archaeology. Root portions of teeth were removed with a sterile drill wheel. These root portions were briefly brushed with 5% (wt/vol) NaOCl using a UV-irradiated toothbrush that was soaked in 5% (wt/vol) NaOCl for at least 1 min between samples. Roots were then soaked in 6% (wt/vol) bleach for 5 min. Samples were rinsed twice with ddH_2_O and soaked in 70% ethanol for 2 min, transferred to a clean paper towel on a rack inside the glove box, UV irradiated for 50 min on each side, and then allowed to dry. They were weighed and transferred to clean, UV-irradiated 5-mL or 15-mL tubes for chemical extraction. Per 100 mg of each sample, 2 mL of EDTA Buffer (0.5 M, pH 8.0) and 50 μL of proteinase K (10 mg/mL) were added. Tubes were rocked in an incubator for 72 h at room temperature. Extracts were concentrated to 250 μL using Amplicon Ultra-15 concentrators with a 30-kDa filter. Samples were purified according to manufacturer’s instructions using the Minelute PCR Purification Kit with the only change that samples were incubated with 100 μL of Elution Buffer at 37 °C for 10 min before elution.

### Library Preparation.

Of putatively positive extracts in the qPCR or MALT screening, 50 µL were turned into Illumina double-stranded DNA libraries with initial USER treatment (New England Biolabs) to remove postmortem damage in form of deaminated cytosines by consecutive incubation with uracil-DNA-glycosylase (UDG) and endonuclease VIII (25). To enhance the efficiency of subsequent double indexing, UDG-treated libraries were quantified by qPCR using IS7/IS8 primer and split for a maximum of 2 × 10^10^ DNA molecules. Every library was indexed with a unique index combination in a 10-cycle amplification reaction using Pfu Turbo Cx Hotstart DNA Polymerase (Agilent) (62, 63). The amplification products were purified using the MinElute DNA purification kit (Qiagen) and eluted in TET (10 mM Tris⋅HCl, 1 mM EDTA, pH 8.0, 0.05% Tween 20). For the capture, the indexed libraries were amplified to 200–300 ng/µL using Herculase II Fusion DNA Polymerase (Agilent) and purified a second time as described.

The non-UDG library preparation for all Edix Hill samples was conducted using a protocol modified from the manufacturer’s instructions included in the NEBNext Library Preparation Kit for 454 (E6070S; New England Biolabs) as detailed in ref. 64. DNA was not fragmented and reactions were scaled to half volume; adaptors were made as described in ref. 62 and used in a final concentration of 2.5 µM each. DNA was purified on MinElute columns (Qiagen). Libraries were amplified using the following PCR setup: 50-µL DNA library, 1× PCR buffer, 2.5 mM MgCl_2_, 1 mg/mL BSA, 0.2 µM in PE 1.0, 0.2 mM dNTP each, 0.1 U/µL HGS Taq Diamond, and 0.2 µM indexing primer. Cycling conditions were as follows: 5 min at 94 °C, followed by 18 cycles of 30 s each at 94 °C, 60 °C, and 68 °C, with a final extension of 7 min at 72 °C. Amplified products were purified using MinElute columns and eluted in 35 µL of EB. Samples were quantified using Quant-iT PicoGreen dsDNA kit (P7589; Invitrogen Life Technologies) on the Synergy HT Multi-Mode Microplate Reader with Gen5 software.

### In-Solution Capture.

For the in-solution capture, a probe set was generated using a fragment size of 52 bp and a tiling of 1 bp with the following genomes as templates: CO92 chromosome (NC_003143.1), CO92 plasmid pMT1 (NC_003134.1), CO92 plasmid pCD1 (NC_003131.1), KIM 10 chromosome (NC_004088.1), Pestoides F chromosome (NC_009381.1), and *Y. pseudotuberculosis* IP 32953 chromosome (NC_006155.1). The capture was performed as previously described (65) on 96-well plates with a maximum of two samples pooled per well and all blanks with unique index combinations in one well.

### Sequencing and Data Processing.

All captured products were sequenced either on an Illumina NextSeq500 or HiSeq4000 platform at the Max Planck Institute for the Science of Human History in Jena, Germany. The non-UDG libraries of Edix Hill samples were sequenced on Illumina NextSeq500 at the University of Cambridge Biochemistry DNA Sequencing Facility, and the FastQ files were processed on the Estonian Biocenter server and screened with MALT (20) using a reference set including full bacterial and viral genomes with 85% identity.

For all sequenced UDG libraries, de-multiplexed reads were processed with the EAGER pipeline (66) starting with Illumina adapter removal, sequencing quality filtering (minimum base quality of 20) and length filtering (minimum length of 30 bp). Sequencing data of paired-end and single-end sequencing were concatenated after adapter removal and merging. The same was done for samples from the same individual (DIT004) and all data from Lunel-Viel (LVC) due to low genomic coverage after ensuring an identical genotype. The sequencing results are shown in *SI Appendix*, Table S3.

Mapping against reference genomes of CO92 (chromosome NC_003143.1, plasmid pMT1 NC_003134.1, plasmid pCD1 NC_003131.1, plasmid pPCP1 NC_003132.1) was done with BWA using stringent parameters (−n 0.1, −l 32). Reads with low mapping quality were removed with Samtools (−q 37), and duplicates were removed with MarkDuplicates. For the plasmids, a merged reference was used, consisting of the CO92 reference of pCD1 (NC_003131.1), pMT1 (NC_003134.1), and pPCP1 [NC_003132.1, with base pairs 3,000–4,200 masked (19)], to avoid overestimation of coverage due to homologous regions. For the verification of positive qPCR results, we normalized the number of reads mapping to each plasmid with reads mapping to the chromosome and calculated the Mahalanobis distance for each sample to detect outliers. Based on this, we excluded the samples PEI001.A and DIR002.A as false positives (*SI Appendix*, Table S2).

The raw data of the Aschheim and Altenerding genomes were processed identically, however considering only the A120 sample for Aschheim instead of the combined A120+A76 data (7, 8).

### SNP Calling and Evaluation.

All genomes recovered from UDG-libraries with higher than 4.5-fold mean coverage including the Altenerding genome were assessed in the SNP analysis. Additionally, the sample WAG001.A was evaluated to explore its phylogenetic position, since it was the only positive sample of the relevant site.

The UnifiedGenotyper within the Genome Analysis Toolkit was used for SNP calling and creating VCF files for all genomes, using “EMIT_ALL_SITES” to generate calls for all positions in the reference genome. For the subsequent analyses, 233 previously published modern *Y. pestis* genomes (*SI Appendix*, Table S12), one genome from second- to third-century Tian-Shan mountains [DA101 (28)], one genome representing the Black Death from London East Smithfield [8291-11972-8124 (13)], and seven Second Pandemic genomes [Ellwangen; Bolgar; Marseille L’Observance OBS107, OBS110, OBS116, OBS124, OBS137 (12, 13)] were taken along together with *Y. pseudotuberculosis* (IP32953) as an outgroup. Previously identified problematic regions (26, 40) as well as regions annotated as repeat regions, rRNAs, tRNAs, and tmRNAs were excluded for all following analyses. MultiVCFAnalyzer, version 0.85 (67), was used for generating a SNP table with the following settings: Minimal coverage for base call of 3 with a minimum genotyping quality of 30 for homozygous positions, minimum support of 90% for calling the dominant nucleotide in a “heterozygous” position. All positions failing these criteria would be called “N” in the SNP table. For the SNP evaluation, all N positions of unique SNPs within the First Pandemic lineage were reevaluated, replacing N by “0” for not covered and lowercase letters for homozygous positions with maximum twofold coverage. To test for possible mixed infections or elevated contamination, all SNPs not passing the 90% threshold were plotted (*SI Appendix*, Fig. S1).

For the evaluation of unique and shared SNPs of First Pandemic genomes retrieved from UDG-treated libraries, we used the newly developed tool “SNPEvaluation” (https://github.com/andreasKroepelin/SNP_Evaluation) and a comparative mapping, using BWA with high stringent (−n 0.1, −l 32) and low stringent (−n 0.01, −l 32) mapping parameters, allowing for more mismatches in the latter. SNPs were called true positive when meeting the following criteria within a 50-bp window: (A) the ratio of mean coverage of low-stringent to high-stringent mapping is not higher than 1.00, (B) no “heterozygous” positions, and (C) no noncovered positions (*SI Appendix*, Tables S8 and S9). An assessment of this method is presented in *SI Appendix*, showing a maximal sensitivity (100% false positives detected) while accepting a high specificity (up to 3.49–8.57% of true positions filtered out).

SNP evaluation on the plasmids was done using the same criteria after mapping to the individual references as described above. For the SNP effect analysis, the remaining unique true SNPs were compared with the genome annotations of the CO92 *Y. pestis* reference genome (*SI Appendix*, Table S10).

Shared SNPs (*SI Appendix*, Table S11) were evaluated with the same criteria with minor modifications: The minimum threshold for calling a position was set to one read covering and SNPs were called true positive, if the SNP passed the criteria in more than half of the genomes under examination.

The Aschheim genome was evaluated separately (*SI Appendix*, Table S14) but with the same criteria. As previously addressed (7), the enormously high number of potential false-positive SNPs might not be explained solely by contamination by soil bacteria or sequencing errors but additionally by PCR or capture artifacts.

### Phylogenetic Analyses.

For the phylogenetic analyses, we aimed for one high coverage genome per site to minimize missing data in the SNP alignment, excluding the genome of EDI003.A, EDI004.A, and UNT004.A after assuring no conflicting positions with EDI001.A and UNT003.A, respectively, in the SNP evaluation. A maximum-likelihood tree [RAxML 8 (30) using the GTR substitution model, Fig. 2*A*; for full tree, see *SI Appendix*, Fig. S4] was generated without exclusion of missing and ambiguous data (full SNP alignment), resulting in a total number of 6,580 SNPs. Robustness of all tree nodes was tested by the bootstrap methods using 1,000 pseudoreplicates.

A detailed tree of the First Pandemic lineage was drawn manually based on the performed SNP evaluation, excluding all potential false-positive SNPs (Fig. 2*B*).

### Analysis of Virulence Factors and Genome Decay.

The presence/absence analysis for genes was performed with BEDTools (68) by calculating the percentage across each gene using the function “coverage,” which calculates the percentage of bases in a given window being covered by at least one read (3). Since gene duplications can affect the mapping quality, the mapping quality filter of BWA was set to 0 (−q 0) to generate a bam-file as input. For the heatmap of virulence factors (Fig. 3), a collection of proven and putative virulence genes (31, 32) was evaluated. With this method, only deletions and pseudogenization by large gene truncations can be detected; a test for pseudogenization by frameshift mutations was not attempted. The more extensive analysis on genome decay was based on the annotation file for the reference genome CO92 (55) by extracting all regions annotated as “gene.” For the exact determination of the start and end positions of deletions, mapping with BWA-MEM was performed (69).

## Supplementary Material

Supplementary File
