# Supplementary material for: Ancient Yersinia pestis genomes from across Western Europe reveal early diversification during the First Pandemic (541–750)
Source: Proc Natl Acad Sci U S A. 2019 Jun 4;116(25):12363–72. doi: 10.1073/pnas.1820447116 (PMC6589673; doi:10.1073/pnas.1820447116)
Supplement: Supplementary File [file pnas.1820447116.sapp.pdf]

PNAS
www.pnas.org

Supplementary Information for

Ancient Yersinia pestis genomes from across Western Europe reveal early diversification during the First Pandemic (541–750)

Marcel Keller, Maria A. Spyrou, Christiana L. Scheib, Gunnar U. Neumann, Andreas Kröpelin, Brigitte Haas-Gebhard, Bernd Päffgen, Jochen Haberstroh, Albert Ribera i Lacomba, Claude Raynaud, Craig Cessford, Raphaël Durand, Peter Stadler, Kathrin Nägele, Jessica S. Bates, Bernd Trautmann, Sarah Inskip, Joris Peters, John E. Robb, Toomas Kivisild, Dominique Castex, Michael McCormick, Kirsten I. Bos, Michaela Harbeck, Alexander Herbig, Johannes Krause

Corresponding authors:
Marcel Keller, marcel.keller@ut.ee
Michaela Harbeck, harbeck@snsb.de
Alexander Herbig, herbig@shh.mpg.de
Johannes Krause, krause@shh.mpg.de

This PDF file includes:

Supplementary Information Text
Figs. S1 to S10
Tables S1 to S14
References for SI

www.pnas.org/cgi/doi/10.1073/pnas.1820447116

Supplementary Information Text

Assessment of SNPEvaluation

To test the validity of the tool SNPEvaluation and the applied criteria for detection of false positive SNPs, we generated a set of artificial datasets representing clean and contaminated *Y. pestis* genomes ranging from ~3x to ~30x coverage. To mimic the contamination with environmental bacteria (background), we used the sample DIR002.A, since enrichment with a *Y. pestis* probeset cannot be simulated reliably *in silico*. The sample DIR002.A was captured and sequenced after initial qPCR screening, but was classified as negative for *Y. pestis* after sequencing and mapping. Therefore, the 8470 reads mapping with strict parameters are derived from environmental sources. For the target *Y. pestis* reads (foreground), we generated artificial sequencing data based on the reference genome CO92 aiming for 3-fold, 5-fold, 10-fold and 30-fold coverage in triplicates using the tool gargammel (1). We simulated paired-end 75 bp sequencing reads using the coverage flag (-c) and the length distribution of the paired-end 75 bp sequenced sample DIT003.B. The achieved coverages are ~6 % lower (on average 2.83-fold, 4.72-fold, 9.43-fold, 28.20-fold, see SI Appendix, Table S5) due to mapping quality filtering. The artificial reads were combined with the background and mapped with stringent (-n 0.1) and lenient (-n 0.01) mapping parameters. Since the capture probe set was constructed based on CO92 among others, a possible capture bias was neglected for the artificial CO92 reads.

To test for the sensitivity of our method, we applied it to all false positive SNPs that were introduced by our background sample (SI Appendix, Fig. S2, Table S6). As expected, no SNPs were called in any of the simulated *Y. pestis* datasets without background. Using only the background (DIR002), a total number of 451 SNPs were called. One additional SNP was only called with spiked in *Y. pestis* reads (called as N in DIR002), summing up to a total of 452 possible false positive SNPs. With increasing coverage of spiked in *Y. pestis* reads, the number of false positive SNPs decreases from on average 139.67 for 3-fold, 40.33 for 5-fold, 5 for 10-fold to 0 for 30-fold, showing that the environmental contamination can introduce a significant number of false positive SNPs for low coverage samples but is negligible for high coverage samples. The remaining positions of putative false positive SNPs are called as heterozygous (N) or as reference call in high coverage samples (in average 0.67 for 10-fold, 63.67 for 30-fold).

Using the same criteria as for the First Pandemic samples in this study, SNPEvaluation was able to filter 100 % of false positive SNPs from every artificial dataset. Moreover, all chosen filtering criteria were necessary for the determination as false positive SNPs: Regarding the ratio of mean coverage with low/high stringent mapping, this criterion was able to filter out almost all SNPs. Only 2 of 451 SNPs from the pure background, 1 of 147 SNPs from a 3x-fold and 1 of 39 SNPs from a 5-fold coverage contaminated *Y. pestis* samples passed the criterion (ratio 1.00), but were filtered out either due to a heterozygous SNP or uncovered positions surrounding the SNP. The lowest ratio for the false positive SNPs without other criteria

applying ranges between 1.05 and 1.21, justifying the very strict criterion of a ratio of 1.00 to be accepted as a true positive SNP.

To determine the specificity of our method, we generated a SNP table based only on modern *Y. pestis* strains and extracted the SNPs that are shared by at least 34 genomes (~15 %). This cutoff was chosen due to the local maximum in the distribution of the numbers of genomes sharing SNPs. This resulted in a list of 418 SNPs, which constitute the backbone phylogeny of *Y. pestis* and are a good proxy for true positive SNP positions (see SI Appendix, Fig. S3, Table S7). However, all positions appear as reference calls in our datasets, since the artificial reads are based on the same genome (CO92) that was arbitrarily chosen as reference genome.

In the simulated CO92 datasets without background, in average 79.33 positions were called as reference for the simulated 3-fold coverage samples, 230.33 for 5-fold, 403.33 for 10-fold and all 418 for 30-fold. All remaining positions were called as N due to low coverage. The ratio of mean coverage with low/high stringent mapping never exceeded 1.00 in any datasets, showing that sequencing errors simulated by gargammel are not interfering with this parameter. A maximum of 2.63 % of the reference calls were filtered out due to uncovered bases surrounding the position in the simulated 3-fold or 5-fold coverage samples, but never for 10-fold or 30-fold coverage. Using solely the background sample, only 2 positions were called as reference but filtered out due to a >1.00 ratio, giving evidence that the DIR002 sample is indeed negative for *Y. pestis*.

For the simulated CO92 datasets contaminated with the background, in average 74.33 positions were called for the 3-fold samples, 227.67 for 5-fold, 401.33 for the 10-fold and all 418 for 30-fold. This means that 0 – 7.89 % of the positions are not called due to heterozygosity introduced by the background. After filtering with SNPEvaluation, in average 69.67 positions remained for the 3-fold samples, 216.67 for 5-fold, 385.00 for 10-fold and 401.67 for 30-fold, so 3.59 – 8.57 % of called positions are filtered out.

In summary, SNPEvaluation offers a maximum sensitivity (100 % of false positive SNPs filtered out), while retaining a high specificity (8.57 % or fewer of true shared positions filtered out). Although we used only one background sample in this analysis and were not able to model, i.e., the capture bias, the presented method is a powerful tool to filter low coverage datasets for false positive SNPs, a crucial step to build reliable bacterial phylogenies.

SNP Evaluation of the Aschheim Genome and SNP Effect Analysis

The Aschheim genome (2) was evaluated separately, given its peculiarly high number of potential false positive SNPs described previously (3). Our systematic evaluation verified previous classifications: all SNPs potentially unique to Aschheim that passed the criteria show a coverage lower than 5-fold, which was the threshold of their SNP calling. However, the high number of presumably shared SNPs that did not pass our stricter criteria underlines again the high 'heterozygosity' of the genome (see SI Appendix, Table S14) that might be explained not only by contamination by soil bacteria or sequencing errors but presumably also by PCR and capture artefacts, as previously discussed (3). Therefore, the Aschheim genome was excluded from subsequent analyses.

Of the 39 non-shared phylogenetically informative chromosomal SNPs that were detected among all new genomes, 20 are non-synonymous in coding regions of (hypothetical) proteins (SI Appendix, Table S10). The genome of VAL001.B shows non-synonymous SNPs in the genes *tyrP*, YPO1985 and YPO2588. TyrP is a transcriptional regulator for the metabolism of aromatic amino acids and was identified as a virulence factor crucial for the infection of mice (4). YPO1985 was identified as a glycosyl transferase gene inactivated in the avirulent strain 91001 and thus might be a virulence factor as well (5). The gene YPO2588 codes for an ABC transport protein. An additional non-synonymous SNP was detected on the pMT1 plasmid in the putative DNA-binding protein YPMT1.59C. For the genome PET004.A, only one non-synonymous SNP was identified, located on the hypothetical protein YPO3510. The genomes of LVC_merged, LSD001.A and LSD023.A show seven shared non-synonymous SNPs: in the genes *marC*, a multidrug resistance protein; *phrB,* coding a 3',5'-cyclic-nucleotide phosphodiesterase; *tyrA*, a bifunctional chorismite mutase/prephenate dehydrogenase; the phosphoenolpyruvate carboxylase *ppc,* the oligogalacturonate lyase ogl; the sugar transport ATP-binding protein YPO1554, identified as pseudogene in CO092 (6); and the hypothetical protein YPO4112. The gene *ppc* has been shown to be connected with the type III secretion system that is essential for pathogenicity in *Y. pestis* by injection of Yops (*Yersinia* outer membrane proteins) into host cells of the innate immune system (7). An additional non-synonymous SNP shared among this clade was found on the pCD1 plasmid, affecting *yopB*, a *Yersinia* outer membrane protein, identified as virulence factor since it is an important translocon for the injection of effector proteins via the type III secretion system (8). A SNP unique to LVC_merged was identified in the hypothetical protein YPO2238. Both LSD001.A and LSD023.A share a SNP in the *lacY*, a galactoside permease. In LSD001.A, four unique SNPs were identified as non-synonymous: in *hisP*, a histidine/lysine/arginine/ornithine transporter subunit; *yapB*, a putative autotransporter protein pseudogenized in KIM (9); YPO1999, a decarboxylase; and YPO1856, a hypothetical protein. In LSD023.A, three non-synonymous SNPs were found: in *cpxA*, a two-component sensor kinase; *lacY*, a galactoside permease already affected by a SNP shared with LSD001.A; and *znuC*, a high-affinity zinc transporter ATPase.

Extended Deletion Analysis

A deletion of ca. 12.9 kb was found on the chromosome (positions 2,533,444 to 2,546,401 in CO92) of all three genomes of the French clade (LVC_merged, LSD001.A, LSD023.A), affecting the genes *araF, araG, araH, araC, manA* and several hypothetical proteins (see SI Appendix, Fig. S7). Affecting the arabinose operon, strains with this deletion will be unable to metabolize arabinose. This is also a defining characteristic of the biovar *Microtus* (10), here caused by a 122-bp frameshift deletion in *araC*, a clade within the 0.PE4 branch including the genomes 0.PE4Ca_CMCCN010025, 0.PE4Cc_CMCC18019, 0.PE4Cd_CMCC93014, 0.PE4Ce_ CMCC91090, 0.PE4m_I-3086, 0.PE4_I-3134, 0.PE4_Microtus91001 and 0.PE4_M0000002. The loss of the arabinose operon is however only one of multiple changes in genome architecture, gene loss and pseudogenization in this clade, causing these strains to

be avirulent in humans. Therefore, the 12.9 kb deletion observed here in the First Pandemic genomes is presumably a similar case of genome decay.
Another deletion of 14.8 kb was found on the pMT1 plasmid (positions 23,133 to 37,975 in CO92) of the genomes of the French clade (LVC_merged, LSD001.A, LSD023.A) and both genomes of Unterthürheim (UNT003.A, UNT004.A). All 21 genes affected by this deletion are hypothetical proteins including a putative ABC transporter ATP-binding protein, therefore the functional consequences of the deletion cannot be determined. The fact that this deletion is found in the French clade as well as in the Unterthürheim genomes but not in the Altenerding (AE1175) and Dittenheim genomes (DIT003.B) that are otherwise identical, suggests that this deletion is more common and mediated by a process similar to the transposable elements on the chromosome.

Phylogeographic Analyses

The new genomes and radiocarbon dates combined suggest an association of the British genome as well as the polytomy giving rise to the four lineages with the early phase of the First Pandemic or even the Justinianic Plague itself (541–544). The accumulation of one (EDI001) or two (Altenerding cluster) SNPs from the basal node of all genomes could have happened on the way from Egypt to western Europe. The fact that the pandemic reportedly spread from Pelusium along the Mediterranean coastline in two independent waves, one heading west to Alexandria and the other east to Palestine, could explain the early branching event (11). Strikingly, such a diversification during the onset of a pandemic has not been found yet for the Black Death (1348–1352), where the two genomes from London East Smithfield (12) and Barcelona were found to be identical (13). Besides differing mutation rates, this might be due to differences in propagation speed between the 6th and 14th century, related to changes in human mobility by land and sea: a significantly slower or less direct transmission over large distances would allow the pathogen to acquire more substitutions.
The lineages found in Bavaria could have spread there by a ‘western route’ from Gaul, by a ‘southern route’ from Italy or by an ‘eastern route’ from Illyricum, which were all affected by plague in or around 543. The presence of plague in the British Isles even suggests a fourth ‘northern route’ upstream along the Rhine river. The ‘western’ and ‘southern route’ would have necessitated overland transport via the Roman road network that connected all of the relevant sites with the Mediterranean coastlines and was still functional in the 6th century (SI Appendix, Fig. S9). The ‘southern route’ would have required crossing the Alps via different passes that had been used since Antiquity (14). Navigation along the Danube could have facilitated the ‘eastern route’. The importance of rivers for the spread of plague has already been shown exemplarily for the Rhône during the First Pandemic (15) and for the Black Death (16). However, attempts to prove the preferential spread via rivers during Second Pandemic have recently been criticized (17, 18).
The site of Petting is geographically situated only 100 km southeast of Aschheim and Altenerding (SI Appendix, Fig. S9). However, it was located in the Roman province *Noricum ripense* whereas the sites with the distinct uniform lineage were situated in *Raetia secunda*

(Aschheim, Altenerding, Unterthürheim) or close by (Dittenheim). Although the administrative system of the Western Roman Empire had broken down by the mid-6th century, its political borders continued to be influential, not least because of the ecclesiastical system of dioceses that followed them. It is possible that these ancient boundaries influenced the spread of the two epidemic outbreaks in modern-day Bavaria. Since the river Inn separated *Raetia secunda* and *Noricum ripense*, this might suggest that rivers could serve as physical barriers to the spread of plague where river transport was negligible. This in turn would rather suggest the 'eastern route' or the 'southern route' for Petting as described above.
Complementing the previous results from Aschheim and Altenerding, our new data from Unterthürheim and Dittenheim underline the epidemic extent of this plague outbreak in early medieval Bavaria, totalling 16 individuals with genomic evidence for *Y. pestis* and an additional five PCR-positive individuals in Aschheim (19). Far from the urban centres of the time and any recorded outbreak of plague, the new molecular evidence stands in strong contrast to Durliat's claim that the Justinianic Plague was merely an urban phenomenon. Instead, we view this data as being in line with ancient statements by Procopius, John of Ephesos and Paul the Deacon who reported that the countryside of the Levant and Italy were severely impacted.

Radiocarbon Dating

At least one individual per burial was sampled for radiocarbon dating for all burials that tested positive for *Y. pestis*, assuming simultaneity of interment for the multiple burials. Samples were dated at the CEZ Archaeometry gGmbH, Mannheim, Germany. For Saint-Doulchard, published radiocarbon dates of adjacent burials in the same trench are reported (20). These as well as the raw radiocarbon dates of Aschheim and Altenerding (2, 3) were recalibrated for consistency. The raw radiocarbon dates were calibrated with IntCal13 (21) in OxCal v4.3.2 (22). All raw and calibrated dates are given in SI Appendix, Table S12; Fig. S8 shows the respective probability distributions. Some of the intervals completely pre-date the onset of Justinianic Plague (541) which could be explained by a marine or freshwater reservoir effect (23, 24) or human bone collagen offset (25). In the absence of C/N isotope data and a well-established method for addressing the human bone collagen offset, we report calibrated dates without any correction. A combination of the probability distributions using the function "Combine" in Oxcal was attempted for the trench inhumations of Lunel-Viel (LVC001, LVC002, LVC003, LVC005, LVC006, LVC007), the double burial 1175/1176 of Altenerding (AE1175, AE1176) and the quadruple burial 131–134 of Unterthürheim (UNT004, UNT005, UNT008). For Lunel-Viel, the resulting 2-sigma interval is 562–604 calAD (A_{comb} 148.6 %; A 109–127.9 %), for Altenerding 428–552 calAD (A_{comb} 107.1 %; A 104–105.9 %) and for Unterthürheim 428–544 calAD (A_{comb} 29.6 %; 14.5–100.9 %). The low agreement index for Unterthürheim is caused by the comparatively recent dating sample of UNT005 with an individual agreement index of 14.5 %. Therefore, the combination of uncorrected dates is deemed problematic in general and was dismissed for interpretation.

Cartography

All maps were generated in ArcGIS 10.4.1 (ESRI) using the 'World Ocean Basemap' without references. The sources for all historical occurrences are given in the SI Appendix. The mapped regions in Fig. 1 are primarily based on the Digital Atlas of Roman and Medieval Civilizations (DARMC; https://darmc.harvard.edu) maps "Provinces AD303–324" for Western Europe and "Provinces ca. AD500" for Eastern Europe, Middle East and Africa. The provinces in SI Appendix, Fig. S9 are based on a georeferenced map by Rettner and Steidl (26), the Roman roads are combined from Rettner and Steidl and the DARMC map "Roman Roads". The main rivers in Figs. 1C and 3 were taken from Natural Earth (ne_10m_river_lake_centerlines, http://www.naturalearthdata.com), based on data provided by the European Commission, Joint Research Centre, Institute for Environment and Sustainability (JRC IES).

Archaeological Context Information

The following site descriptions present condensed information on all sites examined in this study. The classification of multiple burial types follows McCormick (2015). Sex and age determination is based only on morphological examination. For the age classification, the German system (27) *is used as follows: Infans I (0–6 years old), infans II (7–12 years old), juvenile (13–20 years old), adult (20–40 years old), mature (40–60 years old), senile (more than 60 years old). Sites that were tested positive for plague are set in bold.*

Alladorf (ALL; Markt Turnau, Landkreis Kulmbach, Germany):
The Carolingian cemetery of Alladorf, dating roughly between 630 to 720, revealed 163 graves with remains of 276 individuals. However, the total size of the cemetery is unknown, since the excavation did not reach the borders of the burial area. B. Leinthaler classified three burials as double burials (type 1): 179/180 (ALL001, early adult female; ALL002, infans I), 184/185 (ALL003, infans II; ALL004, late adult to early mature male) and 203/204 (ALL005, late adult male; ALL006, infans I). The two burials 188/189 (infans I; late adult to early mature male) and 208/209 (infans I; early adult female) were classified as double burials with unclear simultaneity.
(28, 29)

Dirlewang (DIR; Landkreis Unterallgäu, Germany):
The Alemannic site of Dirlewang is a very small cemetery with 40 excavated burials and an expected number of 55 burials in total. It dates to the Late Merovingian period, from 650-700, based on the archaeological finds. Two double burials (type 1) were found on this site, 33/34 (DIR001, juvenile to early adult female; juvenile male) and 38/39 (adult male; DIR002, adult female). Graves 18 and 19 (mature male; early adult male) did not share the same grave pit but were buried very close to each other, indicating a connection. Burials 30/31, 36/37 and 2 were classified as non-simultaneous successive or additive double burials (type 2).
(30)

Dittenheim (DIT; Landkreis Weißenburg-Gunzenhausen, Germany):
The early medieval cemetery of Dittenheim recruited from a settlement on the site of the modern village. Only 6.5 km south of the *limes*, the settlement was probably well connected to the remaining Roman infrastructure. 2.5 km Southeast of the cemetery, remains of a Germanic fortification dating to the Migration Period were found, known as *Gelbe Bürg*. This structure went out of use around 500, just before the region fell under the rule of Franks.
The cemetery was known already by 1937. The first excavation campaign in 1968 revealed the first 114 burials. Later campaigns took place in 1971 (burials 115–164) and 1972 (165–244). The excavations probably reached the borders of the cemetery. However, some burials might have been lost due to erosion from a nearby river and from plowing. Besides the early medieval burials, older settlement traces from the Linear Pottery culture, Pre-Roman Iron Age and the imperial Roman period as well as one Bronze Age burial were found on the site.

The cemetery of Dittenheim, dating from the middle of the 6th century to the end of the 7th century, revealed 238 graves containing 244 individuals and 10 cremations from the same period. Other remarkable finds were three horse burials and one circular pit. Four burials were classified as double burials type 1: 8A/B (DIT007, mature female; mature male; possibly an additive burial), 18A/B (DIT003, mature male; DIT004, adult female), 22A/B (DIT005, infans I; DIT006, adult female) and 188A/B (DIT001; DIT002, both infans II). There were also three exceptional single burials in prone position (180, 201, 204) deviating from the contemporary burial rites.
(31)

Edix Hill (EDI; civil parish Barrington, Cambridgeshire, United Kingdom):

The Anglo-Saxon cemetery of Edix Hill, close to Barrington and Orwell was initially discovered in the 19th century. Excavations between 1989 and 1991 revealed part of an inhumation cemetery comprising 149 individuals in 115 graves, dating to between 500 and 625/650, although one burial was believed to be possibly Iron Age. It is estimated that there may originally have been around 300 burials in total with a complete cross-section of the population by age and sex suggesting that the burials relate to a community of 50–65 people spanning around 150 years. The initial dating of the cemetery was based primarily upon artefact typologies of the various grave goods and seriation by correspondence analysis, with burials broadly divided into earlier and later groups and some evidence for spatial patterning over time. Subsequent radiocarbon dating has broadly confirmed and refined the dating of the cemetery. Palaeopathological evidence suggested the presence of tuberculosis, leprosy and cancers. The human remains are currently held by Cambridgeshire County Council, who generously provided access to the material.
The cemetery contained a total of 18 multiple burials, of which 10 were classifiable as type 1: they comprise one quadruple burial with at least two of the individuals buried simultaneously and eight simultaneous double burials. Four double burials are of unclear simultaneity, another four and a triple burial are clearly understandable as type 2 (non-simultaneous). The site was initially not sampled for the purpose of plague screening, so samples from both multiple and single burials were screened. This includes the single graves 76 (Sk405, EDI001; juvenile), 69 (Sk359, EDI002, early adult female), 78 (Sk424, EDI003, juvenile), 46 (Sk146, EDI010, early mature male), 60 (Sk183, EDI011, adult female), 63 (Sk198, EDI012, early adult male), 83 (Sk436, EDI014, early adult female), 90 (Sk458, EDI017, senile female), 95 (Sk530, EDI018, late mature female), 97 (Sk551, EDI019, early adult male), 99 (Sk576, EDI020, mature male), 100 (Sk578, EDI021, early mature male), 105 (Sk592, EDI022, late mature female), the non-simultaneous double burial grave 66 (Sk322A, EDI013, at least adult male; SK322B, early adult female) and triple burial grave 18 (Sk42A1, infans I; Sk42A2, early adult with indet. sex); Sk42B, EDI009, early adult female), the simultaneous double burials grave 96 (Sk547A, adult female; Sk537B, EDI004, infans II), 106 (Sk626A, EDI005, early adult female; Sk626B, EDI006, early adult male), 9 (Sk13A, EDI007, mature to senile male; juvenile male) and 84 (Sk440A, EDI015, adult female; Sk440B, infans I) as well as the complex burial grave 2, with two individuals buried simultaneously (Sk3B, adult to mature male; Sk3C, EDI007, early adult

male) and two more individuals buried later (Sk3A1 and Sk3A2, both adult and possibly female and male respectively).
(32, 33)

Forchheim (FOR; Gemeinde Pförring, Landkreis Eichstätt, Germany):
Within the vestiges of a late medieval settlement, a quadruple burial was found without any context suggesting a larger burial ground. Two mature women (FOR002, individual 2; ind. 4), a mature man (FOR003, ind. 3) and a male juvenile (FOR001, ind. 1) were buried partially overlapping but all in East-West orientation. Remarkably, one of the women (2) was buried in the prone position. Grave goods such as a sax and a glass bead necklace date the burial to the second half of the 7th century. A belt buckle was indicative of a clothed inhumation.
(34)

Grafendobrach (GRA; Gemeinde Kulmbach, Landkreis Kulmbach, Germany):
The Carolingian cemetery of Grafendobrach revealed 85 burials; the excavations 1975–1976 did not reach the edges of the burial ground. The various garment artefacts found in the graves point to the late 9th to early 10th century. Besides several secondary burials (type 2), there were two remarkable graves: complex 83/84/85 consisted of two adjacent stone cists holding two women (83, GRA002, early mature; 85, GRA003 early adult) and an infant (84, ca. 1 year old) buried at the feet of 83. A single burial of a man (42, GRA001, early senile) was covered with stones, on top of which two neonates (43, 44) were found without individual burial pits.
(35)

Kleinlangheim (KLH, Gemeinde Großlangheim, Landkreis Kitzingen, Germany):
The Frankish cemetery of Kleinlangheim dates to the late 5th to early 8th century and was completely excavated from 1962 to 1969. It contained 244 burials and a remarkable number of 56 cremations; unlike other contemporary cemeteries, the graves showed signs of grouping, potentially indicating family groups. Nine multiple burials type 1 were identified, eight of them double burials: 35/36 (KLE005, adult male; late mature male), 41/42 (late adult male with skull fracture; juvenile to early adult), 147/148 (min. adult; neonate), 171/172 (two infans I), 208/209 (infans I; early adult female), 272/273 (late adult male; KLE004, senile male), 211 (KLE001, mature male; second individual cremated) and 218/219 (KLE002, mid-adult to mature male, KLE003, late adult to early mature female). The triple burial 100–102 contained remains of two infants (1–2 years old; 4–6 years old) and one mature individual of indeterminable sex.
(36)

Leobersdorf (LEO, Bezirk Baden, Austria):
The Avar cemetery of Leobersdorf was excavated between 1977 and 1983 and was dated to 640-800. In total, 154 burials containing remains of 171 individuals were excavated with an exceptionally high number of 25 multiple burials. 16 burials were identified as double burials, of which 9 contained each an adult and a subadult (16: adult female and infans I; 57: mature male and infans I; 74: adult female and infant; 93: mature male and infans I; 100: mature male

and juvenile female; 104: adult female and infans I; 114: adult male and infans I; 119: late mature male and infans II; 140: adult female and infans II). Four consisted of two adults (35: mature male and female; 86: adult female and mature male; 114: adult female and male; 144: senile and adult male) and two held only subadults (23: male juvenile and infans II; 103: two juvenile females; 145: infans I and II). The remains of infant individuals often were identified only after the morphological examination following the excavation.

Three burials were identified as triple burials (21: senile male, mature female and infant; 67: adult female and two infants; 82: juvenile male, LEO001; adult female, LEO002; infans I, LEO003). A fourth (99) was interpreted as a double burial (mature male and infant) with a secondary burial (female).

Two burials contained four individuals (105: mature female, juvenile female, infans I and neonates; 134: adult female, mature male, infans I and II). Burial 79 contained the remains of five individuals, identified as the burial of a mature female with secondary burials of four additional individuals (senile female and male, juvenile female and infans II) that had probably been buried on the same spot previously, and were reburied after the mature female.

(37, 38)

Lunel-Viel (LVH, LVC; Arrondissement Montpellier, Département Hérault, France):

The site of Lunel-Viel is equidistant (25 km) from the modern and Roman towns of Montpellier and Nîmes at the intersection of two paved roads that were active in late Antiquity: a later Roman secondary road running about 2 km southeast and parallel to the Roman *Via Domitia* that connected Iberia to Italy, and a road connecting the *Via Domitia* to the coastal lagoon *l'Étang d'or*, part of the complex of lagoons that included that of Lattes (anc. *Lattara*), famous already in Antiquity for its fishery (Pliny the Elder, Natural History, 9.29–32), and Maguelone, an island bishopric first mentioned in the 6th century when abundant emerging archaeological evidence documents far-flung Mediterranean shipping connections. A brief Roman-era occupation in the 2nd century BCE at Lunel-Viel was followed by continuous settlement beginning ca. 50–80 CE, although the ancient settlement area was abandoned in the 7th century, and the exact location of the new dwelling zones between the 7th and the 10th century remains unknown. Three inhumation cemeteries received the deceased of this community in succession from the 4th century. The earliest, at *Le Verdier,* functioned from the end of the 3rd century down to the beginning of the 6th century, and yielded 340 burials. The second, known as *Les Horts*, received burials of the late 5th to 7th centuries; 140 burials were excavated out of an estimated original total of ca. 200. The third is associated with the church of St. Vincent; burials began there ca. 520 and continued down to the 17th century; 97 have been excavated.

The cemetery *Les Horts* contained one clear case of a double burial, likely simultaneous (type 1), of two adult individuals (38A/B) placed head facing toes in a sarcophagus; the head and upper body of 38B were destroyed, along with the feet of 38A and part of the sarcophagus, during later leveling operations. Disarticulated remains of a third poorly preserved adult (38C, LVH001) were found pushed into the eastern end of the sarcophagus. Grave goods likely stemming from the original burial (one buckle, one plated buckle, one fibula, one pin and one clasp) date the burial of 38C to 475–550.

Outside the cemeteries, anomalous (deviant) burials of the remains of eight individuals were found during the excavation of the Gallo-Roman structures, called the *Quartier central*. Robbing of foundation stones in Antiquity had left empty trenches which were subsequently used for the summary interment of these individuals. A pin buckle, a spindle whorl and a knife found with the remains of the presumably clothed individuals date the interment to the 5th to 6th century. The positions and postures of the individuals clearly deviate from the burial customs seen in the contemporaneous cemeteries of *Les Horts* and *Le Verdier*: two adult women (3A: LVC003, adult; 3B: LVC004, mature) were placed under limestone slabs, with one resting her head on the thighs of the other. Westward of this group, a woman (1: LVC001, early adult) and another adult individual (2: LVC002, late adult to early mature, sex indeterminable) were found; the latter however had been disturbed by later agricultural activities. Whereas these individuals appear to have been laid rather carefully on the ground, the two men adjacent to the west (4: LVC005, mature, 5: LVC006, early adult) seem to have been carelessly dropped into the trench, the latter with his arms stretched over his head. Two more individuals, a young adolescent woman (6, LVC007) and an infant (7, sex indeterminable), were found in a second trench to the east. However, all individuals were interred more or less in supine East-West oriented position, as expected for early medieval Christian burials.
(39, 40)

München-Aubing (AUB, Stadt München, Germany):
The Baiuvarian cemetery of München-Aubing was excavated in two phases, 1938 and 1960–1963. It was used from the 5th to 7th century. A total of 896 graves was excavated. Four double burials (type 1) have been identified: 854/855 (AUB008, adult male; late mature male) 809/810 (AUB006; AUB007, two adult males), 724/725 (AUB004, adult male; AUB005, mature male; with possible later manipulation) and 676/677 (AUB002, mature female; AUB003, male individual). A third individual (675, infans I) was buried later on top of the double burial 676/677.
(41)

Neuburg an der Donau (NEU; Landkreis Neuburg-Schrobenhausen, Germany):
The site of Neuburg an der Donau was occupied at least from the first half of the 2nd century by a Roman military base associated with an urn cemetery with around 130 cremations. The burial ground *Seminargarten* is related to a later occupation period during the 4th century. From an estimated number of 150 burials, this site revealed 130 burials with 133 individuals. One burial was identified as a double burial (32A/B, adult female and juvenile with indeterminable sex), one as a triple burial (34A/B/C: mature male; NEU001, mature male; NEU002, adult male). Both are part of zone 1, dating to 330–360. Two additional burials (14, 28) were suggested to contain “mother and child” (neonate/infant).
(42, 43)

Peigen (PEI; Markt Pilsting, Landkreis Dingolfing-Landau, Germany):

The early medieval cemetery of Peigen dating to the mid-5th to the 7th century revealed around 274 burials. Among these, three could be identified as double burials. Double burial 18 contained an adult woman (18-1, PEI001) and an early adult woman (18-2, PEI002), buried in opposite orientation. Double burial 63 contained an adult woman (63-1, PEI004) and a senile man (63-2, PEI005). The burial of a late mature male (109-1) and a child (infans I, 109-2) form the third clear double burial. The burials 62-1 of a juvenile male and 62-2 of a late mature female (PEI003) were adjacent but at different depth and therefore of questionable simultaneity (type 2).
(44)

Petting (PET; Landkreis Traunstein, Germany):

Although located in Germany today, Petting falls in a region that was in ancient times associated to the Roman city and later ecclesiastical center of *Iuvavum* (today Salzburg, Austria) and has therefore a different settlement history than other parts of southern Bavaria. The cemetery of Petting was discovered in 1991 and excavated within the following two years. A comprehensive publication of the site of Petting is still pending, not least because the archaeological findings were not available for research until the ownership situation was clarified in 2010. Studies so far concentrated on individual burials or specific artefacts.
The cemetery was completely excavated, counting 721 burials. Only 24 burials were lost due to destruction prior to the excavation: around 50 % of the burials fell victim to ancient grave robbers. At least four multiple burials were observed in the cemetery, all presumably type 1. The double burial 172/173 contained a female and a male individual; the double burial 377/378 a late adult to mature and a late adult to early mature male (PET003/PET004), the triple burial 342/343/344 at least one early adult male (PET001) and an infans II (PET002). The double burial 630/631 of an early adult female (PET006) and an early mature female (PET007) was associated with a single burial of an infans II (PET005) on top.
(45, 46)

Regensburg Fritz-Fend-Straße (RFF; Stadt Regensburg, Germany):

Regensburg, *Castra Regina,* was an important Roman military base with a civilian settlement of the province *Raetia*. The extensive adjacent cemetery *Kumpfmühl* was excavated in several campaigns following the first excavation in 1872 by J. Dahlem. He documented 827 burials and cremations but left the vast majority of features undocumented, which led S. v. Schnurbein to the assumption that the first excavation uncovered around 3000 cremations and 2000 burials, mainly dating to the late 3rd to mid 4th century. More features were lost in this area due to later construction works without archaeological survey. An excavation in 1999 revealed 100 additional cremations and 50 burials. The excavations of *Fritz-Fend-Straße* and *Im Güterbahnhof* took place in 2011 and revealed 48 cremations/115 burials and 161 cremations/116 burials, respectively. The site *Fritz-Fend-Straße* contained two exceptional double burials of type 1: In burial 30, the second individual (30-1: RFF001, late adult to early mature male) was laid in prone position and opposite orientation on top of the first (30-2), who

was buried in supine position. In 53, the two individuals, a juvenile female (53-2: RFF002) and an early mature male (53-3, RFF003), were buried in crouched position facing in the same direction but in opposite orientation. An archaeological or radiocarbon date for *Fritz-Fend-Straße* has not yet been published, but a similar date as for *Kumpfmühl* can be assumed. (47)

Saint-Doulchard Le Pressoir (LSD; Arondissement Bourges, Département Cher, France):

In the former village of Saint-Doulchard, 2 km from Bourges, an archaeological survey in 2007 revealed a dense funerary space, dated from the 7th to 12th centuries. The town is first mentioned as *Sanctus Dulcardus* in the 7th century, named after the hermit Dulcardus (late 6th century). It is likely that the cemetery was established on the site of Dulcardus' hermitage. However, a tumulus (6th to 4th c. BCE) and a Roman villa found in the vicinity of the excavated funerary space attest earlier occupation of the site.

In 2009, the rescue excavation led by P. Maçon excavated a part of this cemetery measuring approximately 400 m², reaching one of its boundaries in the form of a ditch that borders the burial area and runs from the northeast to the southwest. This ditch was also used for burials: 57 individuals in 48 burials have been discovered within it whose organization and funerary practice may indicate a particular mortality crisis. However, some secondary burials and stratigraphically overlapping graves within the ditch could suggest that it was used on multiple occasions. Eleven multiple burials were found, including nine double burials F206-35/36 (mature; fetus), F206-68/69 (LSD008, mature to senile male; infans I), F206-71/72 (LSD009, senile male, infans I), F206-79a/79b (LSD011, mature to senile male; infans I), F206-93/145 (LSD012, early adult; early adult male), F206-152a/152b (infans I, infans II), F206-155a/155b (senile female; LSD019, mature to senile male), F206-156a/156b (LSD020, mature to senile male; infans I) and F206-204a/204b (LSD023, mature male; infans I-II), and two triple burials F206-91a/91b/91c (mature male, infans I, infans I), F 206-172a/172b/172c (infans I; LSD022, juvenile; LSD021, mature female). Three more double burials were found within the cemetery area delimited by the ditch: F206-75a/-75b (infans I to juvenile; late adult to mature female), F206-229a/-229b (late adult to mature female, infans I) and F206-235 (at least adult male; subadult).

In addition to several multiple burials, a selection of single burials within the ditch were also sampled for this study: F206-31 (LSD001, early adult female), F206-38 (LSD002, mature female), F206-40 (LSD003, mature male), F206-45 (LSD004, mature to senile male), F206-51 (LSD005, mature male), F206-52 (LSD006, early adult), F206-65 (LSD007, mature to senile), F206-78 (LSD010, mature to senile male), F206-132 (LSD013, mature male), F206-136 (LSD014, mature female), F206-144 (LSD015, juvenile), F206-151 (LSD016, mature male), F206-153 (LSD017, infans II), F206-154 (LSD018, early adult), F206-208 (LSD024, juvenile), F206-215 (LSD025, mature to senile female), F206-216 (LSD026, infans II).

A palaeodemographic analysis highlighted similarities in the structure of the population buried in the ditch with mortality profiles frequently observed in epidemic contexts, including plague. This suspicion is reinforced by the taphonomic evidence arguing for simultaneous interments

(type 1) in an easily available structure, separated from the rest of the community. Nevertheless, the presence of multiple burials in the space enclosed by the ditch might indicate a gradual shift in funerary practice at the beginning of an epidemic when mortality was still limited.
Radiocarbon dating was performed on five individuals buried in the ditch. The calibrated 2-sigma intervals cluster in two groups (ca. 650–880 and ca. 720–920), which might indicate the usage of the ditch as funerary space for multiple events. A combination of all five dates is rejected by Oxcal (df=4, T=11.2 [5% 9.5]), further substantiating the hypothesis of multiple events.
(20)

Sindelsdorf (SIN; Landkreis Weilheim-Schongau, Germany):
The early medieval cemetery of Sindelsdorf consists of 331 burials with remains of 354 individuals dating from 500 to 720. It included three double burials type 1 (25/26 senile male and mature male; 51/52, senile and mature male (SIN006, SIN007); 154/155, infant and senile female) and a burial group of three individuals with one buried later on top (163: infant; 164: SIN002, late adult to early mature male; 165: SIN003, adult female; 162: SIN001, senile male). The double burial 25/26 with a later burial on top (24, mature female) may be identified as a probable homicide due to a perimortem skull fracture (26).
(48)

Straubing Azlburg I/II (SAZ; Stadt Straubing, Germany):
Straubing, called *Sorviodurum* by the Romans, was an important *castrum* on the Danubian border of *Raetia*. The Late Roman cemeteries – around 200 m apart from each other – were excavated in 1981 (*Azlburg I*) and 1984 (*Azlburg II*). However, the borders of the cemeteries were not reached on all sides, so the original dimensions remain unclear. They were in use at roughly the same time, approximately between 300 and 450. *Azlburg I* contained 107 graves with 111 individuals and one cremation. The burial 54-1/-2/-3 was identified as a triple burial (SAZ002, male juvenile; SAZ003, male juvenile; third individual min. adult). The double burial 16/17 contained the remains of two children (SAZ001, infans I; neonate). *Azlburg II* contained additional 434 graves with 45 individuals. The double burial 5a/b of two adult men was interpreted as the burial of two soldiers.
(49)

Unterthürheim (UNT; Gemeinde Buttenwiesen, Landkreis Dillingen an der Donau, Germany):
The town of Unterthürheim is probably the site of the early medieval settlement that used the burial ground *Buttenwiesen*. Traces of an older Imperial Roman settlement (1st to 5th century) were found on the *Thürlesberg* hill, around two kilometers from the cemetery; it was near the Roman roads *Via Iuxta Danuvium* (following the Danube) and the *Via Claudia Augusta* (connecting *Augusta Vindelicum*/Augsburg with Italy) which intersect at the fort *Submuntorium*, around 10 km Northeast of Unterthürheim.

The Alemannic cemetery has been known since 1889, when a local resident discovered the first six graves. Additional burials were found between 1943 and 1966. The first professional excavation took place in 1968 (graves 1–42), later excavations occurred between 1969 and 1972 (graves 43–178) and in 1979 (graves 180–238). All of them were rescue excavations, which explains the scattered trenches and limited dimensions: The excavations are thought to have reached the southern and northern border of the cemetery, but the extent to the west and east remain unknown. In total, 256 burials were excavated of which 230 were preserved well enough for more detailed examination. They are archaeologically dated to 525 to 680.
H. Lüdemann lists in total 14 double burials, two triple burials and one quadruple burial. The triple burials 120/122 and 124/125 as well as the double burials 167/168, 186/186a and 217/217a were classified as secondary burials (type 2). Burials 12 (mature with indeterminable sex, subadult) 63/64 (UNT006, adult female; UNT007, infans I), 79/80 (adult female, infans I), 116 (mature female; UNT001, infans II), 140/141 (mature male, early adult female), 145/146 (adult to early mature female, male) and 189 (adult females, neonates) presumably simultaneous double burials (type 1). 65/66 (infans I, infans II) and 190/190a (adult to early mature female, early adult male) were classified by C. Grünewald as possible secondary burials (type 2). For the burials 97/98 and 147/149, the classification is unclear.
The quadruple burial 131–134 (UNT004, infans I; UNT005, adult male; infans I; min. adult male) was associated with two single burials: 129 (UNT002, infans I) and 130 (UNT003, mature male).
(38, 50)

Valencia Plaça de l'Almoina (VAL; Ciudad de Valencia, Spain):

The site of Valencia, *Plaça de l'Almoina* is an intramural cemetery that was founded in the 5th century and remained in use during the Visigothic period in the 6th and 7th centuries. It was excavated from 1985 to 1999. Contrary to the earlier Roman practice, the necropolis was not located outside the city walls but close to a shrine commemorating the martyrdom of St. Vincent, and adjacent to the cathedral. Many burials still follow the later Roman tradition that made use of *tegulae* and amphoras. Among the later burials, there are several monumentalizing collective tombs that were probably used as elite family tombs for successive burials.
The excavated areas of the necropolis contained a number of multiple burials of differing types and dates, including 15 or 16 collective slab tombs that appear to reflect successive burials (type 2: details in McCormick 2016, 1024n15). At the moment of sampling, the human remains were comingled and only sorted by find numbers (corresponding the numbers VAL001–009), so the assignment to individuals is not possible for this site. Four or five features seemed to be of type 1, i.e., reflecting at least in part simultaneous burials. The quadruple burial tomb 4 was covered with *tegulae* and contained four individuals who were piled one on top of another in an east-west orientation (two samples were taken, VAL008). Tomb 28 was stone-lined and just north of an apse identified as a memorial shrine to the martyrdom of St. Vincent; it contained a minimum of 21 individuals, from whom sixteen samples were taken (VAL005, VAL006, VAL007). Tomb 50, just southwest of Tomb 28 (and actually under the wall of that apse), may well have been a continuation of Tomb 28; it contained at least seven individuals, from whom

seven samples were taken (VAL003, VAL004). Tombs 28 and 50 are lower than and earlier than the wall of the apse. The apse itself seems well dated to the late sixth or early seventh century, based on pottery excavated in a pit under the apse pavement. Multiple tomb 40 was a pit burial containing four supine individuals (five samples, VAL002) oriented east-west in the entry from the east into the apse of the memorial shrine. Tomb 41 is south of the apse structure which might indicate a privileged burial space. It is a pit that seems to have used pre-existing Roman walls to the south and east for two sides; bricks were simply placed without mortar to form the other two sides of the burial space, which might suggest a hasty or improvised burial. It contained the very disturbed remains of at least 15 individuals, including 4 subadults, from which we took seven samples (VAL001).
(38, 51–53)

Waging (WAG; Landkreis Traunstein, Germany):

The early medieval cemetery of Waging, close to the site of Petting, was discovered and excavated in 1987/1988. The excavators deduced a discontinuity between the previous Roman settlement (until 300) and the early medieval colonization related to the burial ground that was in use between 530 and 700. Similarly to the site of Petting, the resettlement was most likely dominated by *Iuvavum* (modern Salzburg, Austria) in the southeast.
Although the 239 burials were subsequently analyzed and the artefacts went into a local exhibition after extensive conservation measures, the site is still not published in a comprehensive way. At least three burials were identified as multiple burials: the double burial 200/201 of an early mature male and infans II (WAG002; WAG003) were located on top of the burial of a potentially male infans II (WAG001). The double burial 37 contained remains of a potentially female infans II (WAG004) and a juvenile to early adult individual (WAG005). Burial 39 was identified as a double burial of a potentially female infans II (WAG006) and a late mature female (WAG007).
(45, 54)

Westheim (WES; Landkreis Weißenburg-Gunzenhausen, Germany):

The Merovingian cemetery of *Westheim-Mehlbuck*, excavated between 1979 and 1985, revealed remains of 255 individuals in 228 graves and dates to the 6th to mid-7th century. Five burials were clearly identified as double burials: 17a/b, 26a/b, 36a/b, 52a/b, 106a/b and 128a/b. Six additional burials were interpreted as presumably double burials of a child with a parent: 172, 190, 202, 206, 208 and 210. The triple burial I, uncovered during a previous excavation in the 1910s, held the remains of a late adult to early mature male (1-1, WES001), a late mature female (1-3, WES003) and of a third individual (1-2, WES002, min. adult, probably female). Burial 13a/b was reported by H. Lüdemann as a quadruple. However, according to R. Reiss, the burial contained only disturbed remains of two males (adult, mature).
(38, 55)

Sources for mapping plague outbreaks between 541 and 750 CE

Clysma as possible starting point

Tsiamis et al. (2009); Harper (2017) 215–218

541: Pelusium

Prokopios, *BP* 2.22, 6

See also: Stathakopoulos (2004) no. 102

541: Gaza, Ashkelon, Negev

John of Ephesos, *Fragment E* 77

Epigraphic material in Conrad (1996) 95

See also: Stathakopoulos (2004) no. 103

541: Alexandria

Prokopios, *BP* 2.22, 6

John of Ephesos, *Fragment E* 77, 80

Chron. Seert 185

Michael the Syrian 2 (235–238)

See also: Stathakopoulos (2004) no. 104

542: Jerusalem and countryside

Cyril of Scythopolis, *Vita* of Kyriakos 10

See also: Stathakopoulos (2004) no. 105

542: Izra

Epigraphic material in Koder (1995) 13–18

See also: Stathakopoulos (2004) no. 106

542: Antioch

Vita of Symeon Stylites Iunior 69

See also: Stathakopoulos (2004) no. 107

542: Apamea

Evagrios, *Hist. eccl.* 4.29

See also: Stathakopoulos (2004) no. 108

542: Emesa

Zacharias Rhetor, *Fragment ch. IX*

Leontios of Neapolis, *Vita* of Symeon Salos 151

See also: Stathakopoulos (2004) no. 109

542: Myra

Vita of Nicholas of Sion 52

See also: Stathakopoulos (2004) no. 110

542: Constantinople

Prokopios, *BP,* 2.22, 2.23

John of Ephesos, *Fragment E* 74–93

John Malalas, 482

Theophanes, *Chron.* AM 6034

See also: Stathakopoulos (2004) no. 111

542: Sykeon

Theodore of Sykeon, Vita 8 (I 7–8)

See also: Stathakopoulos (2004) no. 112

542: North Africa

Victor of Tunnuna, *Chronica* ad a. 542 (201)

See also: Stathakopoulos (2004) no. 114

542: Sicily

Byzantina Siciliae, 133

See also: Stathakopoulos (2004) no. 115

543: Sufetula

Epigraphic material from Sufetula nos. 1–4 (277–280)

See also: Stathakopoulos (2004) no. 117

543: Italy, Illyricum

Marcellinus Comes ad a. 543 (107)

See also: Stathakopoulos (2004) no. 116

543: Gaul, Arles, Reims, Trier

Gregory of Tours, *Lib. hist.* 4.5, 6.15, 6.33

Gregory of Tours, *Lib. vitae patrum* 6.6, 17.4

Gregory of Tours, *Liber in gloria confessorum* 78

543: Spain

Victoris Tunnunensis Chronicon, *Consularia Caesaraugustana*, ad a. p.c. Basili II

See also: Kulikowski (2007) 150–151

543–544: Rome

Inscriptiones Christianae Urbis Romae 1.1452, 2.4287, 7.17624, 2.5088, 2.4289, 8.20839, 2.5087, 2.5087, 2.5087.

See also: Stathakopoulos (2004) no. 118

544: Ireland, Britain

Annals of Tigernach 137, 198

Adomnán, *Vita Columbae* 348

See also: E. Phillimore (1888); A. Dooley (2007), 216; J. Maddicott (2007), 173–174

558: Constantinople

Agathias, *His.,* 5.10

John Malalas, *Chron.* 18.127 (489)

Theophanes, *Chron.* AM 6050

Agapios, *Kitab al-'Unwan*

See also: Stathakopoulos (2004) no. 134, Harper (2017) no. 1

561–562: Cilicia and Anazarbos, Syria, Mesopotamia, Antioch

Theophanes, *Chron.* AM 6053

Vita Symeon Stylites Iunior 126–129

Chron. ad a. 640

Barhadbšabba 388–389

Chron. Seert 185–186

Amr ibn Matta 42–43
See also: Stathakopoulos (2004) no. 136, Harper (2017) no. 2

565: Liguria, Northern Italy
Paul the Deacon, *Hist. Langobardorum* 2.4
See also: Stathakopoulos (2004) no. 139, Harper (2017) no. 3

571: Italy, Gaul, Bourges, Chalon-sur-Saône, Clermont, Dijon, Lyon
Marius of Avenches, a. 571
Gregory of Tours, *Lib. hist.* 4.31–32
See also: Stathakopoulos (2004) no. 144, Harper (2017) no. 4

573–574: Constantinople, Egypt, Syria and Antioch
John of Biclaro, a. 573
Agapios, *Kitab al-'Unwan*
John of Nikiu 94.18
Chron. ad a. 846
Michael the Syrian 10.8 (346)
See also: Stathakopoulos (2004) no. 145, Harper (2017) no. 5

576: Ireland
For secondary sources see:
Dooley (2007), 219
Woods (2003)

582–584: Southwestern Gaul, Narbonne, Spain
Gregory of Tours, *Lib. hist. 6*.14, 6.33
See also: Harper (2017) no. 6

586: Constantinople
Agapios, *Kitab al-'Unwan*
See also: Harper (2017) no. 7

588: Gaul, Lyon, Marseille, Spain
Gregory of Tours, *Lib. hist.* 9.21–22
See also: Harper (2017) no. 8

590–591: Rome, Narni, Ravenna, Istria and Grado, Rhône Valley, Avignon, Viviers
Gregory of Tours, *Lib. hist.* 10.1, 10.23
Gregory the Great, *Dial.* 4.18, 4.26, 4.37; *Ep*. 2.2
Paul the Deacon, *Hist. Langobardorum,* 3.24, 4.4
Liber pontificalis 65
See also: Stathakopoulos (2004) nos. 151, 154; Harper (2017) nos. 9, 10

592: Syria, Palestine, Antioch
Evagrius, *Hist. eccl.* 4.29
I. Palaestina Tertia nos. 68–70
Hassan ibn Thabit in Conrad (1981) 154
See also: Stathakopoulos (2004) no. 155, Harper (2017) no. 11

597: Thessalonica and countryside
Mir. Demetr., 3 & 14

See also: Stathakopoulos (2004) no. 156, Harper (2017) no. 12

598: Thrace

Theophylact Simocatta, *Hist.* 7.15.2

See also: Stathakopoulos (2004) no. 159, Harper (2017) no. 13

599–600: Constantinople, Asia Minor and Bithynia, Syria, North Africa, Italy, Marseille

Michael the Syrian, 10.23 (387)

Chron. ad a. 1234

Gregory the Great, *Ep.* 9.232, 10.20

Paul the Deacon, *Hist. Langobardorum,* 4.14

Elias of Nisibis, a. 911

Thomas of Margâ, *Book of Governors,* 11

Fredegar, *Chron.* 4.18

See also: Stathakopoulos (2004) no. 160, Harper (2017) no. 14, Biraben and LeGoff (1975) 75

609: Cortijo de Chinales, Spain

CIL II 7.677

See also: Harper (2017) no. 15, McCormick (2016) 327

619: Constantinople, Alexandria

Mirac. sanct. Artemii, 34

See also: Stathakopoulos (2004) no. 173, Harper (2017) no. 17

626–628: Palestine, Mesopotamia

Michael the Syrian, 11.3 (409)

Eutychius, *Annales*

al-Tabari, 1061

Arabic sources in Conrad (1981) 159–163

See also: Stathakopoulos (2004) nos. 177, 178; Harper (2017) no. 18

638–639: Palestine, Syria, Mesopotamia

Michael the Syrian, 11.8 (423)

Elias of Nisibis, (AH 18)

Chron. ad a. 1234, 76 (AH 18)

Arabic sources in Conrad (1981) 167ff.

See also: Stathakopoulos (2004) no. 180, Harper (2017) no. 20

663–666: England, Ireland

Adomnán, *Vita Columbae* 47

Bede, *Hist. eccl.* 3.23, 27, 30; 4.1, 7, 8

Bede, *Vit. Cuthb.* 8

See also: Harper (2017) no. 21, Maddicott (2007)

670–671: Kufa

Arabic sources in Conrad (1981) 250–253

See also Stathakopoulos (2004) no. 185, Harper (2017) no. 22

672–673: Egypt, Palestine, Mesopotamia, Kufa

Theophanes, *Chron.* AM 6164

Agapios, *Kitab al-ʻUnwan*

Arabic sources in Conrad (1981) 253ff.

See also: Stathakopoulos (2004) nos. 185, 186; Harper (2017) no. 23

680: Rome, Pavia

Paul the Deacon, *Hist. Langobardorum* 6.5

Liber pontificalis 81

See also Stathakopoulos (2004) no. 192, Harper (2017) no. 24

684–687: England, Ireland

Adomnán, *Vita Columbae* 47

Bede, *Hist. eccl.* 4.14

See also Harper (2017) no. 25, Maddicott (2007)

687–689: Syria, Mesopotamia, Basrah

John bar Penkaye, *Riš Mellē* XV 160–165 (68–71)

Arabic sources in Conrad (1981) 263ff.

See also: Stathakopoulos (2004) nos. 194, 195; Harper (2017) no. 26

689–690: Egypt

Arabic sources in Conrad (1981) 271ff.

See also: Stathakopoulos (2004) no. 196, Harper (2017) no. 27

693: Spain, southwestern Gaul

Mozarabic Chronicle of 754, 41

See also: Harper (2017) no. 28, Kulikowski (2007) 153–154

698–700: Constantinople, Syria, Mesopotamia

Elias of Nisibis (AH 79 and 80)

Chron. ad a. 819, AG 1011

Theophanes, *Chron.* AM 6190 & 6192

Nikephoros, *Brev.* 41

Leo Grammaticus, *Chron.* 167

Arabic sources in Conrad (1981) 274ff.

See also: Stathakopoulos (2004) nos. 198–200; Harper (2017) no. 29

704–706: Syria, Mesopotamia, Basrah, Kufa

Michael the Syrian, 11.17 (449)

Chron. Zuqnin a. 1016

Arabic sources in Conrad (1981) 278ff.

See also: Stathakopoulos (2004) nos. 201, 203; Harper (2017) no. 30

707–709: Spain

Akhbar majmu'a, 7.BkS

See also Harper (2017) no. 31, Kulikowski (2007)

713: Syria

Chronicle of Disasters a. 1024

Michael the Syrian, 11.17 (452)

Chron. ad a. 819 & ad a. 846 a. 1024

See also Stathakopoulos (2004) no. 205, Harper (2017) no. 32

714–715: Egypt

Severos, *History of the Patriarchs* 17

See also Stathakopoulos (2004) no. 207, Harper (2017) no. 33

718–719: Syria, Mesopotamia, Basrah

Arabic sources in Conrad (1981) 286ff.

See also: Stathakopoulos (2004) no. 209, Harper (2017) no. 34

725–726: Syria, Mesopotamia

Theophanes, *Chron.* AM 6218

Vita Willibaldi 4

Michael the Syrian, 11.19 (436)

Agapios, Kitab al-ʻUnwan

Elias of Nisibis (AD 107)

Chron. ad a. 819 a. 1036

See also Stathakopoulos (2004) no. 213, Harper (2017) no. 35

729: Syria

Michael the Syrian, 11.21 (463)

Harper (2017) no. 36

732–735: Egypt, Palestine, Syria, Mesopotamia

Theophanes, *Chron.* AM 6225

Agapios, *Kitab al-ʻUnwan*

Arabic sources in Conrad (1981) 291ff.

See also Stathakopoulos (2004) no. 214, Harper (2017) no. 37

743–750: Egypt, North Africa, Syria, Mesopotamia, Basrah, Sicily, Italy, Greece, Constantinople, Armenia

Severos, *History of the Patriarchs* 18

Michael the Syrian, 11.22 (465– 66)

Chron. Zuqnin a. 1055–1056, a. 1061– 62

Chron. ad a. 1234

Theophanes, *Chron.* AM 6238

Nikephoros, *Brev.* 67

Nikephoros, *Antirhetikos III* 496B

Theodore Studites 805B–D

Michael Glycas, *Annales* 527

John Zonaras, *Epit. hist.* 15.6

John of Naples, *Gesta episcoporum neapolitanorum* 42

Arabic sources in Conrad (1981) 293ff.

See also: Stathakopoulos (2004) nos. 218–222, Harper (2017) no. 38, McCormick (2007) 292

Primary sources:
Adomnán, *Vita Columbae* = *Adomnán's Life of St. Columba.* Ed. and tr. A.O. Anderson and M. O. Anderson (Oxford 1991)
Akhbar majmu'a = *A History of Early Al-Andalus: The Akhbar Majmu'a.* Tr. D. James (New York 2012)
al-Tabari = *The Sāsānids, the Byzantines, the Lakhmids, and Yemen.* Tr. C. E. Bosworth (New York 1999).
Amr ibn Matta = *Maris, Amri et Slibae de Patriarchis Netsorianorum commentaria.* Ed. H Gismondi (Rome 1896).
Annals of Tigernach = *The Annals of Tigernach*. Ed. W. Stokes. *Revue Celtique* 16–17 (1895–1896). Reprint, 2 vols. (Dyfed 1993).
Agapios, *Kitab al-'Unvan* = *Kitab al-'Unvan.* Ed. A. Vasiliev. In *PO* 5.4, 7.4; 8.3; 11.1 (1910, 1911, 1912, 1915).
Agathias = *Agathiae Myrinei Historiarum Libri Quinque.* Ed. R. Keydell. *CFHB* 2 (Berlin 1967).
Barhadbšabba = *Cause de la foundation des écoles.* Ed. A. Scher. In *PO 4*, 388–389.
Bede, *Hist. eccl.* = *Bede's Ecclesiastical History of the English People.* Eds. B. Colgrave, R. A. B. Mynors (Oxford 1991).
Bede, *Vit. Cuthb.* = *Two Lives of St. Cuthbert*. Ed. B. Colgrave (Cambridge 1940).
Chron. ad a. 640 = *The Seventh Century in the West-Syrian Chronicles*. Tr. A. Palmer. *Translated texts for historians 15* (Liverpool 1993).
Chron. ad a. 846 = *Chronicon ad a. 846*. Ed. I. B. Chabot, E. W. Brooks. CSCO 4, Syr. 4 (Louvain 1907)
Chron. ad a. 819 = *Anonymi auctoris Chronicon ad annum Christi 1234 pertinens, I. praemissum est Chronicon Anonymum ad A. D. 819 pertinens.* Ed. J.-B. Chabot. In CSCO 81; Syr. 36 (Paris 1916).
Chron. ad a. 1234 = *Anonymi auctoris Chronicon ad annum Christi 1234 pertinens, I. praemissum est Chronicon Anonymum ad A. D. 819 pertinens.* Ed. J.-B. Chabot. In CSCO 81; Syr. 36 (Paris 1916).
Chronicle of Disasters = *A Chronicle of Disasters dated AD 716.* In: *The Seventh Century in the West-Syrian Chronicles*. Tr. A. Palmer. *Translated texts for historians 15,* (Liverpool 1993)
Chron. Seert = *Chronicle of Seert (Histoire Nestorienne).* Ed. A. Scher. In *PO* 4, 213–313; 5, 217–344; 7, 93–203.
Chron. Zuqnin = *Chronicon anonymum pseudo-dionysianum vulgo dictum.* Ed. J.-B. Chabot. CSCO 91, 104; Syr. 43, 53 (Paris 1927, 1933)
Victoris Tunnunensis Chronicon = *Victoris Tunnunensis Chronicon cum reliquiis ex consularibus Caesaraugustanis et Iohannis Biclarensis Chronicon*. Ed. C. Cardelle de Hartmann. Corpus Christianorum, Series Latina 173A (Turnhout 2001).
Cyril of Scythopolis = *Kyrillos von Skytopolis.* Ed. E. Schwartz. TU 49.2 (Leipzig 1939).
Elias of Nisibis = *Opus Chronologicum.* Ed. E. W. Brooks. CSCO 63, Syr. 23 (Louvain 1964).
Eutychius, *Annales* = *Annales.* Ed. L. Cheikho, CSCO 50–51 (Paris 1904).

Evagrius *Hist. eccl.* = *Historia ecclesiastica.* Eds. J. Bidez, L. Parmentier. *The Ecclesiastical History of Evagrius* (London 1898).
Fredegar, *Chron.* = Fredegar, *Chronica*. Ed. B. Krusch, SRM, 2.128.5–6 (Hannover 1888).
Gregory of Tours, *Lib. hist.* = *Libri historiarum X.* Eds. B. Krusch, W. Levison. MGH SRM, 1.1 (Hannover 1937–1951).
Gregory of Tours, *Liber in Gloria confessorum* = *Liber in Gloria confessorum*. B. Krusch, MGH SRM, 1.2 (Hannover 1885).
Gregory of Tours, *Lib. vitae partum* = *Liber vitae partum..*Ed. B. Krusch, MGH SRM, 1.2 (Hannover 1885).
Gregory the Great, *Dial.* = *Dialogorum libri iv.* Ed. A: de Vogüé. SC 251, 260, 265 (Paris 1978–1980).
Gregory the Great, *Ep.* = *Registrum epistularum.* Ed. D. Norberg. CC 140–40A (Turnhout 1982).
John bar Penkaye, *Riš Mellē* = *North Mesopotamia in the Late Seventh Century: Book XV of John bar Penkaye's Riš Mellē.* S. Brock. Jerusalem Studies in Arabic and Islam 9 (Jerusalem 1989).
John of Biclaro = *Chronica.* Ed. T. Mommsen. MGH AA 9, 2, 213–214 (Berlin 1894).
John of Ephesos *Fragment E* = *Historiae Ecclesiasticae fragmenta.* Eds. W. J. van Douwen, J. P. N. Land. *Verhandelingen der koniglijke Akademie van Wetenschapen Afdeeling Letterkunde* 18, 197–264 (Amsterdam 1899).
John Malalas = *Ioannis Malalae chronographia*. Ed. I. Thurn (Berlin 2000).
John of Naples, *Gesta episcoporum neapolitanorum* = *Johannes Diaconus, Gesta episcoporum neapolitanorum*. Ed. G. Waitz. MGH SRL, 425.15–19 (Hannover 1878).
John of Nikiu = *The Chronicle of John, Bishop of Nikiu.* Ed. R. H. Charles (London 1916).
John Zonaras, *Epit. hist.* = *Ioannis Zonarae epitome historiarum*. Ed. L. Dindorf (Leipzig 1870).
Leo Grammaticus, *Chron.* = *Leonis Grammatici Chronographia*. Ed. I. Bekker. CSHB 42 (Bonn 1842).
Leontios of Neapolis, *Vita of Symeon Salos* = *Das Leben des heiligen Narren Symeon*. Ed. L. Rydén. *Acta Universitatis Upsalensis. Studia Graeca Upsaliensia* 4, 151 (Stockholm, Göteborg, Upsala 1963).
Liber pontificalis = *Liber pontificalis.* Ed. T. Mommsen. MGH Gesta Pont. Rom. 1 (Hannover 1898).
Marius of Avenches = *Chronica.* Ed. T. Mommsen. MGH AA 9, 2, 225–240 (Berlin, 1894).
Marcellinus Comes = *Chronicon ad annum DXVIII.* Ed. T. Mommsen, MGH AA 11, 60–108 (Hannover 1894).
Michael Glycas, *Annales* = *Michaelis Glycae Annales*. Ed. I. Bekker. CSHB 24 (Bonn 1836).
Michael the Syrian = *Chronique de Michel le Syrien, patriarche Jacobite d'Antioche (1166–1199).* Ed. J.-B. Chabot (Paris 1899–1924).
Mir. Demetr. = *Les plus anciens recueils de Miracles de Saint Démétrius et la pénétration des Slaves dans les Balkans.* P. Lemerle. 2 vols. (Paris 1979–1981).

Mirac. sanct. Artemii = *Miracula Sancti Artemii.* Ed. A. Papadopoulos-Kerameus. *Varia Graeca Sacra*. 34, 52 (Saint Petersburg 1909).

Mozarabic Chronicle of 754 = Cronica Mozarabe de 754: Edicion critica y traduccion. Ed. and tr. J. E. Lopez Pereira (Zaragoza, 1989).

Nikephoros, *Antirhetikos III = Nikephoros Patriarches*, *Antirhetikos* III. In PG 100.

Nikephoros, *Brev. = Nikephoros, Patriarch of Constantinople: Short History.* Ed. C. Mango. CFHB 13 (Washington 1990).

Paul the Deacon = *Historia Langobardorum*. Ed. G. Waitz. SS rer. Langobard. (Hannover 1878).

Prokopios *BP = Persian Wars*. Eds. J. Haury, G. Wirth, *Opera*. Vol. 1 (Leipzig 1962–1964).

Severos, *History of the Patriarchs = History of the Patriarchs of the Coptic Church of Alexandria.* Ed. B. Evetts, PO 1.2, 5.1, 10.5 (Paris 1906–1915).

Theodore of Sykeon, *Vita = La vie de Théodore de Sykéon.* Ed. A. J. Festugière. *Subsidia Hagiographica* 48 (Brussels 1970).

Theodore Studites = *Laudatio Platonis*. In PG99.

Theophanes = *Theophanis chronographia.* Ed. C. de Boor (Leipzig 1883).

Theophylact Simocatta, *Hist. = Theophylacti Simocattae historiae.* Ed. C. de Boor (Leipzig 1997).

Thomas of Margâ, Book of Governors = *The Book of Governors: the Historia monastica of Thomas, bishof of Margâ A.D. 840.* Ed. E. A. W. Budge (London 1893).

Victor of Tunnuna = *Chronica a. CCCCXLIV–DLXVII.* Ed. T. Mommsen, MGH AA 11, 184–206 (Berlin 1894).

Vita of Nicholas of Sion = *The Life of Saint Nicholas of Sion.* Eds. I. Ševčenko, N. P. Ševčenko (Brookline, 1984).

Vita of Symeon Stylites Iunior = *La vie ancienne de S. Syméon Stylite le jeune (521–592).* Ed. P. van den Ven (Brussels 1962).

Vita Willibaldi = Vitae Willibaldi et Wynnebaldi auctore sanctimoniali Heidenheimensi. Ed. O. Holder-Egger, MGH SS, 15/1 (Hannover 1887).

Zacharias Rhetor = *Historia Ecclesiastica.* Ed. E. W. Brooks, CSCO Syr. 3.6 (Louvain, 1924).

Inscriptions:

Byzantina Siciliae = *Byzantina Siciliae*. Ed. G. Manganaro. *Minima Epigraphica et Papyrologica* 4, 133 (2001).

Inscriptiones Christianae Urbis Romae = Inscriptiones Christianae Urbis Romae septimo seculo antiquiores, Nova Series (= ICUR NS) Eds. I.B. de Rossi et al. 10 vols. (Rome 1922–1992).

CIL = *Corpus Inscriptionum Latinarum* (Berlin 1863–).

Epigraphic material from Sufetula = Nouvelles recherches d'archéologie et d'épigraphie chrétienne à Sufetula (Byzacène). N. Duval. *Mélanges d'archéologie de l'École française de Rome* 68 (1956).

I. Palaestina Tertia = *Inscriptions from Palaestina Tertia*. Eds. Y. Meimaris, K. Kritikakou (Athens 2005).

Secondary sources:

L. I. Conrad: *The Plague in the Early Medieval Near East* (PhD, Princeton University 1981).

L. I. Conrad: Die Pest und ihr soziales Umfeld im Nahen Osten des frühen Mittelalters. *Der Islam* 73(1996): 81–112.

A. Dooley: The Plague and Its Consequences in Ireland. In: *Plague and the End of Antiquity: The Pandemic of 541–750.* Ed. L. K. Little (Cambridge 2007).

J.-N. Biraben, J. Le Goff: The Plague in the Early Middle Ages. In: *Biology of Man in History.* Ed. R. Forster and O. Ranum, trans. E. Forster and P. M. Ranum (Baltimore 1975).

K. Harper: *The Fate of Rome. Climate, Disease & the End of an Empire.* (Princeton, Oxford 2017).

J. Koder: Ein inschriftlicher Beleg zur Justinianischen Pest in Zora (Azra'a). Byzantinoslavica 56 (1995): 12–18.

M. Kulikowski: Plague in Spanish Late Antiquity. In: *Plague and the End of Antiquity: The Pandemic of 541–750.* Ed. L. K. Little (Cambridge 2007).

J. Maddicott: Plague in Seventh-Century England. In: *Plague and the End of Antiquity: The Pandemic of 541–750.* Ed. L. K. Little (Cambridge 2007).

M. McCormick: Tracking mass death during the fall of Rome's empire (II): a first inventory of mass graves. *Journal of Roman Archaeology* 29 (2016):1004–1046.

M . McCormick: Toward a Molecular History of the Justinianic Pandemic. In: *Plague and the End of Antiquity: The Pandemic of 541–750.* Ed. L. K. Little (Cambridge 2007).

E. Phillimore: The Annales Cambriae and Old-Welsh Genealogies from Harlein MS 3859. *Y Cymmrodor* 9 (1888):141–83.

D. C. Stathakopoulos: *Famine and Pestilence in the Late Roman and Early Byzantine Empire.* (Burlington 2004).

C. Tsiamis et al.: The Red Sea and the Port of Clysma. A Possible Gate of Justinian's Plague. *Gesnerus* 66 (2009):209–217.

D. Woods: *Acorns, the Plague and the 'Iona Chronicle.' Peritia* 17–18 (2003–4): 495–502.

Fig. S1: Heterozygosity plots for the twelve genomes with higher than 4.5-fold coverage. The y-axis displays percentage of allele frequency, the reference calls (0 %) and alternative calls (100 %) were excluded for scaling.

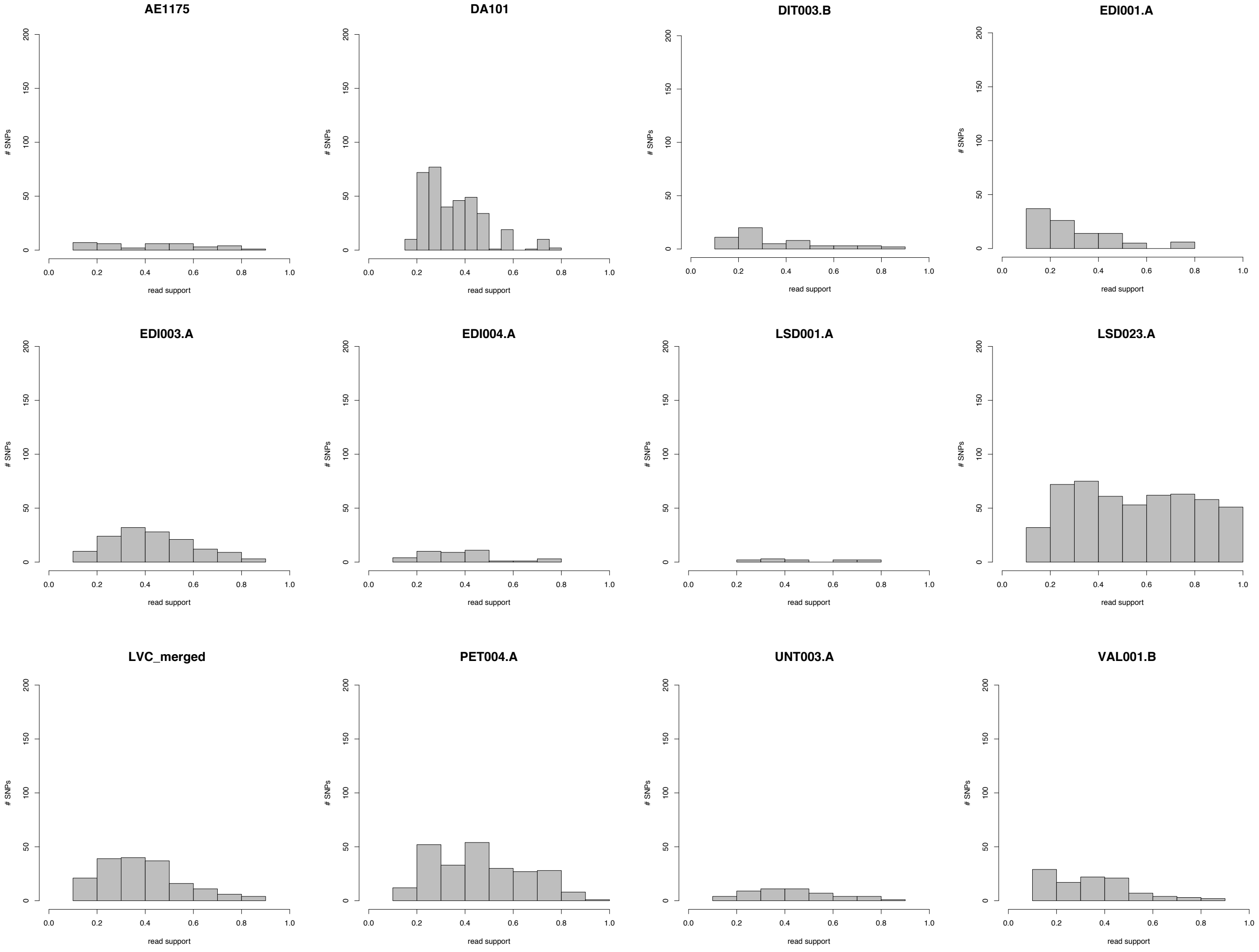

Fig. S2: Stacked bar chart for the false positive SNPs called in the background (DIR002.A) and the contaminated simulated datasets. The "Ns" are called due to heterozygosity (grey), all false positive SNPs were filtered out by our SNP evaluation (orange). With increasing coverage, the foreground has a high enough coverage to outshine the contamination and enable a reference call. See also SI Appendix, Table S6.

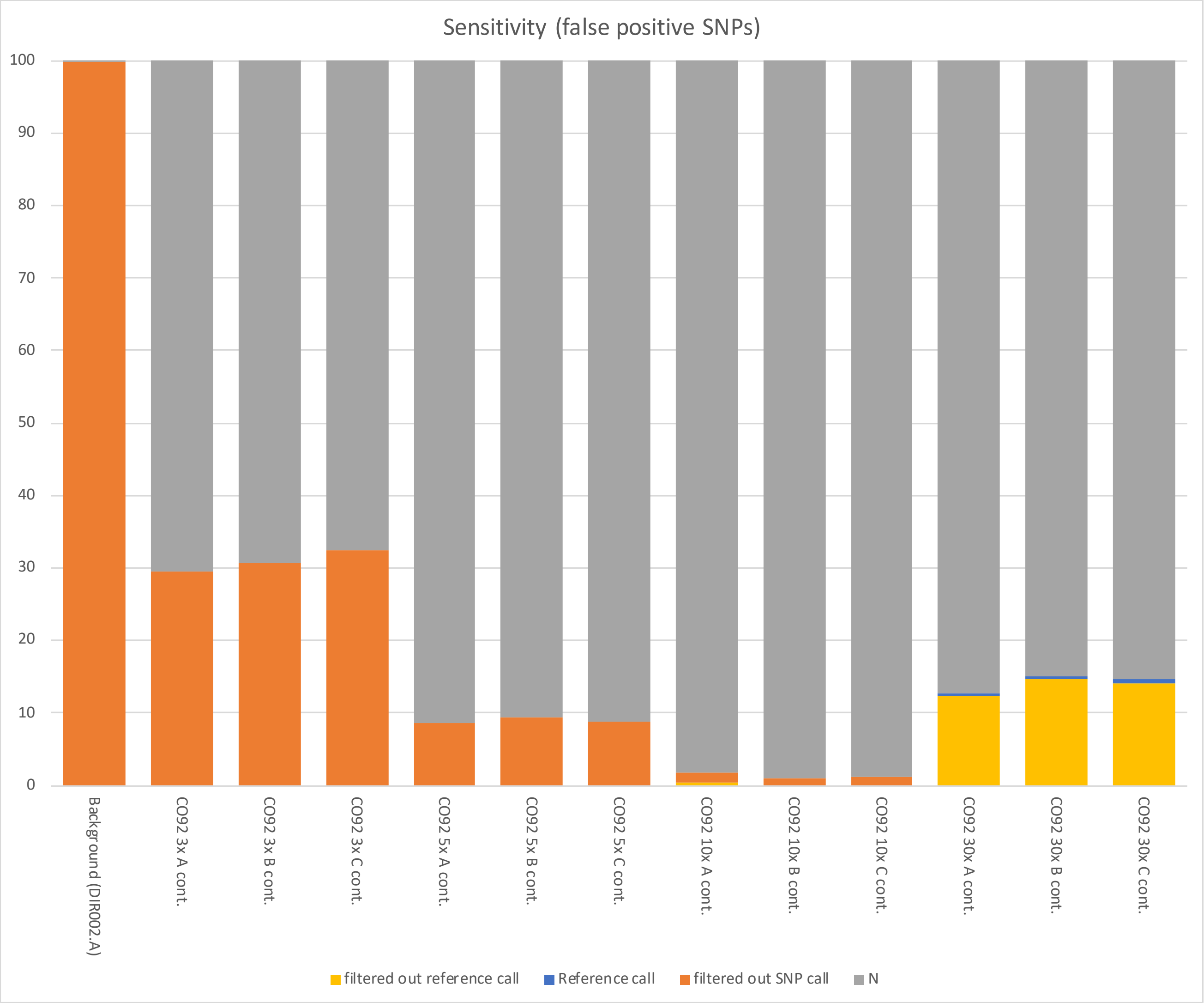

Fig. S3: Stacked bar chart for the 418 core positions for the backbone phylogeny of modern *Y. pestis* in the simulated datasets with ("Contaminated", right bar) and without ("Clean", left bar) background (DIR002.A). Whereas for the clean datasets, the number of "Ns" (grey) and filtered positions (orange) are only dependent on coverage and go to zero for the 30-fold coverage datasets, more positions are lost as "Ns" due to heterozygosity or filtered out due to contamination. See also SI Appendix, Table S7.

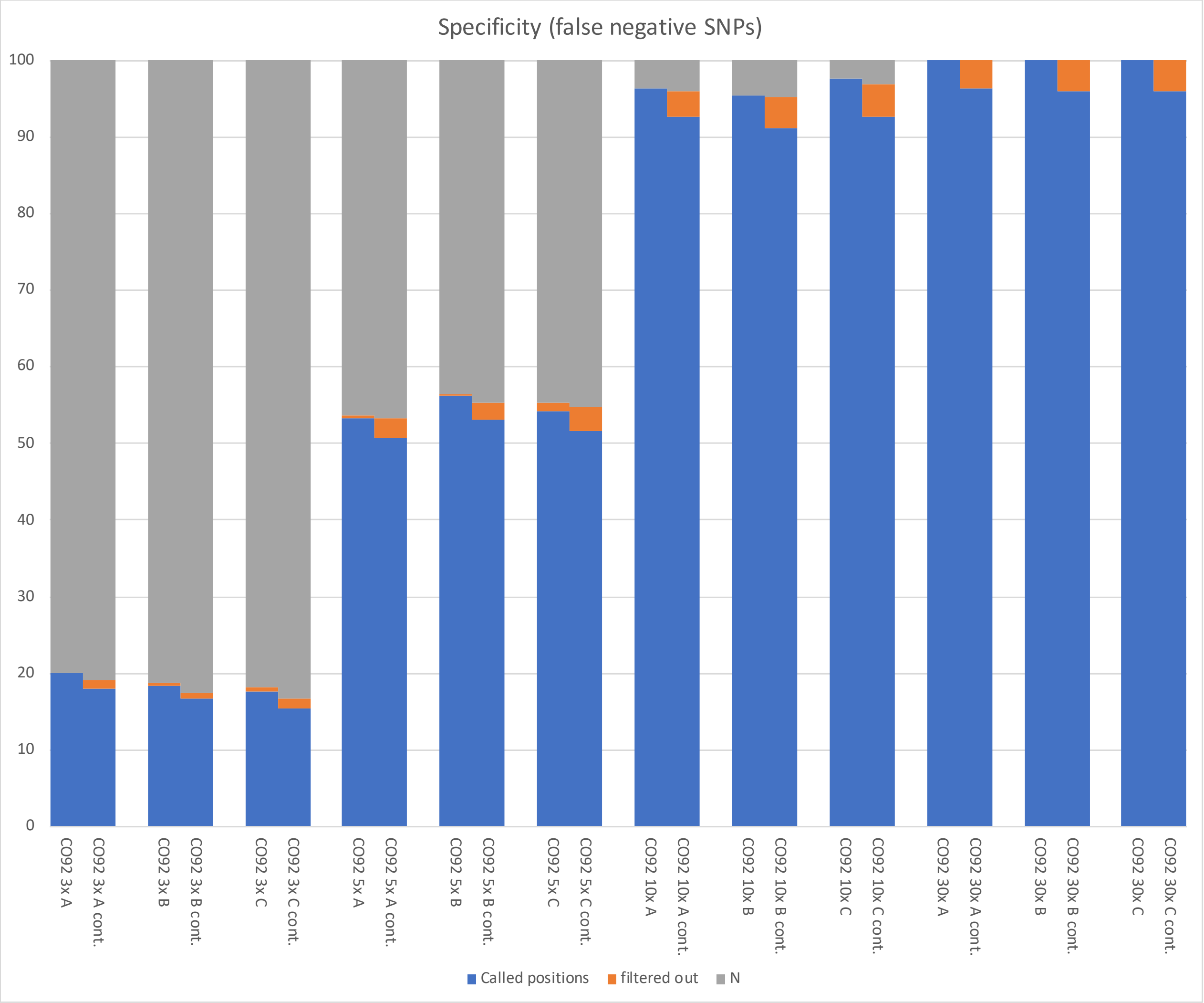

Fig. S4: Maximum Likelihood tree with full SNP alignment (6580 positions) of 233 modern *Y. pestis* and one *Y. pseudotuberculosis* genome, ten published (2nd–3rd century in orange; Altenerding in blue; Second Pandemic in red) and eight newly reconstructed genomes (green). Numbers on node are showing bootstrap values (1000 iterations).

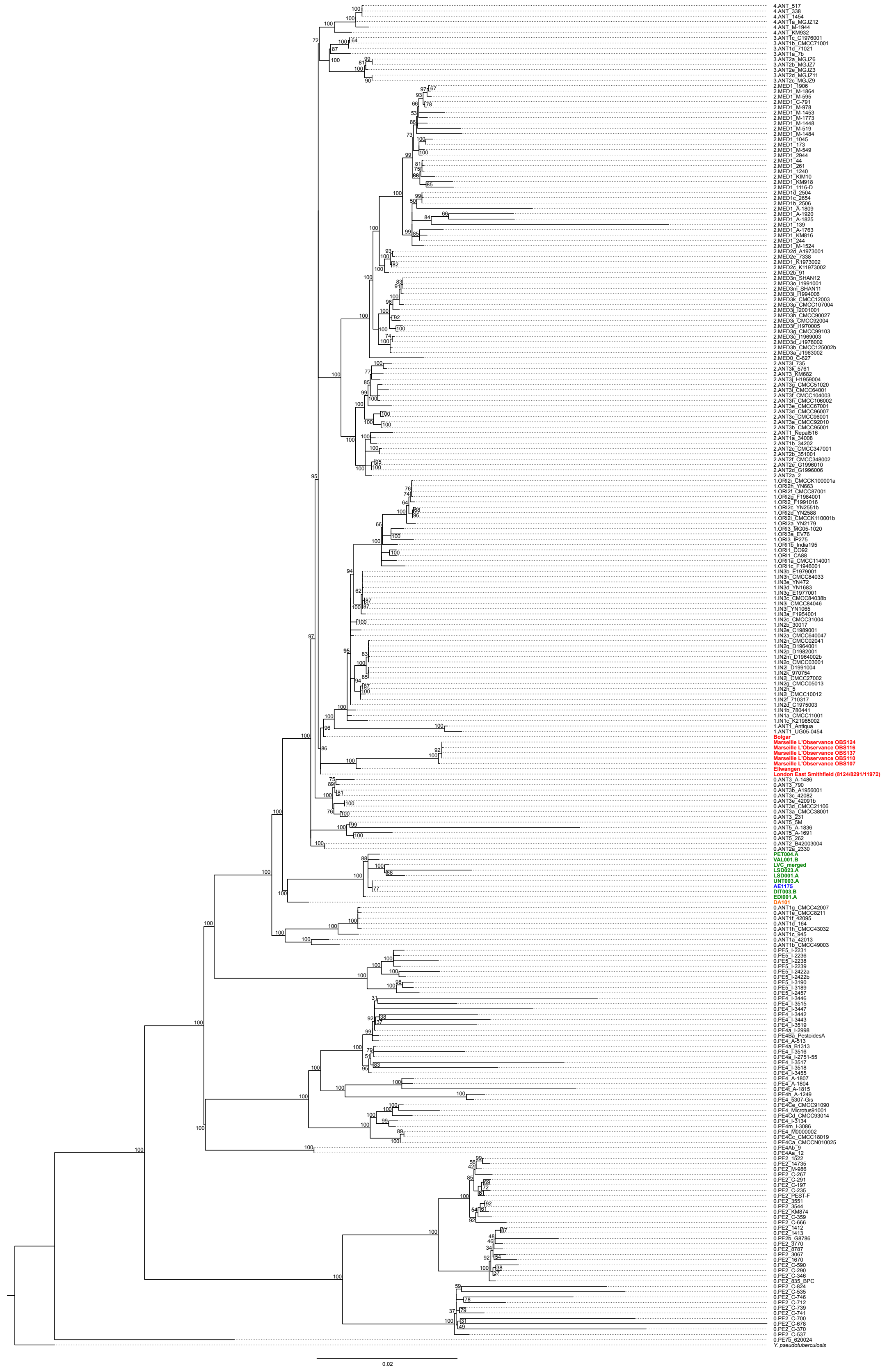

Fig. S5: Heatmap showing the percentage of coverage of plasmidal virulence factors in all analyzed First Pandemic genomes.

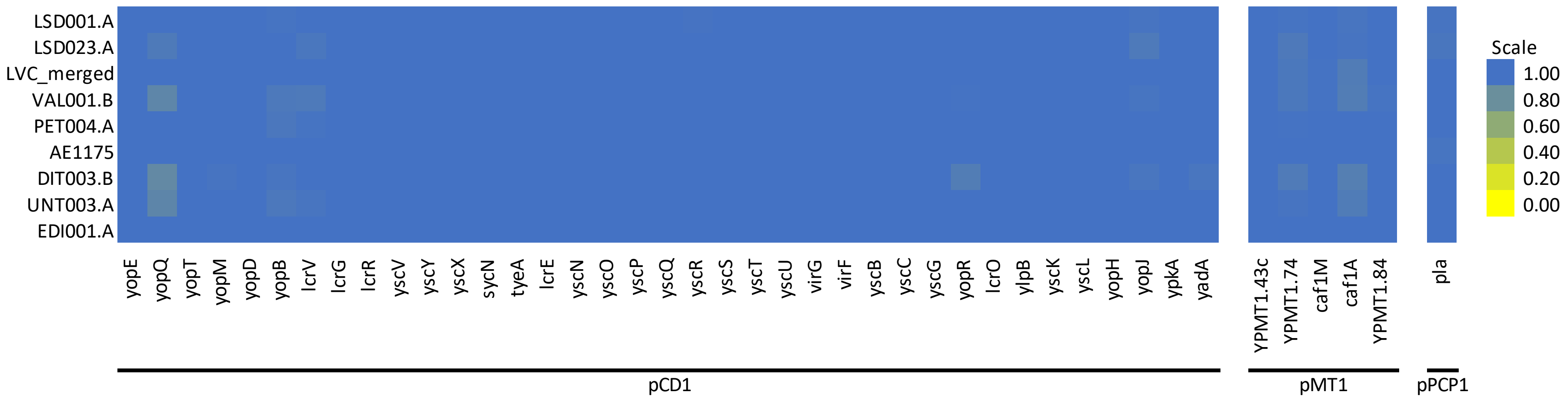

Fig. S6: Heatmap showing the percentage of coverage of the ca. 45 kb region of the chromosome deleted in LSD001.A, LSD023.A, LVC_merged and Marseille. First Pandemic genomes (blue and green) and Second Pandemic genomes (red) are shown in combination with selected strains of main clades of modern *Y. pestis* diversity on Branch 0 as well as the reference genomes of *Y. pseudotuberculosis* and *Y. pestis* (CO92).

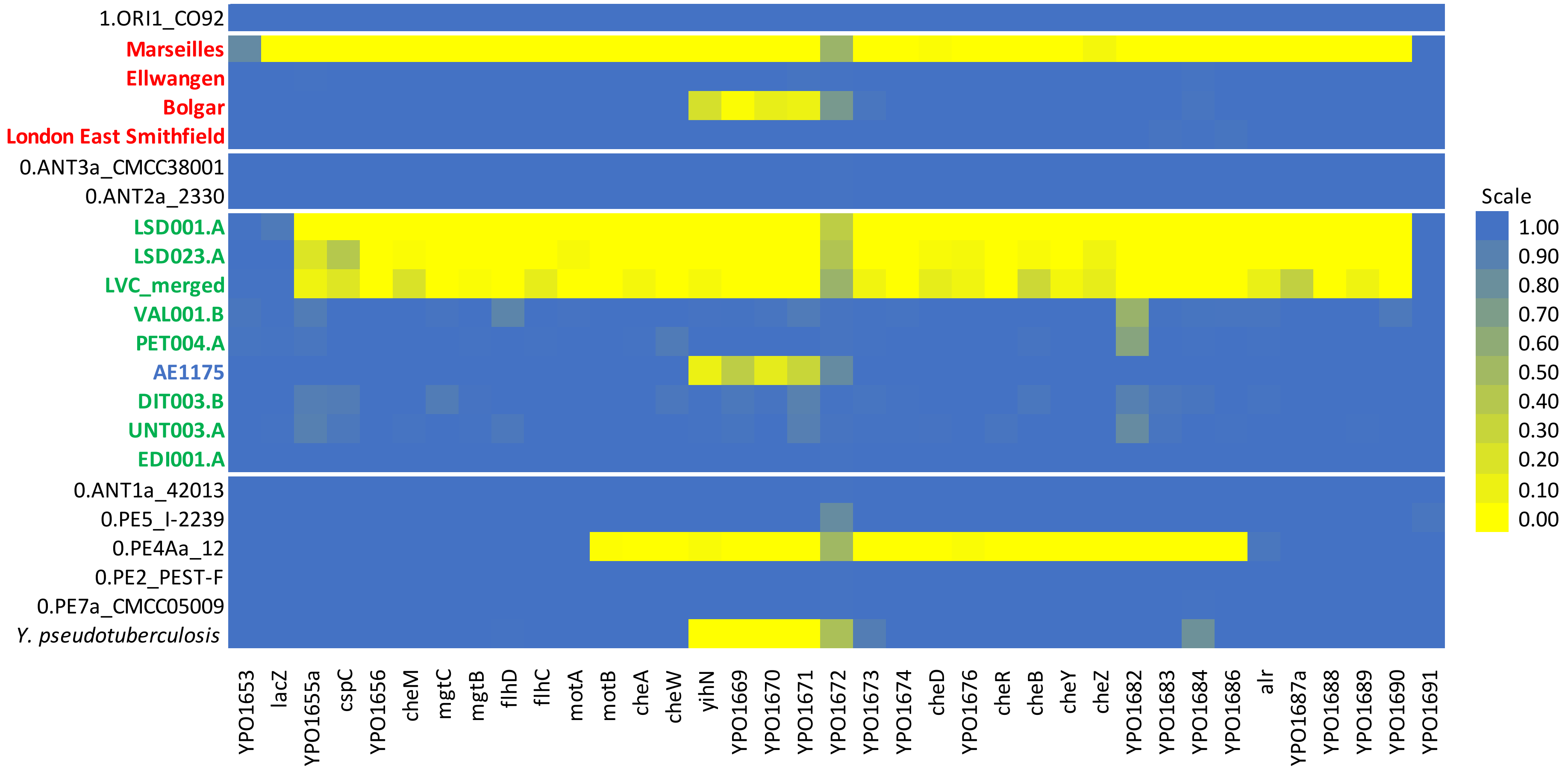

Fig. S7: Heatmap showing the percentage of coverage of a region on the chromosome (A) deleted in LSD001.A, LSD023.A and LVC_merged, and a region on the pMT1 plasmid (B) deleted in LSD001.A, LSD023.A, LVC_merged and UNT003.A.

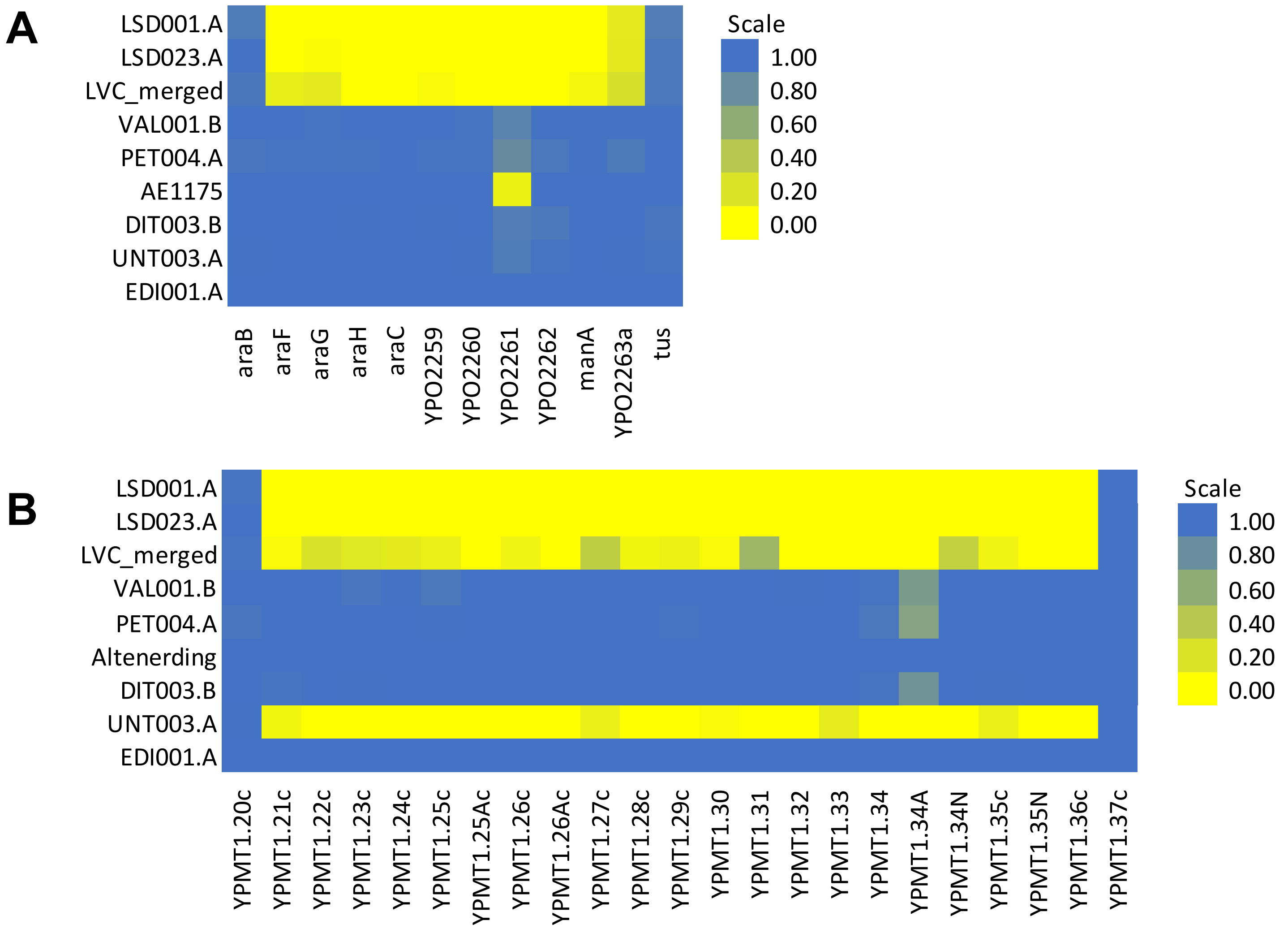

Fig. S8: Combined figure showing the probability plots for all radiocarbon samples (blue, 2-sigma ranges, see SI Appendix, Table S13 for details). The archaeological dating is shown as light blue box. Years with documented plague outbreaks are highlighted in red (Europe) and light orange (only Asia/Africa). The schematic tree of the presented genomes is shown on the left.

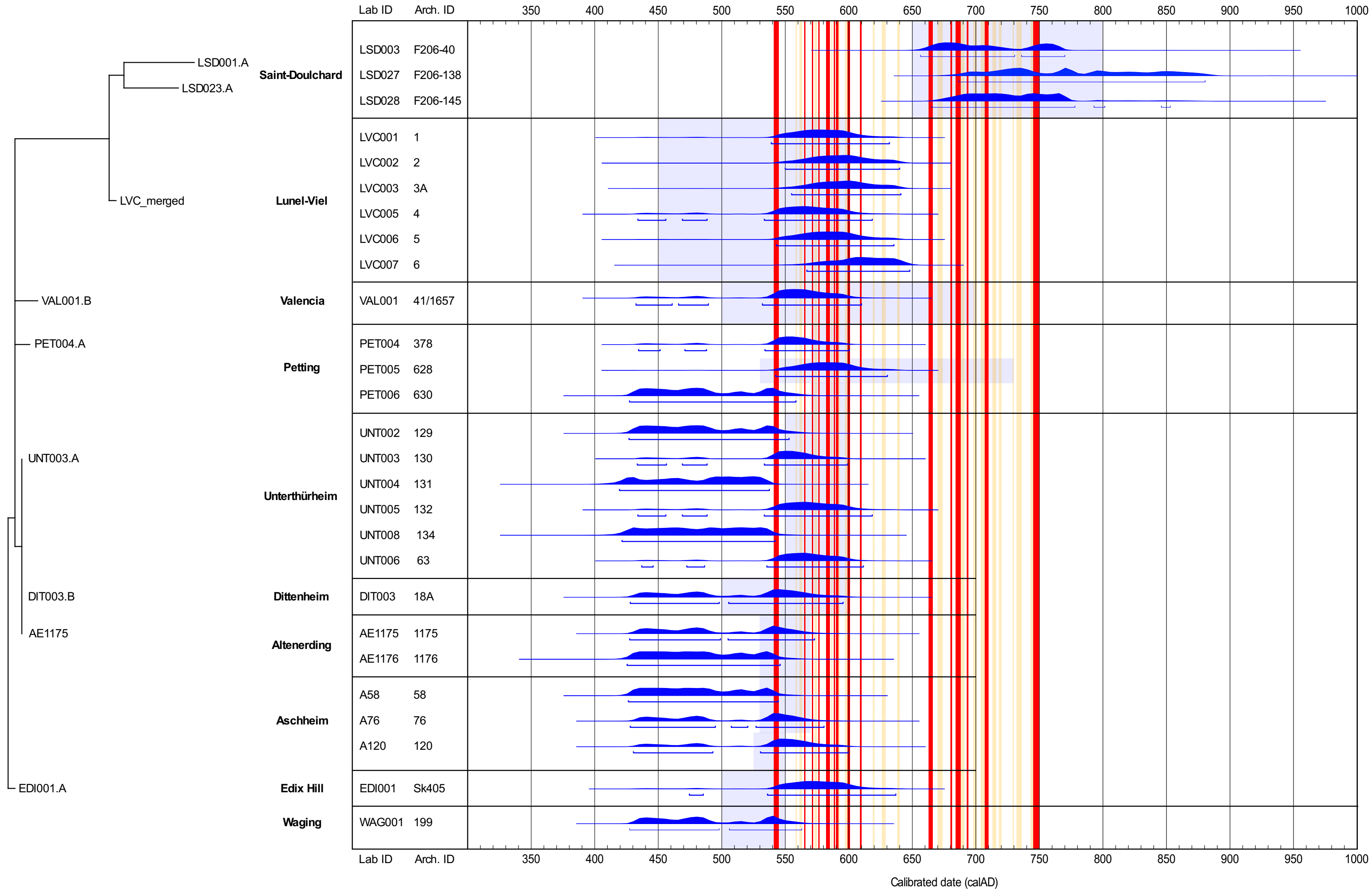

Fig. S9: Historical geography of the tested sites in Germany and Austria. A: Detailed map of all tested sites in Germany and Austria in relation to main rivers (blue), the Roman road network (orange) and the Roman provinces in their last state before the fall of the Western Roman Empire (shaded regions); all sources are given in the SI Appendix. The sites of published genomes are depicted by pink squares, the sites for the genomes presented here are represented by yellow squares. Sites tested negative are depicted in black upward-pointing triangles (burials dating before 541), squares (dating around 541–544), downward-pointing triangles (dating after 544). B: Relation of the detailed map (rectangular space) to the sites in Britain (Edix Hill), France (Lunel-Viel, Saint-Doulchard) and Spain (Valencia) presented in this study.

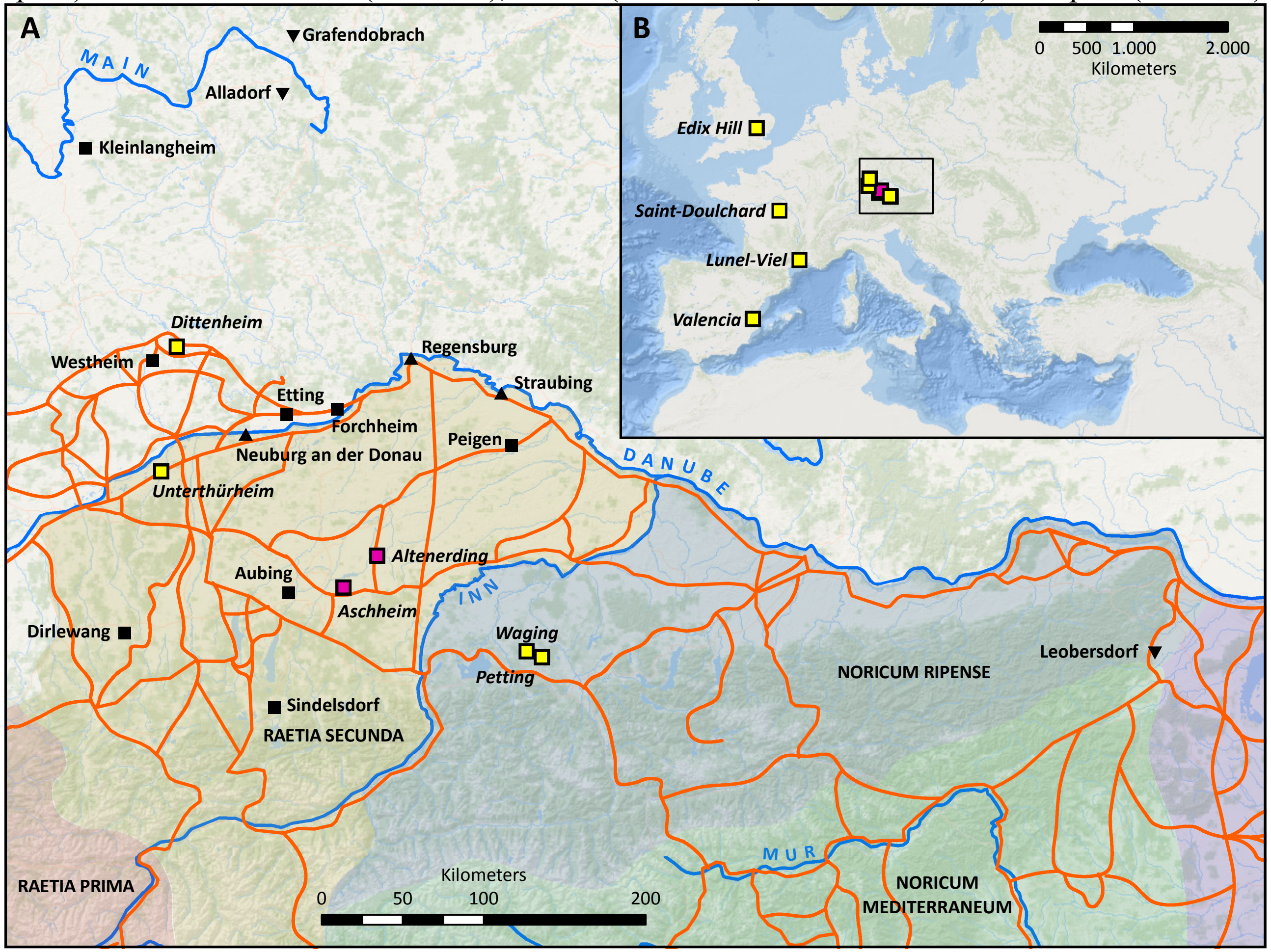

Fig. S10: Screenshot of IGV showing the positions 8528/8529 on the pPCP1 plasmid (175043/175044 in the merged reference) showing the 2-bp deletion causing mismapping.

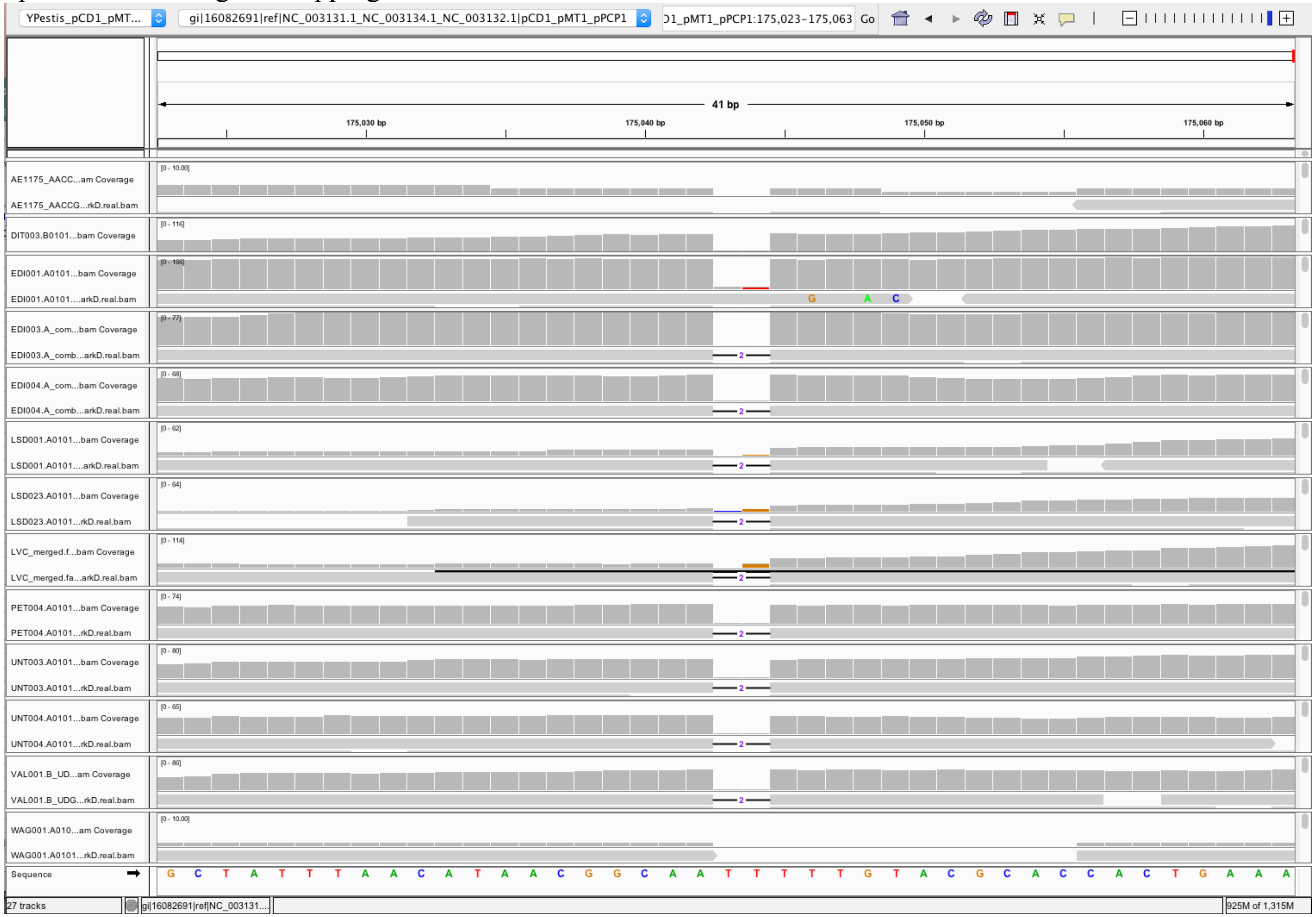

Table S1: Table of all tested individuals and samples. 'Group' gives the archaeological IDs of simultaneously buried individuals or individual from the same collective burial (Valencia: for details see site description, SI Apendix). 'Dating' gives the range of the archaeological dating CE for each burial. 'Total' list the total number of individuals, further broken down to adults (>20 ya) and subadults (<20 ya). 'Arch. ID' and 'Lab ID ind.' are showing the individual ID with the morphological 'sex'(f=female, m=male, ?=indeterminable) and 'age' (i=infans 1, ii=infans 2, j=juvenile, a=adult, m=mature, s=senile, (a)=at least adult, e=early, l=late). Teeth are noted in the FDI system or letters (I=Incisor, C=Canine, P=premolar, M=molar, lower case letters for deciduous teeth, indet=indeterminable). The qPCR screening results for *Y. pestis* are given in the 'Scr' column, samples screened with MALT are indicated by a star. The 'Seq' column shows if samples were considered as positive after sequencing.

Site	Group	Dating	Total	Adult	Sub-adult	Arch. ID	Lab ID ind.	Sex	Age	Features	Teeth	Lab ID sample	Scr	Seq
Alladorf	179/180	700–900	2	1	1	179	ALL001	f	ea		47	ALL001.A	neg	
						180	ALL002	?	i		64	ALL002.A	neg	
	184/185	700–900	2	1	1	184	ALL003	?	ii		85	ALL003.A	neg	
						185	ALL004	m	la–em		28	ALL004.A	neg	
	203/204	700–900	2	1	1	203	ALL005	m	la		18	ALL005.A	neg	
						204	ALL006	?	i	May be a later burial	74	ALL006.A	neg	
Dirlewang	33/34	650–700	2	1	1	33	DIR001	f	j–a		51	DIR001.A	neg	
	38/39	650–675	2	2	0	39	DIR002	f	a		17	DIR002.A	pos	neg
**Dittenheim**	188(A+B)	550–700	2	0	2	188A	DIT001	?	i		37	DIT001.A	neg	
						188B	DIT002	?	i		37	DIT002.A	neg	
	**18(A+B)**	**500–600**	**2**	**2**	**0**	**18A**	**DIT003**	**m**	**m**		18	DIT003.A	neg	
											**M**	**DIT003.B**	**pos**	**pos**
	**18(A+B)**	**500–600**	**2**	**2**	**0**	**18B**	**DIT004**	**f**	**a**		**17**	**DIT004.A**	**pos**	**pos**
											**M**	**DIT004.B**	**pos**	**pos**
	22(A+B)	600–700	2	1	1	22A	DIT005	?	i		85	DIT005.A	neg	
							DIT006	f	a		38	DIT006.A	neg	
	8(A+B)	500–700	2	2	0	8A	DIT007	f	m		18	DIT007.A	neg	

Site	Group	Dating	Total	Adult	Sub-adult	Arch. ID	Lab ID ind.	Sex	Age	Features	Teeth	Lab ID sample	Scr	Seq
**Edix Hill**	**Grave 76**	**500–550**	**1**	**0**	**1**	**Sk405**	**EDI001**	**?**	**j**		**M**	**EDI001.A**	**pos***	**pos**
	Grave 69	500–550	1	1	0	Sk359	EDI002	f	ea		M	EDI002.A	pos*	neg
	**Grave 78**	**500–550**	**1**	**0**	**1**	**Sk424**	**EDI003**	**?**	**j**		**M**	**EDI003.A**	**pos***	**pos**
	**Grave 96**	**500–550**	**2**	**1**	**1**	**Sk547B**	**EDI004**	**?**	**ii**	**Simultaneous double burial**	**M**	**EDI004.A**	**pos***	**pos**
	**Grave 106**	**500–550**	**2**	**2**	**0**	**Sk626A**	**EDI005**	**f**	**ea**	**Simultaneous double burial**	**M**	**EDI005.A**	**pos***	**pos**
						Sk626B	EDI008	m	ea	Simultaneous double burial	M	EDI008.A	neg	
	Grave 2	550–650	4	4	0	Sk3C	EDI009	m	ea	Sk3B and Sk3C simultaneous	M	EDI009.A	neg	
	Grave 9	500–550	2	1	1	Sk13A	EDI010	m	m-s	Simultaneous double burial	M	EDI010.A	neg	
	Grave 18	550–650	3	2	1	Sk42B	EDI006	f	ea	Nonsimultaneous double burial	M	EDI006.A	neg	
	Grave 46	500–550	1	1	0	Sk146	EDI011	m	em		M	EDI011.A	neg	
	Grave 60	550–650	1	1	0	Sk183	EDI012	f	a		M	EDI012.A	neg	
	Grave 63	500–650	1	1	0	Sk198	EDI013	m	ea		M	EDI013.A	neg	
	Grave 66	500–650	2	2	0	Sk322A	EDI014	m	(a)	Nonsimultaneous double burial	M	EDI014.A	neg	
	Grave 83	500–550	1	1	0	Sk436	EDI015	f	ea		M	EDI015.A	neg	
	Grave 84	500–550	2	1	1	Sk440A	EDI016	f	a	Simultaneous double burial	M	EDI016.A	neg	
	Grave 88	500–650	1	1	0	Sk453A	EDI017	m	ea		M	EDI017.A	neg	
	Grave 90	500–650	1	1	0	Sk458	EDI007	f	s		M	EDI007.A	neg	
	Grave 95	500–550	1	1	0	Sk530	EDI018	f	lm		M	EDI018.A	neg	
	Grave 97	550–650	1	1	0	Sk551	EDI019	m	ea		M	EDI019.A	neg	
	Grave 99	500–650	1	1	0	Sk576	EDI020	m	m		M	EDI020.A	neg	
	Grave 100	500–650	1	1	0	Sk578	EDI021	m	ea		M	EDI021.A	neg	
	Grave 105	500–650	1	1	0	Sk592	EDI022	f	lm		M	EDI022.A	neg	
Forchheim	1–4	650–700	4	2	1	1	FOR001	m	j		27	FOR001.A	neg	
						2	FOR002	f	m	Prone position	27	FOR002.A	neg	
						3	FOR003	m	m		47	FOR003.A	neg	
Grafendobrach	42/43/44	850–930	3	1	2	42	GRA001	m	es		46	GRA001.A	neg	
	83/84/85	850–930	3	2	1	83	GRA002	f?	em	May be a later burial	16	GRA002.A	neg	
						85	GRA003	f	ea		16	GRA003.A	neg	
Kleinlangheim	211/212	630–720	2	min 1	?	211	KLH001	m	m	Second burial: cremation	47	KLH001.A	neg	
	218/219	560–630	2	2	0	218	KLH002	m	ma–m		17	KLH002.A	neg	
						219	KLH003	f	la–em		23	KLH003.A	neg	
	272/273	670–720	2	2	0	273	KLH004	m	s		33	KLH004.A	neg	
	35/36	630–720	2	2	0	35	KLH005	m	a		37	KLH005.A	neg	
Leobersdorf	82(A–C)	770–800	3	1	2	82A	LEO001	m	j		36	LEO001.A	neg	
						82B	LEO002	f	a		27	LEO002.A	neg	
						82C	LEO003	?	i		55	LEO003.A	neg	

Site	Group	Dating	Total	Adult	Sub-adult	Arch. ID	Lab ID ind.	Sex	Age	Features	Teeth	Lab ID sample	Scr	Seq
**Saint-Doulchard**	**F 206-31**	**750–800?**	**1**	**1**	**0**	**31**	**LSD001**	**f**	**ea**		**47**	**LSD001.A**	**pos**	**pos**
	**F 206-38**		**1**	**1**	**0**	**38**	**LSD002**	**f**	**m**		**47**	**LSD002.A**	**pos**	**pos**
	F 206-40		1	1	0	40	LSD003	m	m		17	LSD003.A	neg	
	F 206-45		1	1	0	45	LSD004	m	m–s		17	LSD004.A	neg	
	F 206-51		1	1	0	51	LSD005	m	m		47	LSD005.A	neg	
	F 206-52		1	1	0	52	LSD006	?	ea		47	LSD006.A	neg	
	**F 206-65**		**1**	**1**	**0**	**65**	**LSD007**	**?**	**m–s**		**47**	**LSD007.A**	**pos**	**pos**
	F 206-68/68		2	1	1	68	LSD008	m	m–s		28	LSD008.A	neg	
	F 206-71/72		2	1	1	71	LSD009	m	s		37	LSD009.A	neg	
	F 206-78		1	1	0	78	LSD010	m	m–s		47	LSD010.A	neg	
	F 206-79		2	1	1	79a	LSD011	m	m–s		47	LSD011.A	neg	
	F 206-93/145		2	2	0	93	LSD012	?	ea		47	LSD012.A	neg	
	**F 206-132**		**1**	**1**	**0**	**132**	**LSD013**	**m**	**m**		**47**	**LSD013.A**	**pos**	**pos**
	F 206-136		1	1	0	136	LSD014	f	m		37	LSD014.A	neg	
	F 206-144		1	0	1	144	LSD015	?	j		47	LSD015.A	neg	
	F 206-151		1	1	0	151	LSD016	m	m		47	LSD016.A	neg	
	F 206-153		1	0	1	153	LSD017	?	ii		46	LSD017.A	neg	
	F 206-154		1	1	0	154	LSD018	?	ea		38	LSD018.A	neg	
	**F 206-155**		**2**	**2**	**0**	**155b**	**LSD019**	**m**	**m–s**		**48**	**LSD019.A**	**pos**	**pos**
	**F 206-156**		**2**	**1**	**1**	**156a**	**LSD020**	**m**	**m–s**		**47**	**LSD020.A**	**pos**	**pos**
	**F 206-172**		**3**	**1**	**2**	**172c**	**LSD021**	**f**	**m**		**38**	**LSD021.A**	**pos**	**pos**
						**172b**	**LSD022**	**?**	**j**		**46**	**LSD022.A**	**pos**	**pos**
	**F 206-204**		**2**	**1**	**1**	**204a**	**LSD023**	**m**	**m**		**47**	**LSD023.A**	**pos**	**pos**
	**F 206-208**		**1**	**0**	**1**	**208**	**LSD024**	**?**	**j**		**47**	**LSD024.A**	**pos**	**pos**
	F 206-215		1	1	0	215	LSD025	f	m–s		47	LSD025.A	neg	
	**F 206-216**		**1**	**0**	**1**	**216**	**LSD026**	**?**	**ii**		**36**	**LSD026.A**	**pos**	**pos**

Site	Group	Dating	Total	Adult	Sub-adult	Arch. ID	Lab ID ind.	Sex	Age	Features	Teeth	Lab ID sample	Scr	Seq
Lunel-Viel (Les Horts)	38(A–C)	475–550	3	3	0	38C	LVH001	?	(a)	Double burial with remains of a third individual	M	LVH001.A	neg	
											M	LVH001.B	neg	
											M	LVH001.C	neg	
											I	LVH001.D	neg	
											I	LVH001.E	neg	
**Lunel-Viel (Quartier centrale)**	**1–5**	**400–600**	**6**	**6**	**0**	**1**	**LVC001**	**f**	**ea**		M	LVC001.A	neg	
											**M**	**LVC001.B**	**pos**	**pos**
											**26**	**LVC001.C**	**pos**	**pos**
						2	LVC002	?	la–em		M	LVC002.A	neg	
											M	LVC002.B	neg	
						3A	LVC003	f	a		M	LVC003.A	neg	
											M	LVC003.B	neg	
						3B	LVC004	f	a		M	LVC004.A	neg	
											M	LVC004.B	neg	
						**4**	**LVC005**	**m**	**m**		**M**	**LVC005.A**	**pos**	**pos**
											**M**	**LVC005.B**	**pos**	**pos**
											**43**	**LVC005.C**	**pos**	**pos**
						**5**	**LVC006**	**m**	**ea**		**M**	**LVC006.A**	**pos**	**pos**
											M	LVC006.B	neg	
	6/7	400–600	2	0	2	6	LVC007	f	j–ea		M	LVC007.A	neg	
											M	LVC007.B	neg	
München-Aubing	675/676/677	650–700	3	2	1	676	AUB002	f	m		13	AUB002.A	neg	
						677	AUB003	m	?		16	AUB003.A	neg	
	724/725	400–700	2	2	0	724	AUB004	m	a		46	AUB004.A	neg	
						725	AUB005	m	m		31	AUB005.A	neg	
	809/810	400–700	2	2	0	809	AUB006	m	a		16	AUB006.A	neg	
						810	AUB007	m	a		36	AUB007.A	neg	
	854/855	650–700	2	2	0	854	AUB008	m	a		47	AUB008.A	neg	
	675/676/677	650–700	3	2	1	675	AUB009	?	i		85	AUB009.A	neg	
Neuburg an der Donau	34(N/M/S)	330–360	3	3	0	34M	NEU001	m	m		13	NEU001.A	neg	
						34S	NEU002	m	a		37	NEU002.A	neg	
Peigen	18(1–2)	600–700	2	2	0	18-1	PEI001	f	a	Opposite orientation	37	PEI001.A	pos	neg
						18-2	PEI002	f	ea	Opposite orientation	47	PEI002.A	neg	
	62(1–2)	600–650	2	1	1	62-2	PEI003	f	lm		38	PEI003.A	neg	
	63(1–2)	580–620	2	2	0	63-1	PEI004	f	a		47	PEI004.A	neg	
						63-2	PEI005	m	s		45	PEI005.A	neg	
**Petting**	342/343/344	530–730	3	2	1	342	PET001	m	ea		37	PET001.A	neg	
						343	PET002	?	ii		74	PET002.A	neg	
	**377/378**	**570–600**	**2**	**2**	**0**	377	PET003	m	la–m		26	PET003.A	neg	
						**378**	**PET004**	**m**	**la–em**		**36**	**PET004.A**	**pos**	**pos**
	**628**	**530–730**	**1**	**0**	**1**	**628**	**PET005**	**?**	**ii**	**Only a few remains, directly above 630/631**	**37**	**PET005.A**	**pos**	**pos**
	**630/631**	**570–600**	**2**	**2**	**0**	**630**	**PET006**	**f**	**ea**		**46**	**PET006.A**	**pos**	**pos**
						631	PET007	f	em		46	PET007.A	neg	

Site	Group	Dating	Total	Adult	Sub-adult	Arch. ID	Lab ID ind.	Sex	Age	Features	Teeth	Lab ID sample	Scr	Seq
Regensburg	30(1–2)	350–450	2	2	0	30-1	RFF001	m	la–em	Prone position	36	RFF001.A	neg	
	53(2/3)	350–450	2	1	1	53-2	RFF002	f	j	Crouched inhumation	26	RFF002.A	neg	
						53-3	RFF003	m	em	Crouched inhumation	46	RFF003.A	neg	
Sindelsdorf	162	630–720	1	1	0	162	SIN001	m	s	162 directly above 163–165	48	SIN001.A	neg	
	163–165	630–720	3	2	1	164	SIN002	m	la–em		17	SIN002.A	neg	
						165	SIN003	f?	m		47	SIN003.A	neg	
	51/52	630–670	2	2	0	52-1	SIN006	m	s		37	SIN006.A	neg	
						52-2	SIN007	m	m		33	SIN007.A	neg	
Straubing	16/17	300–400	2	0	2	16	SAZ001	?	i		85	SAZ001.A	neg	
	54a–c	300–400	3	1	2	54a	SAZ002	m?	j		26	SAZ002.A	neg	
						54b	SAZ003	m	j		27	SAZ003.A	neg	
**Unterthürheim**	116(1–2)	525–680	2	1	1	116(-2)	UNT001	?	ii		16	UNT001.A	neg	
	**129**	**550–600**	**1**	**0**	**1**	**129**	**UNT002**	**?**	**i**	**Close to 131–134**	**54**	**UNT002.A**	**pos**	**pos**
	**130**	**550–600**	**1**	**1**	**0**	**130**	**UNT003**	**m**	**m**	**Close to 131–134**	**37**	**UNT003.A**	**pos**	**pos**
	**131/132/133/134**	**550–600**	**4**	**2**	**2**	**131**	**UNT004**	**?**	**i**		**55**	**UNT004.A**	**pos**	**pos**
						**132**	**UNT005**	**m**	**a**		**48**	**UNT005.A**	**pos**	**pos**
	**63/64**	**550–600**	**2**	**1**	**1**	**63**	**UNT006**	**f**	**a**		**36**	**UNT006.A**	**pos**	**pos**
	63/64	550–600	2	1	1	64	UNT007	?	i		84	UNT007.A	neg	

Site	Group	Dating	Total	Adult	Sub-adult	Arch. ID	Lab ID ind.	Sex	Age	Features	Teeth	Lab ID sample	Scr	Seq
**Valencia**	**41**	**500–700**	**15**	**?**	**min 4**	**1657**	**VAL001**	**?**	**?**	**Collective burial**	M	VAL001.A	neg	
											**M**	**VAL001.B**	**pos**	**pos**
											P	VAL001.C	neg	
											m	VAL001.D	neg	
											P	VAL001.E	neg	
											m	VAL001.F	neg	
	40	500–700	4	?	?	1728	VAL002	?	?	Burial on apse entrance	I	VAL002.A	neg	
											M	VAL002.B	neg	
											C	VAL002.C	neg	
											indet.	VAL002.D	neg	
											indet.	VAL002.E	neg	
	50	500–700	7	min 2	min 1	10039	VAL003	?	?	Collective burial next to apse	P	VAL003.A	neg	
											M	VAL003.B	neg	
											M	VAL003.C	neg	
											I	VAL003.D	neg	
											I	VAL003.E	neg	
											I	VAL003.F	neg	
						10346	VAL004	?	?	Collective burial next to apse	C	VAL004.A	neg	
	28	500–700	min 21	min 13	min 3	10307	VAL005	?	?	Collective burial next to apse	M	VAL005.A	neg	
											M	VAL005.B	neg	
											M	VAL005.C	neg	
											M	VAL005.D	neg	
											M	VAL005.E	neg	
											M	VAL005.F	neg	
											M	VAL005.G	neg	
											M	VAL005.H	neg	
											Indet.	VAL005.I	neg	
											indet.	VAL005.J	neg	
											M	VAL005.K	neg	
											indet.	VAL005.L	neg	
						10313	VAL006	?	?	Collective burial next to apse	M	VAL006.A	neg	
											m	VAL006.B	neg	
						10322	VAL007	?	?	Collective burial next to apse	M	VAL007.A	neg	
	4	500–700	4	3	1	10066	VAL008	?	?	*Tegula* burial	M	VAL008.A	neg	
											27	VAL008.B	neg	
	28	500–700	min 21	min 13	min 3	10339	VAL009	?	?	Collective burial next to apse	27	VAL009.A	neg	

Site	Group	Dating	Total	Adult	Sub-adult	Arch. ID	Lab ID ind.	Sex	Age	Features	Teeth	Lab ID sample	Scr	Seq
**Waging**	**199**	**500–550**	**1**	**1**	**0**	**199**	**WAG001**	**m**	**ii**	**Superposition (under 200/201)**	**55**	**WAG001.A**	**pos**	**pos**
											M	WAG001.B	neg	
	200/201	600–700	2	1	1	200	WAG002	m	em		36	WAG002.A	neg	
											M	WAG002.B	neg	
						201	WAG003	?	ii		85	WAG003.A	neg	
											M	WAG003.B	neg	
	37(1–2)	600–700	2	1	1	37-1	WAG004	f?	ii	Superposition over 39, disturbed	46	WAG004.A	neg	
						37-2	WAG005	?	j–ea		62	WAG005.A	neg	
	39(N/S)	600–700	2	1	1	39N	WAG006	f	ii		75	WAG006.A	neg	
											m	WAG006.B	neg	
						39S	WAG007	f	lm		43	WAG007.A	neg	
											P	WAG007.B	neg	
Westheim	1	500–650	3	2	1	1(-1?)	WES001	m	la–em		27	WES001.A	neg	
						1(-2?)	WES002	f?	(a)		18	WES002.A	neg	
						1(-3?)	WES003	f	lm		26	WES003.A	neg	

Table S2: Mapping results of all UDG treated and captured samples for the chromosome and all three plasmids, giving the number of unique reads after clipping, merging, quality filtering and duplicate removal, and the mean coverage. In the three columns left of the last (showing the Mahalanobis distance), the ratio of mapping reads to the respective plasmid/chromosome is calculated for each sample.

Sample	Chrom. reads	Chrom. coverage	pCD1 reads	pCD1 coverage	pMT1 reads	pMT1 coverage	pPCP1 reads	pPCP1 coverage	Ratio reads pCD1/chrom.	Ratio reads pMT1/chrom.	Ratio reads pPCP1/chrom.	Mahalanobis distance
DIR002.A	8470	0.08	10	0.01	9	0.01	3	0.01	0.00118	0.00106	0.00035	8.80
DIT003.B	877150	9.41	31613	23.44	26800	14.12	7777	49.80	0.03604	0.03055	0.00887	0.61
DIT004.A	192557	1.65	7638	4.40	6383	2.74	2043	9.06	0.03967	0.03315	0.01061	0.77
DIT004.B	21824	0.22	849	0.58	935	0.46	220	1.12	0.03890	0.04284	0.01008	1.21
EDI001.A	2720235	38.05	66158	69.86	60478	43.68	10589	95.51	0.02432	0.02223	0.00389	1.36
EDI002.A	406	0.00	0	0	0	0	1	0.00	0.00000	0.00000	0.00246	9.36
EDI003.A	482945	5.17	21554	15.80	14738	7.80	6270	37.61	0.04463	0.03052	0.01298	2.95
EDI004.A	656840	7.50	21818	17.42	18463	10.55	5507	36.90	0.03322	0.02811	0.00838	0.57
EDI005.A	1706	0.02	19	0.02	24	0.01	14	0.07	0.01114	0.01407	0.00821	3.80
LSD001.A	426234	4.77	23345	17.55	22886	12.41	4539	25.77	0.05477	0.05369	0.01065	5.28
LSD002.A	3736	0.04	99	0.07	146	0.07	23	0.11	0.02650	0.03908	0.00616	2.63
LSD007.A	107157	1.05	4145	2.70	3774	1.80	519	2.65	0.03868	0.03522	0.00484	1.83
LSD013.A	2190	0.02	57	0.04	71	0.03	22	0.11	0.02603	0.03242	0.01005	0.85
LSD019.A	380773	3.93	15515	10.87	14445	7.44	1511	8.29	0.04075	0.03794	0.00397	2.65
LSD020.A	172504	1.76	6450	4.36	7697	3.79	1229	6.13	0.03739	0.04462	0.00712	2.25
LSD021.A	209598	2.08	6261	4.17	7690	3.70	1235	6.29	0.02987	0.03669	0.00589	1.05
LSD022.A	8536	0.10	303	0.24	352	0.20	42	0.25	0.03550	0.04124	0.00492	1.92
LSD023.A	591040	7.15	19573	16.25	17618	10.69	2320	15.28	0.03312	0.02981	0.00393	1.45
LSD024.A	207496	3.52	7371	8.22	6124	5.07	1771	15.05	0.03552	0.02951	0.00854	0.74
LSD026.A	1896	0.02	46	0.03	61	0.03	13	0.06	0.02426	0.03217	0.00686	1.03
LVC001.B	40317	0.41	1731	1.19	1731	0.86	1176	6.33	0.04293	0.04293	0.02917	9.14
LVC001.C	182392	1.78	8385	5.54	6156	2.96	5694	28.43	0.04597	0.03375	0.03122	10.26

Sample	Chrom. reads	Chrom. coverage	pCD1 reads	pCD1 coverage	pMT1 reads	pMT1 coverage	pPCP1 reads	pPCP1 coverage	Ratio reads pCD1/chrom.	Ratio reads pMT1/chrom.	Ratio reads pPCP1/chrom.	Mahalanobis distance
LVC005.A	162315	1.81	7300	5.56	5327	2.94	2022	12.14	0.04497	0.03282	0.01246	2.36
LVC005.B	3628	0.04	114	0.08	132	0.07	63	0.30	0.03142	0.03638	0.01736	2.48
LVC005.C	276945	2.68	8392	5.43	9594	4.55	6033	31.11	0.03030	0.03464	0.02178	5.19
LVC006.A	41524	0.43	1178	0.81	1673	0.85	277	1.44	0.02837	0.04029	0.00667	2.50
PEI001.A	1133	0.01	6	0.00	36	0.03	0	0.00	0.00530	0.03177	0.00000	11.07
PET004.A	515024	5.62	19971	14.86	17721	9.54	4442	26.78	0.03878	0.03441	0.00862	0.73
PET005.A	55916	0.49	2213	1.31	2395	1.04	589	2.62	0.03958	0.04283	0.01053	1.15
PET006.A	19144	0.20	645	0.46	746	0.38	209	1.09	0.03369	0.03897	0.01092	0.80
UNT002.A	14842	0.14	648	0.43	546	0.28	311	1.52	0.04366	0.03679	0.02095	2.62
UNT003.A	753340	7.57	21453	14.59	18224	9.13	5836	32.65	0.02848	0.02419	0.00775	0.72
UNT004.A	533323	5.19	20168	13.53	13211	6.45	4671	24.91	0.03782	0.02477	0.00876	3.02
UNT005.A	13669	0.15	578	0.40	448	0.24	171	0.90	0.04229	0.03277	0.01251	1.39
UNT006.A	7322	0.07	177	0.12	159	0.08	80	0.42	0.02417	0.02172	0.01093	1.13
VAL001.B	996679	9.62	27992	18.30	30071	14.29	5520	29.05	0.02809	0.03017	0.00554	0.42
WAG001.A	22904	0.23	733	0.50	539	0.26	351	1.93	0.03200	0.02353	0.01532	1.93

Table S3: Sequencing results for all verified *Y. pestis*-positive samples. The data of the Altenerding sample AE1175 was reprocessed without additional capture or sequencing. Data was merged in case multiple samples of the same individual were positive (DIT004, LVC001, LVC005) or when samples of multiple individuals of the same burial context were merged (LVC).

Sample	Reads prior mapping after adapter clipping and merging	Unique reads mapping to *Y. pestis* reference	Endogenous DNA (%)	Cluster Factor	Mean Coverage	Coverage 1X (%)	Median frag. Length
AE1175	67364973	1164051	3.56	2.06	17.17	93.62	64
DIT003.B	12699975	877150	22.88	3.31	9.41	91.41	48
DIT004.A	65225088	192557	1.02	3.44	1.65	69.02	38
DIT004.B	4361688	21824	1.80	3.59	0.22	17.41	45
DIT004 merged	70153762	215192	1.06	3.46	1.88	72.67	39
EDI001.A	13026793	2720235	25.15	1.20	38.05	95.24	60
EDI003.A	72165434	482945	1.00	1.49	5.17	91.62	47
EDI004.A	61443239	656840	1.47	1.38	7.50	93.92	47
EDI005.A	13587246	1706	0.02	1.77	0.02	0.52	38
LSD001.A	12697045	426234	3.80	1.13	4.77	88.41	49
LSD002.A	8163219	3736	0.05	1.12	0.04	3.49	44
LSD007.A	20960696	107157	1.79	3.50	1.05	49.74	43
LSD013.A	8836976	2190	0.03	1.21	0.02	1.8	42
LSD019.A	19755616	380773	5.15	2.67	3.93	85.09	45
LSD020.A	10566480	172504	1.95	1.20	1.76	70.87	45
LSD021.A	9622435	209598	2.82	1.29	2.08	72.64	43
LSD022.A	9748447	8536	0.10	1.15	0.10	9.07	50
LSD023.A	20406195	591040	6.78	2.34	7.15	90.37	51
LSD024.A	22016115	207496	5.47	5.80	3.52	85.63	76
LSD026.A	8778616	1896	0.03	1.18	0.02	1.52	43
LVC001.B	3593715	40317	3.65	3.25	0.41	28.23	45
LVC001.C	16970946	182392	5.76	3.29	1.78	72.48	43
LVC001 merged	20564661	220514	3.56	3.32	2.17	76.42	43
LVC005.A	3299048	162315	14.12	2.87	1.81	70.13	50
LVC005.B	3634255	3628	0.27	2.75	0.04	3.09	44
LVC005.C	20373965	276945	7.44	3.61	2.68	80.19	43
LVC005 merged	27307268	430854	5.40	3.43	4.42	86.67	45
LVC006.A	4480909	41524	3.29	3.55	0.43	29.66	46
LVC merged	52352838	658940	4.50	3.57	6.71	89.89	45
VAL001.B	65109736	996679	4.28	2.80	9.62	91.35	43

Sample	Reads prior mapping after adapter clipping and merging	Unique reads mapping to *Y. pestis* reference	Endogenous DNA (%)	Cluster Factor	Mean Coverage	Coverage 1X (%)	Median frag. Length
PET004.A	52013500	515024	2.69	2.72	5.62	92.30	48
PET005.A	89496419	55916	0.34	5.51	0.49	34.62	39
PET006.A	48802311	19144	0.12	3.02	0.20	16.10	44
UNT002.A	88974245	14842	0.20	12.15	0.14	10.97	41
UNT003.A	37303508	753340	6.11	3.03	7.57	92.65	44
UNT004.A	26118776	533323	7.07	3.46	5.19	91.22	42
UNT005.A	3613234	13669	0.49	1.29	0.15	13.24	45
UNT006.A	91966889	7322	0.08	9.64	0.07	4.98	40
WAG001.A	74283932	22904	0.13	4.31	0.23	13.89	43

Table S4: Number of reads of EDI samples assigned to the *Y. pseudotuberculosis* complex/*Y. pestis* node in MALT with 85 % identity.

Samples	Total # of reads	*Y. pseudotuberculosis* complex node		*Y. pestis* node
		assigned	summed	summed
EDI001.A	14183537	26478	36623	9886
EDI002.A	10714470	44	58	14
EDI003.A	41364951	3485	4738	1204
EDI004.A	5792548	743	991	230
EDI005.A	9029341	21	33	4
EDI006.A	7838268	6	10	1
EDI007.A	44427907	21	27	1
EDI008.A	4544207	79	110	29
EDI009.A	9918621	12	25	2
EDI010.A	4767075	0	0	0
EDI011.A	8639291	4	7	0
EDI012.A	38492923	145	259	29
EDI013.A	17140889	7	8	1
EDI014.A	8189593	0	2	0
EDI015.A	10412796	5	10	1
EDI016.A	27869518	44	69	6
EDI017.A	9565231	10	16	0
EDI018.A	2040312	3	4	1
EDI019.A	10904548	2	6	2
EDI020.A	29162638	29	45	2
EDI021.A	10637817	4	9	1
EDI022.A	9585693	23	31	4

Table S5: Basic statistics for the background (DIR002.A) and the simulated datasets (CO92) with targeted mean coverages of 3-fold, 5-fold, 10-fold and 30-fold, as well as for the datasets after merging with the background (cont.=contaminated).

Sample Name	Reads prior mapping after adapter clipping and merging	Unique reads mapping to Y. pestis reference	Endogenous DNA (%)	Cluster Factor	Mean Coverage	Coverage 1X (%)	Median frag. Length
DIR002.A	3728460	8470	0.44	1.94	0.08	1.07	41
CO92 3x A	279387	263585	94.38	1.00	2.83	89.94	48
CO92 3x B	279376	263413	94.32	1.00	2.83	90.05	48
CO92 3x C	279392	263532	94.36	1.00	2.83	89.97	48
Average	279385.00	263510.00	94.35	1.00	2.83	89.99	48
CO92 5x A	465651	438732	94.28	1.00	4.71	94.24	48
CO92 5x B	465655	439019	94.35	1.00	4.72	94.23	48
CO92 5x C	465642	438804	94.30	1.00	4.72	94.26	48
Average	465649.33	438851.67	94.31	1.00	4.72	94.24	48
CO92 10x A	931299	877513	94.35	1.00	9.43	95.06	48
CO92 10x B	931295	877631	94.36	1.00	9.43	95.07	48
CO92 10x C	931290	877187	94.31	1.00	9.43	95.07	48
Average	931294.67	877443.67	94.34	1.00	9.43	95.07	48
CO92 30x A	2793853	2625000	94.32	1.00	28.20	95.24	48
CO92 30x B	2793896	2625351	94.33	1.00	28.21	95.26	48
CO92 30x C	2793887	2625415	94.33	1.00	28.21	95.25	48
Average	2793878.67	2625255.33	94.33	1.00	28.20	95.25	48
CO92 3x A cont.	4007847	267836	6.99	1.05	2.87	90.00	48
CO92 3x B cont.	4007836	267830	6.99	1.05	2.87	90.12	48
CO92 3x C cont.	4007852	267963	6.99	1.05	2.88	90.05	48
Average	4007845.00	267876.33	6.99	1.05	2.87	90.06	48
CO92 5x A cont.	4194111	436364	10.86	1.04	4.69	94.26	48
CO92 5x B cont.	4194115	436628	10.87	1.04	4.70	94.25	48
CO92 5x C cont.	4194102	436485	10.86	1.04	4.70	94.28	48
Average	4194109.33	436492.33	10.86	1.04	4.70	94.26	48
CO92 10x A cont.	4659759	843943	19.21	1.06	9.12	95.07	48
CO92 10x B cont.	4659755	844112	19.21	1.06	9.12	95.08	48
CO92 10x C cont.	4659750	843602	19.20	1.06	9.12	95.07	48
Average	4659754.67	843885.67	19.21	1.06	9.12	95.07	48
CO92 30x A cont.	6522313	2283270	40.66	1.16	25.01	95.25	49
CO92 30x B cont.	6522356	2283502	40.66	1.16	25.02	95.27	49
CO92 30x C cont.	6522347	2283869	40.66	1.16	25.02	95.25	49
Average	6522338.67	2283547.00	40.66	1.16	25.02	95.26	49

Table S6: Numbers of false positive SNPs introduced by the background (DIR002.A) in the simulated datasets (see also SI Appendix, Fig. S2). "Filtered out" gives the number of false positive SNPs that do not pass our SNP evaluation criteria. In this table, "Ns" are equivalent to heterozygous positions.

	Background	CO92 3x A cont.	CO92 3x B cont.	CO92 3x C cont.	Average	CO92 5x A cont.	CO92 5x B cont.	CO92 5x C cont.	Average	CO92 10x A cont.	CO92 10x B cont.	CO92 10x C cont.	Average	CO92 30x A cont.	CO92 30x B cont.	CO92 30x C cont.	Average
N	1	319	313	305	312.33	413	410	412	411.67	444	448	447	446.33	395	384	386	388.33
Reference call	0	0	0	0	0.00	0	0	0	0.00	2	0	0	0.67	57	68	66	63.67
filtered out reference call	0	0	0	0	0.00	0	0	0	0.00	2	0	0	0.67	56	66	64	62.00
SNP call	451	133	139	147	139.67	39	42	40	40.33	6	4	5	5.00	0	0	0	0.00
filtered out SNP call	451	133	139	147	139.67	39	42	40	40.33	6	4	5	5.00	0	0	0	0.00

Table S7: Numbers of 418 core positions for the backbone phylogeny of modern *Y. pestis* in the simulated datasets with ("Contaminated") and without ("Clean") background (DIR002.A); see also SI Appendix, Fig. S3. Positions were either called as "reference call" or "Ns" representing positions not called due to low coverage or heterozygosity. "Positions filtered out" give the number of positions that did not pass our SNP evaluation. The positions "lost by heterozygosity" give the difference of reference calls (contaminated) to reference calls (clean). The positions "lost by contamination" give the difference of remaining calls after filtering (contaminated) to remaining calls after filtering (clean).

		DIR002.A	CO92 3x A	CO92 3x B	CO92 3x C	Average	CO92 5x A	CO92 5x B	CO92 5x C	Average	CO92 10x A	CO92 10x B	CO92 10x C	Average	CO92 30x A	CO92 30x B	CO92 30x C	Average
**Clean**																		
N		-	334	340	342	338.67	194	182	187	187.67	15	19	10	14.67	0	0	0	0.00
Reference call		-	84	78	76	79.33	224	236	231	230.33	403	399	408	403.33	418	418	418	418.00
filtered out		-	0	1	2	1.00	1	1	4	2.00	0	0	0	0.00	0	0	0	0.00
remaining		-	84	77	74	78.33	223	235	227	228.33	403	399	408	403.33	418	418	418	418.00
lost by filtering		-	0.00 %	1.28 %	2.63 %	1.26 %	0.45 %	0.42 %	1.73 %	0.87 %	0.00 %	0.00 %	0.00 %	0.00 %	0.00 %	0.00 %	0.00 %	0.00 %
**Contaminated**																		
N		416	338	345	348	343.67	195	187	189	190.33	17	20	13	16.67	0	0	0	0.00
Reference call		2	80	73	70	74.33	223	231	229	227.67	401	398	405	401.33	418	418	418	418.00
filtered out		2	5	3	6	4.67	11	9	13	11.00	14	17	18	16.33	15	17	17	16.33
remaining		0	75	70	64	69.67	212	222	216	216.67	387	381	387	385.00	403	401	401	401.67
lost by filtering		100.00 %	6.25 %	4.11 %	8.57 %	6.28 %	4.93 %	3.90 %	5.68 %	4.83 %	3.49 %	4.27 %	4.44 %	4.07 %	3.59 %	4.07 %	4.07 %	3.91 %
lost by heterozygosity	#	-	4	5	6	5.00	1	5	2	2.67	2	1	3	2.00	0	0	0	0.00
	%	-	4.76 %	6.41 %	7.89 %	6.30 %	0.45 %	2.12 %	0.87 %	1.16 %	0.50 %	0.25 %	0.74 %	0.50 %	0.00 %	0.00 %	0.00 %	0.00 %
lost by contamination	#	-	9	7	10	8.67	11	13	11	11.67	16	18	21	18.33	15	17	17	16.33
	%	-	10.71 %	9.09 %	13.51 %	11.06 %	4.93 %	5.53 %	4.85 %	5.11 %	3.97 %	4.51 %	5.15 %	4.55 %	3.59 %	4.07 %	4.07 %	3.91 %

Table S8: Table with all non-shared chromosomal SNPs that were called in the investigated First Pandemic genomes, sorted by genome (second cell in header). 'Position' refers to the position in the reference genome CO92, followed by the respective SNP call and the reference call. The classification as potential false or true positive is based on the preceding parameters regarding a 50 bp window surrounding the SNP: Only SNPs with no uncovered position, no heterozygous SNP and an equal mean coverage with high and low stringent mapping (ratio of 1.00) are classified as true positive SNPs (highlighted in green). If a SNP is ambiguous due to conflicting classification in different samples, this is indicated with a question mark highlighted in orange. The 'comment' column with additional classification is followed by the respective calls in the other First Pandemic genomes (0=not covered, N=heterozygous, lower case letters=less than 3 reads coverage). The last column is giving the corresponding genomes, if the SNP appears as homoplastic in the SNP table.

**Position**	**AE1175 call**	Reference call	Uncovered positions in 50 bp	Heterozygous SNPs in 50 bp	Mean coverage ratio LS/HS	Classification	Comment	DIT003.B	EDI001.A	EDI003.A	EDI004.A	LSD001.A	LSD023.A	LVC_merged	PET004.A	UNT003.A	UNT004.A	VAL001.B	WAG001.A	Homoplasy
20769	G	A	0	1	1.00	false positive		0	N	a	0	0	0	0	A	0	a	0	0	2.MED2e_7338
420208	T	G	20	0	1.00	false positive	Feldman et al. 2016: Called as SNP but not called as unique due to homoplasy	0	t	t	t	0	0	0	0	0	0	0	0	0.PE2_6904
549767	C	T	0	0	1.45	false positive	Feldman et al. 2016	C	T	T	T	c	c	c	c	C	C	c	0	
2348782	T	C	5	0	1.00	false positive		0	t	0	t	t	0	0	0	t	0	t	0	
2828136	G	A	0	3	1.39	false positive		N	N	a	0	a	0	0	0	a	a	g	0	
2980096	T	G	0	1	1.38	false positive		t	N	g	g	t	N	g	0	0	0	g	0	2.ANT2b_351001
2980097	G	A	0	1	1.37	false positive		g	N	a	a	g	N	a	0	0	0	a	0	2.ANT2b_351001
3179828	A	C	0	0	1.00	true positive	shared AE, DIT, UNT	A	C	C	C	c	C	C	C	A	A	C	0	
3223354	T	G	0	1	1.13	false positive		0	N	0	N	0	0	0	0	0	g	0	0	1.IN2i_CMCC10012
3223359	A	C	0	1	1.13	false positive		0	N	0	N	0	0	0	a	0	c	0	0	1.IN2i_CMCC10012, 1.IN1a_CMCC11001
3225856	A	C	23	0	1.24	false positive		a	N	a	0	0	0	0	0	c	a	c	0	
3588894	C	A	0	1	1.17	false positive		a	A	0	0	0	0	a	0	0	0	a	0	
3750736	A	G	0	0	0.99	true positive?	false positive in VAL001.B, LSD001.A, shared AE, DIT, LSD, LVC, PET, UNT, VAL	a	G	G	G	A	A	a	A	A	A	A	a	

**Position**	**AE1175 call**	Reference call	Uncovered positions in 50 bp	Heterozygous SNPs in 50 bp	Mean coverage ratio LS/HS	Classification	Comment	DIT003.B	EDI001.A	EDI003.A	EDI004.A	LSD001.A	LSD023.A	LVC_merged	PET004.A	UNT003.A	UNT004.A	VAL001.B	WAG001.A	Homoplasy
3755861	T	C	0	0	1.00	true positive	Feldman et al. 2016, potentially shared ancestral	0	T	t	0	0	t	t	t	t	t	0	0	
4412624	G	A	0	0	1.00	true positive?	Feldman et al. 2016, false positive in UNT003.A, true positive in PET, potentially shared DIT, LSD, PET, UNT, VAL	0	A	0	a	g	g	0	G	G	g	g	0	

Position	**DIT003.B call**	Reference call	Uncovered positions in 50 bp	Heterozygous SNPs in 50 bp	Mean coverage ratio LS/HS	Classification	Comment	AE1175	EDI001.A	EDI003.A	EDI004.A	LSD001.A	LSD023.A	LVC_merged	PET004.A	UNT003.A	UNT004.A	VAL001.B	WAG001.A	Homoplasy
335336	C	T	0	1	1.00	false positive		N	N	N	N	N	N	0	0	N	N	c	0	0.PE4Cd_CMCC93014, 2.ANT1_Nepal516, 0.PE4_Microtus91001, 0.PE5_6213, 0.PE4_6216, *Y. pseudotuberculosis*
549767	C	T	0	0	1.17	false positive		C	T	T	T	N	c	c	c	C	C	c	0	
944177	G	C	6	0	1.00	false positive		N	N	g	N	c	c	c	c	N	N	N	0	
944178	A	G	6	0	1.00	false positive		N	N	a	g	g	g	g	g	N	g	N	0	
3179828	A	C	0	0	1.00	true positive	shared AE, DIT, UNT	A	C	C	C	c	C	C	C	A	A	C	0	
3225949	G	A	21	0	1.12	false positive		g	G	g	g	0	g	a	0	0	0	g	0	
3890928	G	C	4	0	1.03	false positive		N	N	c	c	g	G	N	N	g	N	G	0	
4232217	T	C	4	0	1.00	false positive		0	T	t	t	t	T	c	0	t	0	0	0	0.PE2_1522

Position	**EDI001.A call**	Reference call	Uncovered positions in 50 bp	Heterozygous SNPs in 50 bp	Mean coverage ratio LS/HS	Classification	Comment	AE1175	DIT003.B	EDI003.A	EDI004.A	LSD001.A	LSD023.A	LVC_merged	PET004.A	UNT003.A	UNT004.A	VAL001.B	WAG001.A	Homoplasy
698935	G	T	0	2	1.09	false positive		t	t	0	0	0	0	t	0	t	0	t	0	
718827	C	T	14	0	1.29	false positive		c	c	0	c	0	c	c	c	c	c	C	0	Branch 2
1105569	T	A	0	2	1.23	false positive		N	a	a	0	0	0	a	0	0	a	a	0	
1105570	C	T	0	2	1.24	false positive		N	g	0	0	0	0	0	0	0	0	0	0	
1444672	G	A	0	1	1.13	false positive?	true positive in EDI004.A, potentially shared ancestral	g	0	G	G	g	G	g	N	g	g	0	0	
1599102	A	T	2	0	1.14	false positive		a	0	t	0	0	0	0	t	0	0	a	0	
1722572	A	G	1	2	1.37	false positive		a	0	N	a	a	0	0	0	a	a	0	0	
2576207	G	A	0	1	1.29	false positive		a	N	0	g	0	N	g	g	g	g	g	0	
2801707	A	G	0	0	1.05	false positive?	true positive in EDI003.A	G	G	A	a	G	G	G	G	G	g	G	0	
3005046	T	A	22	0	1.00	false positive		t	0	0	0	0	t	0	0	t	t	t	0	
3225949	G	A	21	0	1.10	false positive		g	G	g	g	0	g	a	0	0	0	g	0	
3744976	T	C	18	0	1.28	false positive		t	0	0	0	0	0	0	0	0	0	0	0	
3745057	G	A	23	0	1.09	false positive		0	0	0	0	0	0	0	0	0	0	0	0	
4232217	T	C	0	2	1.08	false positive		0	T	t	t	t	T	c	0	t	0	0	0	
4296702	T	G	0	1	1.02	false positive		G	0	0	0	0	0	0	0	0	0	0	0	

Position	**EDI003.A call**	Reference call	Uncovered positions in 50 bp	Heterozygous SNPs in 50 bp	Mean coverage ratio LS/HS	Classification	Comment	AE1175	DIT003.B	EDI001.A	EDI004.A	LSD001.A	LSD023.A	LVC_merged	PET004.A	UNT003.A	UNT004.A	VAL001.B	WAG001.A	Homoplasy
114434	G	A	0	1	2.17	false positive		A	A	A	A	a	A	a	A	A	a	A	0	
224431	T	C	0	1	1.37	false positive		C	C	N	C	C	T	N	C	C	C	C	T	
233897	C	T	0	1	1.34	false positive		T	T	N	T	t	C	T	T	T	T	t	C	
1444672	G	A	0	0	1.20	false positive?	true positive in EDI004.A, potentially shared ancestral	g	0	G	G	g	G	g	N	g	g	0	0	
1820043	C	T	0	0	1.25	false positive		T	T	T	T	T	T	t	T	T	T	T	N	
2801707	A	G	0	0	1.00	true positive?	false positive in EDI001.A	G	G	A	N	G	G	G	G	G	g	G	0	
3155163	C	A	0	1	4.05	false positive		A	N	A	N	0	A	N	N	N	A	N	C	
3849574	A	C	0	1	1.77	false positive		C	C	C	C	c	C	C	C	C	C	C	C	
3896289	C	A	0	1	1.90	false positive		A	A	A	A	a	N	a	N	A	a	a	A	
4639469	G	A	0	1	2.62	false positive		A	A	A	A	N	A	N	A	A	A	A	0	

Position	**EDI004.A call**	Reference call	Uncovered positions in 50	Heterozygous SNPs in 50 bp	Mean coverage ratio LS/HS	Classification	Comment	AE1175	DIT003.B	EDI001.A	EDI003.A	LSD001.A	LSD023.A	LVC_merged	PET004.A	UNT003.A	UNT004.A	VAL001.B	WAG001.A	Homoplasy
567757	A	C	0	0	1.00	true positive	potentially shared ancestral	a	0	a	0	a	0	0	a	0	0	N	0	
1444672	G	A	0	0	1.00	true positive?	false positive in EDI003.A and EDI004.A, potentially shared ancestral	g	0	G	G	g	G	g	N	g	g	0	0	
3262779	A	C	25	0	1.01	false positive		C	0	c	c	0	a	0	0	a	0	a	0	
3480906	G	A	0	1	1.68	false positive		A	A	A	A	N	A	A	A	A	A	A	0	

Position	**LSD001.A call**	Reference call	Uncovered positions in 50 bp	Heterozygous SNPs in 50 bp	Mean coverage ratio LS/HS	Classification	Comment	AE1175	DIT003.B	EDI001.A	EDI003.A	EDI004.A	LSD023.A	LVC_merged	PET004.A	UNT003.A	UNT004.A	VAL001.B	WAG001.A	Homoplasy
187146	A	G	0	1	1.00	false positive		G	G	G	G	G	G	G	G	G	g	G	0	
463390	T	C	0	0	1.00	true positive	unique LSD001.A	C	N	C	c	C	C	C	C	C	C	C	0	
481144	T	C	0	0	1.00	true positive	shared LSD023.A, LVC	C	C	C	c	C	T	T	C	C	C	C	0	
867563	A	G	0	0	1.00	true positive	unique LSD001.A	G	G	G	g	G	G	G	G	G	G	G	0	
1490914	A	C	0	0	1.00	true positive	shared LSD023.A, LVC	C	C	C	c	N	A	A	C	C	C	C	0	
1806544	C	A	0	0	1.00	true positive	shared LSD023.A	A	A	A	A	A	c	A	A	A	A	A	0	
1957012	T	G	0	0	1.00	true positive	shared LSD023.A, LVC	G	G	G	G	G	T	t	G	G	G	G	0	
2103712	G	T	0	0	1.00	true positive	unique LSD001.A	T	T	T	T	t	T	T	T	T	T	T	0	
2272078	T	G	0	0	1.00	true positive	unique LSD001.A	G	g	G	G	G	G	G	G	G	G	G	0	
2715042	T	C	0	0	1.00	true positive	shared LSD023.A	C	C	C	C	C	T	C	C	C	C	C	0	
2737457	T	C	0	0	1.00	true positive	shared LSD023.A, LVC	C	C	C	C	C	T	T	C	C	C	C	0	
2924618	A	G	0	0	1.00	true positive	shared LSD023.A, LVC	G	G	G	G	G	A	A	G	G	G	G	0	
3017615	G	A	0	0	2.43	false positive		A	N	A	N	N	A	A	N	N	N	N	N	
3111587	T	C	0	0	1.00	true positive	unique LSD001.A	C	C	C	C	C	C	c	C	C	C	C	0	
3228793	T	A	0	0	1.00	true positive	unique LSD001.A	A	A	A	A	A	A	A	A	A	A	A	a	
3295569	T	G	0	0	1.00	true positive	unique LSD001.A	G	G	G	G	G	G	G	G	G	g	G	0	
3665838	C	T	0	0	1.00	true positive	shared LSD023.A, LVC	T	T	T	T	T	C	C	T	T	T	T	0	
3750736	A	G	2	0	1.09	true positive?	false positive in VAL, shared AE, DIT, LSD, LVC, PET, UNT, VAL	A	a	G	G	G	A	a	A	A	A	A	a	
3996401	A	C	0	0	1.00	true positive	unique LSD001.A	C	C	C	C	C	C	C	C	C	C	C	0	
4066202	A	G	0	0	1.00	true positive	unique LSD001.A	G	G	G	G	G	G	G	G	G	G	G	0	
4416487	T	G	0	0	1.00	true positive	shared LSD023.A, LVC	G	G	G	G	G	T	T	G	G	G	G	0	
4563794	A	G	0	0	1.00	true positive?	false positive in LVC, shared LSD; LVC	G	G	G	G	G	A	A	G	G	G	G	0	
4633424	A	G	0	0	1.00	true positive	shared LSD023.A, LVC	G	G	G	G	G	A	A	G	G	G	G	0	

Position	**LSD023.A call**	Reference call	Uncovered positions in 50 bp	Heterozygous SNPs in 50 bp	Mean coverage ratio LS/HS	Classification	Comment	AE1175	DIT003.B	EDI001.A	EDI003.A	EDI004.A	LSD001.A	LVC_merged	PET004.A	UNT003.A	UNT004.A	VAL001.B	WAG001.A	Homoplasy
82545	G	T	0	0	1.00	true positive	unique LSD023.A	T	t	T	t	t	0	t	T	t	t	N	t	
216655	T	C	0	0	2.38	false positive		C	c	C	C	C	C	C	C	C	C	C	T	
216673	C	T	0	0	1.73	false positive		T	T	T	T	T	T	T	T	T	T	T	C	
218890	C	T	0	1	2.93	false positive		T	T	T	T	T	T	T	T	T	T	T	0	
221608	A	G	0	2	1.28	false positive		G	G	G	G	G	G	G	G	G	G	G	A	
221638	G	A	0	0	1.21	false positive		A	A	A	N	A	A	A	A	A	A	A	G	
221690	T	G	0	0	1.44	false positive		G	G	N	N	G	G	G	N	G	G	G	T	
221705	A	G	0	0	1.66	false positive		G	G	G	G	G	G	G	G	G	G	G	N	
222442	G	A	0	1	1.54	false positive		A	A	A	N	A	A	A	A	A	A	A	G	
222625	T	G	0	0	1.51	false positive		G	G	G	N	G	G	G	N	G	G	G	N	
222670	A	C	0	0	1.41	false positive		C	C	N	N	C	N	N	N	C	C	C	A	
222672	A	G	0	0	1.40	false positive		G	G	G	N	G	G	G	N	G	G	G	N	
223252	C	T	0	1	1.47	false positive		T	T	N	T	T	T	N	N	T	T	T	C	
224431	T	C	0	0	1.36	false positive		C	C	N	T	C	C	N	C	C	C	C	T	
224434	T	C	0	0	1.34	false positive		C	C	C	N	C	C	N	C	C	C	C	N	
225009	A	T	0	1	1.15	false positive		T	T	T	T	T	T	T	t	N	t	T	N	
225080	C	A	0	2	1.29	false positive		A	A	A	N	A	A	N	A	A	A	A	C	
225083	G	A	0	2	1.33	false positive		A	N	A	N	A	A	A	A	A	A	A	N	
225458	A	T	0	0	1.95	false positive		T	T	T	N	T	T	T	t	T	N	T	N	
225479	G	T	0	0	2.17	false positive		T	T	T	T	T	T	T	t	N	t	T	G	
225752	G	A	0	0	1.25	false positive		A	A	A	N	A	A	N	A	A	A	A	G	
226189	C	T	0	0	1.37	false positive		T	T	T	T	T	T	T	T	T	T	T	C	
226794	A	C	0	0	1.05	false positive		C	C	N	N	C	C	C	N	C	C	C	A	
227010	T	G	0	1	1.74	false positive		G	G	G	G	G	G	G	N	G	G	G	T	

Position	**LSD023.A call**	Reference call	Uncovered positions in 50 bp	Heterozygous SNPs in 50 bp	Mean coverage ratio LS/HS	Classification	Comment	AE1175	DIT003.B	EDI001.A	EDI003.A	EDI004.A	LSD001.A	LVC_merged	PET004.A	UNT003.A	UNT004.A	VAL001.B	WAG001.A	Homoplasy
227176	T	C	0	1	1.20	false positive		C	C	C	C	C	C	N	C	C	C	C	T	
227221	T	C	0	0	1.10	false positive		C	C	C	C	C	C	C	C	C	C	C	T	
227437	C	T	0	1	2.17	false positive		T	T	T	T	T	T	T	T	T	T	T	C	
227757	A	G	0	0	1.28	false positive		G	G	G	N	G	G	G	G	G	G	G	N	
228727	C	T	0	1	2.03	false positive		T	T	T	T	N	T	T	T	T	T	T	N	
228733	T	C	0	1	1.86	false positive		C	C	C	N	N	C	C	C	C	C	C	T	
228773	C	A	0	0	1.14	false positive		A	A	A	N	A	A	A	A	A	A	A	N	
228816	G	T	0	0	1.01	false positive		T	T	N	N	T	T	T	N	T	T	T	G	
229302	T	C	0	0	1.65	false positive		C	C	C	C	C	c	C	C	C	c	c	N	
229306	A	G	0	0	1.59	false positive		G	G	G	G	G	G	G	G	G	G	G	N	
229632	T	C	0	2	1.70	false positive		C	C	C	C	C	C	C	C	C	C	C	C	
229719	G	T	0	0	1.27	false positive		T	T	N	N	T	T	T	T	T	T	T	G	
229730	G	A	0	0	1.25	false positive		A	A	N	N	A	A	A	N	A	A	A	G	
229874	G	A	0	2	2.33	false positive		A	A	A	N	A	A	A	A	A	A	A	G	
231527	A	G	0	1	1.29	false positive		G	G	G	g	G	G	G	G	G	G	G	a	
232124	A	T	0	0	1.13	false positive		T	T	T	N	T	T	T	T	T	T	T	A	
232802	C	T	0	1	3.45	false positive		T	T	T	T	T	t	T	t	T	0	T	T	
233897	C	T	0	0	1.18	false positive		T	T	N	C	T	t	T	T	T	T	t	C	
233971	G	C	0	1	1.45	false positive		C	C	N	N	C	C	C	C	C	C	C	N	
234724	C	T	0	0	2.91	false positive		T	T	T	t	T	t	T	T	T	T	T	C	
234727	A	G	0	0	2.96	false positive		G	G	G	G	G	g	G	G	G	G	G	A	
481144	T	C	0	0	1.00	true positive	shared LSD001.A, LVC	C	C	C	c	C	T	T	C	C	C	C	0	
503367	G	A	0	0	1.39	false positive		A	A	A	A	A	A	A	A	A	A	A	N	
712800	A	C	0	0	1.00	true positive	shared LSD001.A, LVC	C	C	C	C	C	a	A	C	C	C	C	0	

**Position**	**LSD023.A call**	Reference call	Uncovered positions in 50 bp	Heterozygous SNPs in 50 bp	Mean coverage ratio LS/HS	Classification	Comment	AE1175	DIT003.B	EDI001.A	EDI003.A	EDI004.A	LSD001.A	LVC_merged	PET004.A	UNT003.A	UNT004.A	VAL001.B	WAG001.A	Homoplasy
881946	A	G	0	0	1.00	true positive	unique LSD023.A	G	G	G	G	G	G	G	G	G	G	G	g	
996250	A	G	0	0	1.00	true positive	unique LSD023.A	G	0	G	G	G	G	0	G	G	G	G	0	
1444672	G	A	1	0	1.00	false positive		g	0	G	G	G	g	g	N	g	g	0	0	
1490914	A	C	0	0	1.00	true positive	shared LSD001.A, LVC	C	C	C	c	N	A	A	C	C	C	C	0	
1769827	G	T	0	0	1.00	true positive	shared LSD001.A, LVC	T	T	T	T	T	g	g	T	T	t	T	0	
1806674	A	G	0	0	1.00	true positive	unique LSD023.A	G	G	G	0	G	g	g	G	G	g	G	0	
1824110	T	A	0	0	1.24	false positive		A	A	A	N	A	a	a	A	A	A	A	T	
1824116	T	G	0	0	1.21	false positive		G	G	G	t	G	g	g	g	G	G	G	T	
1957012	T	G	0	0	1.00	true positive	shared LSD001.A, LVC	G	G	G	G	G	T	t	G	G	G	G	0	
2283822	T	C	0	0	1.00	true positive	unique LSD023.A	C	C	C	C	C	C	C	C	C	C	C	0	
2338880	A	T	0	0	1.00	true positive	unique LSD023.A	T	T	T	T	T	t	T	T	T	T	T	0	
2715042	T	C	0	0	1.00	true positive	shared LSD001.A	C	C	C	C	C	T	C	C	C	C	C	0	
2737457	T	C	0	0	1.00	true positive	shared LSD001.A, LVC	C	C	C	C	C	T	T	C	C	C	C	0	
2924618	A	G	0	0	1.00	true positive	shared LSD001.A, LVC	G	G	G	G	G	A	A	G	G	G	G	0	
3024601	T	G	0	0	1.00	true positive	shared LSD001.A, LVC	G	G	G	G	G	t	T	G	G	G	G	0	
3183745	C	T	0	0	1.09	false positive		T	T	T	T	T	T	T	T	T	T	T	0	
3299755	T	C	0	0	1.66	false positive		N	N	0	N	N	0	N	c	N	t	N	0	*Y. pseudotuberculosis*
3299756	C	T	0	0	1.67	false positive		N	N	0	N	N	0	N	t	N	c	N	0	0.PE2 strains, 3.ANT1a_7b, *Y. pseudotuberculosis*
3518210	A	G	0	2	5.80	false positive		G	G	G	g	G	g	g	G	G	N	G	N	*Y. pseudotuberculosis*
3657089	C	A	0	0	1.00	true positive	unique LSD023.A	A	A	A	A	A	A	A	A	A	A	A	0	
3665838	C	T	0	0	1.00	true positive	shared LSD001.A, LVC	T	T	T	T	T	C	C	T	T	T	T	0	
3683423	T	A	0	1	1.28	false positive		A	A	A	A	A	A	A	A	A	A	A	T	

Position	LSD023.A call	Reference call	Uncovered positions in 50 bp	Heterozygous SNPs in 50 bp	Mean coverage ratio LS/HS	Classification	Comment	AE1175	DIT003.B	EDI001.A	EDI003.A	EDI004.A	LSD001.A	LVC_merged	PET004.A	UNT003.A	UNT004.A	VAL001.B	WAG001.A	Homoplasy
3750736	A	G	0	0	1.00	true positive?	false positive in VAL, LSD001.A, shared AE, DIT, LSD, LVC, PET, UNT, VAL	A	a	G	G	G	A	a	A	A	A	A	a	
3764538	G	A	0	1	1.80	false positive		A	A	A	A	A	A	N	A	A	A	A	N	
3890928	G	C	0	2	1.24	false positive		N	G	N	c	c	g	N	N	g	N	G	0	
3956046	G	A	0	0	1.55	false positive		N	A	N	N	N	N	N	A	A	N	N	N	
4098067	G	A	0	0	3.19	false positive		A	A	A	N	N	A	N	N	N	N	N	G	
4200456	A	C	0	1	3.70	false positive		C	C	C	N	C	C	N	C	N	N	C	N	
4201407	T	C	0	1	2.66	false positive		C	c	C	C	C	c	c	C	C	c	C	T	
4201449	G	A	0	1	1.63	false positive		A	A	N	N	A	A	A	N	A	A	A	G	
4205567	T	A	0	0	1.24	false positive		A	A	A	A	N	A	N	A	A	A	a	T	
4205600	G	A	0	0	1.18	false positive		A	A	A	A	A	A	A	A	A	A	A	N	
4232217	T	C	17	0	1.00	false positive		0	T	T	t	t	t	c	0	t	0	0	0	2.MED1_1045, 2.MED1_173
4416487	T	G	0	0	1.00	true positive	shared LSD001.A, LVC	G	G	G	G	G	T	T	G	G	G	G	0	
4417092	T	G	0	1	1.00	false positive		N	N	N	N	N	N	T	N	N	N	N	0	
4563794	A	G	0	0	1.00	true positive?	false positive in LVC, shared LSD, LVC	G	G	G	G	G	A	A	G	G	G	G	0	
4578141	A	G	0	2	1.48	false positive		G	G	G	G	G	g	G	G	G	G	G	A	
4633424	A	G	0	0	1.00	true positive	shared LSD001.A, LVC	G	G	G	G	G	A	A	G	G	G	G	0	
4647626	T	C	0	2	1.39	false positive		C	N	C	N	C	C	C	C	C	C	C	N	

Position	LVC_merged call	Reference call	Uncovered positions in 50 bp	Heterozygous SNPs in 50 bp	Mean coverage ratio LS/HS	Classification	Comment	AE1175	DIT003.B	EDI001.A	EDI003.A	EDI004.A	LSD001.A	LSD023.A	PET004.A	UNT003.A	UNT004.A	VAL001.B	WAG001.A	Homoplasy
218506	A	C	0	1	3.32	false positive		C	C	C	C	C	C	C	C	C	C	C	0	
481144	T	C	0	0	1.00	true positive	shared LSD, LVC	C	C	C	N	C	T	T	C	C	C	C	0	
712800	A	C	0	0	1.00	true positive	shared LSD, LVC	C	C	C	C	C	a	A	C	C	C	C	0	
1490914	A	C	0	0	1.00	true positive	shared LSD, LVC	C	C	C	N	N	A	A	C	C	C	C	0	
2515458	T	C	0	0	1.00	true positive	unique LVC	C	C	C	C	C	C	C	C	C	C	C	0	
2546208	G	A	10	0	1.27	false positive		A	A	A	A	A	0	N	A	A	A	A	0	
2671168	T	C	0	0	1.21	false positive		C	C	C	C	C	t	t	C	0	C	C	0	
2737457	T	C	0	0	1.00	true positive	shared LSD, LVC	C	C	C	C	C	T	T	C	C	C	C	0	

Position	LVC_merged call	Reference call	Uncovered positions in 50 bp	Heterozygous SNPs in 50 bp	Mean coverage ratio LS/HS	Classification	Comment	AE1175	DIT003.B	EDI001.A	EDI003.A	EDI004.A	LSD001.A	LSD023.A	PET004.A	UNT003.A	UNT004.A	VAL001.B	WAG001.A	Homoplasy
2924618	A	G	0	0	1.00	true positive	shared LSD, LVC	G	G	G	G	G	A	A	G	G	G	G	0	
3024601	T	G	0	0	1.00	true positive	shared LSD, LVC	G	G	G	G	G	t	T	G	G	G	G	0	
3393110	G	A	0	0	3.73	false positive		A	A	A	N	N	A	A	a	N	0	A	g	
3665838	C	T	0	0	1.00	true positive	shared LSD, LVC	T	T	T	T	T	C	C	T	T	T	T	0	
4416487	T	G	0	0	1.00	true positive	shared LSD, LVC	G	G	G	G	G	T	T	G	G	G	G	0	
4417092	T	G	0	1	1.00	false positive		N	N	N	N	N	N	T	N	N	N	N	0	
4563794	A	G	0	0	1.02	false positive?	true positive in LSD001.A, LSD023.A, shared LSD, LVC	G	G	G	G	G	A	A	G	G	G	G	0	
4633424	A	G	0	0	1.00	true positive	shared LSD, LVC	G	G	G	G	G	A	A	G	G	G	G	0	

Position	**PET004.A call**	Reference call	Uncovered positions in 50 bp	Heterozygous SNPs in 50 bp	Mean coverage ratio LS/HS	Classification	Comment	AE1175	DIT003.B	EDI001.A	EDI003.A	EDI004.A	LSD001.A	LSD023.A	LVC_merged	UNT003.A	UNT004.A	VAL001.B	WAG001.A	Homoplasy
514923	T	C	0	1	1.66	false positive		C	C	C	C	C	C	C	C	C	C	C	c	
1573356	C	T	0	1	2.90	false positive		T	T	T	T	T	N	N	N	T	T	N	N	
1791420	G	A	0	0	1.00	true positive	unique PET	A	a	A	a	A	a	0	0	A	A	A	0	
3750736	A	G	0	0	1.00	true positive?	false positive in VAL, LSD001.A, shared AE, DIT, LSD, LVC, PET, UNT, VAL	A	a	G	G	G	A	A	a	A	A	A	A	
3848782	A	T	0	0	3.27	false positive		N	T	T	T	T	T	T	N	T	N	N	T	
3921330	T	C	0	0	1.00	true positive	unique PET	C	C	C	C	C	C	C	C	C	C	C	0	*Y. pseudotuberculosis*
4198065	G	A	0	0	1.53	false positive		A	A	A	A	A	A	A	A	A	A	A	0	
4198659	C	A	0	2	2.60	false positive		A	N	A	A	N	N	A	A	A	A	A	N	
4210722	T	A	0	1	2.95	false positive		A	A	A	A	A	A	N	N	A	A	A	0	
4412624	G	A	0	0	1.00	true positive?	false positive in UNT003.A, true positive in AE, potentially shared AE, DIT, LSD, LVC, PET, UNT, VAL	G	0	A	0	a	g	g	0	G	g	g	0	
4644248	T	C	0	0	1.45	false positive		C	C	C	N	C	c	C	C	C	C	C	c	

Position	**UNT003.A call**	Reference call	Uncovered positions in 50 bp	Heterozygous SNPs in 50 bp	Mean coverage ratio LS/HS	Classification	Comment	AE1175	DIT003.B	EDI001.A	EDI003.A	EDI004.A	LSD001.A	LSD023.A	LVC_merged	PET004.A	UNT004.A	VAL001.B	WAG001.A	Homoplasy
549767	C	T	0	0	1.11	false positive		C	C	T	T	T	c	c	c	c	C	c	0	
1480098	T	G	0	0	1.00	true positive	potentially shared AE, DIT, homoplastic in LSD?	0	t	G	G	G	t	t	g	g	0	0	0	
3179828	A	C	0	0	1.00	true positive	shared AE, DIT, UNT	A	A	C	C	C	c	C	C	C	A	C	0	
3750736	A	G	0	0	1.00	true positive?	false positive in VAL, LSD001.A, shared AE, DIT, LSD, LVC, PET, UNT, VAL	A	a	G	G	G	A	A	a	A	A	A	a	
4412624	G	A	0	0	1.27	true positive?	true positive in AE, PET, potentially shared AE, DIT, LSD, LVC, PET, UNT, VAL	G	0	A	0	a	g	g	0	G	g	g	0	

Position	**UNT004.A call**	Reference call	Uncovered positions in 50	Heterozygous SNPs in 50 bp	Mean coverage ratio LS/HS	Classification	Comment	AE1175	DIT003.B	EDI001.A	EDI003.A	EDI004.A	LSD001.A	LSD023.A	LVC_merged	PET004.A	UNT003.A	VAL001.B	WAG001.A	Homoplasy
225458	G	T	0	1	1.76	false positive		T	T	T	N	T	T	A	T	t	T	t	N	
231350	C	T	24	1	1.58	false positive		T	T	T	T	T	T	T	N	N	T	T	g	
3155190	C	T	0	1	1.91	false positive		T	T	T	T	T	t	T	T	T	T	N	0	
3750736	A	G	0	0	1.00	true positive?	false positive in VAL, LSD001.A, shared AE, DIT, LSD, LVC, PET, UNT, VAL	A	a	G	G	G	A	A	a	A	A	A	a	
3685814	A	G	0	0	1.59	false positive		G	g	G	0	G	0	N	G	g	g	G	G	
3685844	A	G	5	0	1.75	false positive		G	g	G	g	g	g	g	G	G	g	g	G	
4199133	A	C	0	2	2.43	false positive		C	C	C	C	C	C	C	C	C	C	C	N	
4201886	G	A	0	1	1.75	false positive		G	G	G	G	G	G	G	G	G	G	G	G	

**Position**	**VAL001.B call**	Reference call	Uncovered positions in 50 bp	Heterozygous SNPs in 50 bp	Mean coverage ratio LS/HS	Classification	Comment	AE1175	DIT003.B	EDI001.A	EDI003.A	EDI004.A	LSD001.A	LSD023.A	LVC_merged	PET004.A	UNT003.A	UNT004.A	WAG001.A	Homoplasy
718827	C	T	21	0	1.32	false positive		c	c	C	0	c	0	c	c	c	c	c	0	Branch 2
1361842	T	C	0	0	1.00	true positive	unique VAL	C	C	C	C	C	C	C	C	C	C	C	0	
2041294	G	T	22	0	1.00	false positive		T	T	T	t	T	t	t	0	T	t	t	0	
2253178	A	C	0	0	1.00	true positive	unique VAL	C	C	C	C	C	C	C	C	C	C	C	0	
2303798	C	T	16	0	1.92	false positive		T	N	T	N	N	N	N	N	N	N	N	N	
2732804	G	C	0	0	1.12	false positive		C	C	C	C	C	C	C	C	C	c	c	0	
2911604	G	A	0	0	1.00	true positive	unique VAL	A	A	A	A	A	A	A	A	A	A	A	0	
3623563	A	G	0	1	1.07	false positive		N	N	N	N	N	N	N	N	0	N	a	0	
3750736	A	G	5	0	1.00	true positive?	false positive in VAL, LSD001.A, shared AE, DIT, LSD, LVC, PET, UNT, VAL	A	a	G	G	G	A	A	a	A	A	A	a	
3890928	G	C	3	0	1.17	false positive		N	G	N	c	c	g	G	N	N	g	A	0	
4256445	G	A	0	0	1.10	false positive		0	A	A	A	A	A	A	0	A	A	N	0	
4635136	A	C	0	1	1.17	false positive		N	C	N	N	N	c	N	N	c	a	G	0	
4648185	A	G	0	0	1.03	false positive		G	G	G	G	G	G	G	G	G	G	c	G	

Table S9: Table with all non-shared plasmidal SNPs that were called in the investigated First Pandemic genomes, sorted by genome (second cell in header). ‘Position’ refers to the position on the plasmid of the reference genome CO92, followed by the respective SNP call and the reference call. The classification as potential false or true positive is based on the preceding parameters regarding a 50 bp window surrounding the SNP: only SNPs with no uncovered position, no heterozygous SNP and an equal mean coverage with high and low stringent mapping (ratio of 1.00) are classified as true positive SNPs (highlighted in green). If a SNP is ambiguous due to conflicting classification in different samples, this is indicated with a question mark highlighted in orange. The ‘comment’ column with additional classification is followed by the respective calls in the other First Pandemic genomes (0=not covered, N=heterozygous, lower case letters=less than 3 reads coverage).

**Plasmid**	**Position**	**AE1175 call**	Reference call	Uncovered positions in 50 bp	Heterozygous SNPs in 50 bp	Mean coverage ratio LS/HS	Classification	Comment	DIT003.B	EDI001.A	EDI003.A	EDI004.A	LSD001.A	LSD023.A	LVC_merged	PET004.A	UNT003.A	UNT004.A	VAL001.B	WAG001.A
pCD1	18753	C	G	0	2	1.12	false positive		c	N	0	0	N	0	0	c	0	0	0	0
pCD1	29959	G	A	0	0	1.02	false positive?	Feldman et al. 2016; false positive in DIT, true positive in LSD, LVC, PET, UNT, VAL	G	A	A	A	G	G	G	G	G	G	G	0

**Plasmid**	**Position**	**DIT003.B call**	Reference call	Uncovered positions in 50	Heterozygous SNPs in 50 bp	Mean coverage ratio LS/HS	Classification	Comment	AE1175	EDI001.A	EDI003.A	EDI004.A	LSD001.A	LSD023.A	LVC_merged	PET004.A	UNT003.A	UNT004.A	VAL001.B	WAG001.A
pCD1	29959	G	A	0	0	1.05	false positive?	false positive in AE, true positive in LSD, LVC, PET, UNT, VAL	G	A	A	A	G	G	G	G	G	G	G	0

**Plasmid**	**Position**	**LSD001.A call**	Reference call	Uncovered positions in 50 bp	Heterozygous SNPs in 50 bp	Mean coverage ratio LS/HS	Classification	Comment	AE1175	DIT003.B	EDI001.A	EDI003.A	EDI004.A	LSD023.A	LVC_merged	PET004.A	UNT003.A	UNT004.A	VAL001.B	WAG001.A
pCD1	21278	A	G	0	0	1.00	true positive	shared LSD, LVC	G	G	G	G	G	A	A	G	G	G	G	0
pCD1	29959	G	A	0	0	1.00	true positive	false positive in AE, DIT, true positive in LSD, LVC, PET, UNT, VAL	G	G	A	A	A	G	G	G	G	G	G	0
pCD1	46912	G	A	6	0	1.43	false positive		N	g	N	a	N	0	g	N	0	a	0	0
pCD1	51106	A	G	0	0	1.00	true positive	unique LSD001.A	G	G	G	G	G	G	G	G	G	G	G	0

**Plasmid**	**Position**	**LSD023.A call**	Reference call	Uncovered positions in 50 bp	Heterozygous SNPs in 50 bp	Mean coverage ratio LS/HS	Classification	Comment	AE1175	DIT003.B	EDI001.A	EDI003.A	EDI004.A	LSD001.A	LVC_merged	PET004.A	UNT003.A	UNT004.A	VAL001.B	WAG001.A
pCD1	21278	G	A	0	0	1.00	true positive	shared LSD, LVC	G	G	G	G	G	A	A	G	G	G	G	0
pCD1	29959	G	A	0	0	1.00	true positive	false positive in AE, DIT, true positive in LSD, LVC, PET, UNT, VAL	G	G	A	A	A	G	G	G	G	G	G	0
pMT1	81057	T	A	0	2	1.00	false positive		N	N	N	N	N	N	N	N	N	N	N	t
pPCP1	8528	C	G	0	0	1.00	false positive*	*see screenshot Fig. S10	0	T	N	t	t	0	N	T	T	t	T	0
pPCP1	8529	G	T	0	0	1.00	false positive*	*see screenshot Fig. S10, false positive in LVC	0	T	N	t	t	g	G	T	T	t	T	0

**Plasmid**	**Position**	**LVC_merged call**	Reference call	Uncovered positions in 50 bp	Heterozygous SNPs in 50 bp	Mean coverage ratio LS/HS	Classification	Comment	AE1175	DIT003.B	EDI001.A	EDI003.A	EDI004.A	LSD001.A	LSD023.A	PET004.A	UNT003.A	UNT004.A	VAL001.B	WAG001.A
pCD1	21278	G	A	0	0	1.00	true positive	shared LSD, LVC	G	G	G	G	G	A	A	G	G	G	G	0
pCD1	29959	G	A	0	0	1.00	true positive	false positive in AE, DIT, true positive in LSD, LVC, PET, UNT, VAL	G	G	A	A	A	G	G	G	G	G	G	0
pPCP1	8529	G	T	0	1	1.00	false positive		0	T	N	t	t	g	G	T	T	t	T	0

**Plasmid**	**Position**	**PET004.A call**	Reference call	Uncovered positions in 50	Heterozygous SNPs in 50 bp	Mean coverage ratio LS/HS	Classification	Comment	AE1175	DIT003.B	EDI001.A	EDI003.A	EDI004.A	LSD001.A	LSD023.A	LVC_merged	UNT003.A	UNT004.A	VAL001.B	WAG001.A
pCD1	29959	G	A	0	0	1.00	true positive	false positive in AE, DIT, true positive in LSD, LVC, PET, UNT, VAL	G	G	A	A	G	G	G	G	G	G	G	0

**Plasmid**	**Position**	**UNT003.A call**	Reference call	Uncovered positions in 50	Heterozygous SNPs in 50 bp	Mean coverage ratio LS/HS	Classification	Comment	AE1175	DIT003.B	EDI001.A	EDI003.A	EDI004.A	LSD001.A	LSD023.A	LVC_merged	PET004.A	UNT004.A	VAL001.B	WAG001.A
pCD1	29959	G	A	0	0	1.00	true positive	false positive in AE, DIT, true positive in LSD, LVC, PET, UNT, VAL	G	G	A	A	A	G	G	G	G	G	G	0
pMT1	59223	A	T	0	1	1.39	false positive		N	N	T	T	N	N	T	N	N	a	T	0

**Plasmid**	**Position**	**UNT004.A call**	Reference call	Uncovered positions in 50	Heterozygous SNPs in 50 bp	Mean coverage ratio LS/HS	Classification	Comment	AE1175	DIT003.B	EDI001.A	EDI003.A	EDI004.A	LSD001.A	LSD023.A	LVC_merged	PET004.A	UNT003.A	VAL001.B	WAG001.A
pCD1	29959	G	A	0	0	1.00	true positive	false positive in AE, DIT, true positive in LSD, LVC, PET, UNT, VAL	G	G	A	A	A	G	G	G	G	G	G	0
pMT1	81058	G	A	0	3	1.14	false positive		N	G	N	N	a	N	N	g	N	N	G	0

**Plasmid**	**Position**	**VAL001.B call**	Reference call	Uncovered positions in 50	Heterozygous SNPs in 50 bp	Mean coverage ratio LS/HS	Classification	Comment	AE1175	DIT003.B	EDI001.A	EDI003.A	EDI004.A	LSD001.A	LSD023.A	LVC_merged	PET004.A	UNT003.A	UNT004.A	WAG001.A
pCD1	29959	G	A	0	0	1.00	true positive	false positive in AE, DIT, true positive in LSD, LVC, PET, UNT, VAL	G	G	A	A	A	G	G	G	G	G	G	0
pMT1	59982	C	A	0	0	1.00	true positive	unique VAL	T	T	T	T	a	T	T	T	T	T	0	0

Table S10: True SNPs identified in the genomes of Edix Hill (EDI), Dittenheim (DIT), Saint-Doulchard (LSD), Lunel-Viel (LVC), Petting (PET), Unterthürheim (UNT) and Valencia (VAL). For all identified unique SNPs including potential false positives, see SI Appendix, Table S5 and S6. *This SNP appears in only one or two reads (below our SNP calling threshold of min. 3 reads).

Chromosome										
Position	Genome	Ref	SNP	Tree	SNP type	Codon change	AA change	Gene ID	Gene name	Function
3750736	AE, DIT, LSD001.A, LSD023.A, LVC*, PET, UNT, VAL, WAG*	G	A	yes	Intergenic	-	-	-	-	-
3179828	AE, DIT, UNT	C	A	yes	Synonymous	tcC/tcA	S/S	YPO2847	*yegM*	Multidrug efflux system subunit MdtA
1444672	AE, EDI, LSD001.A*, LSD023.A, LVC*, UNT*	A	G	no	Intergenic	-	-	-	-	-
3755861	AE, EDI, LSD023.A*, LVC*, PET*, UNT*	C	T	no	Intergenic	-	-	-	-	-
4412624	AE, LSD001.A*, LSD023.A*, PET, UNT, VAL*	A	G	no	Intergenic	-	-	-	-	-
567757	AE*, EDI, LSD001.A*, PET*	C	A	no	Non-synonymous	Ctt/Att	L/I	YPO0523	-	Hypothetical protein
1480098	DIT*, LSD001.A*, LSD023.A*, UNT	G	T	no	Intergenic	-	-	-	-	-
481144	LSD001.A, LSD023.A, LVC	C	T	yes	Synonymous	Ctg/Ttg	L/L	YPO0457	creA	Hypothetical protein
712800	LSD001.A*, LSD023.A, LVC	C	A	yes	Synonymous	cgC/cgA	R/R	YPO0653	glnE	Bifunctional glutamine-synthetase adenylyltransferase/deadenyltransferase
1490914	LSD001.A, LSD023.A, LVC	C	A	yes	Upstream: 63 bases	-	-	YPO1324	-	Undecaprenyl pyrophosphate phosphatase

Chromosome										
Position	Genome	Ref	SNP	Tree	SNP type	Codon change	AA change	Gene ID	Gene name	Function
1769827	LSD001.A, LSD023.A*, LVC*	T	G	yes	Non-synonymous	Acc/Ccc	T/P	YPO1554	-	Pseudogene (sugar transport ATP-binding protei)
1957012	LSD001.A, LSD023.A, LVC*	G	T	yes	Non-synonymous	ttG/ttT	L/F	YPO1713	ogl	Oligogalacturonate lyase
2737457	LSD001.A, LSD023.A, LVC	C	T	yes	Non-synonymous	gCg/gTg	A/V	YPO2437	marC	multiple drug resistance protein MarC
2924618	LSD001.A, LSD023.A, LVC	G	A	yes	Synonymous	gcC/gcT	A/A	YPO2603	rodA	Cell wall shape-determining protein
3024601	LSD001.A*, LSD023.A, LVC	G	T	yes	Non-synonymous	atG/atT	M/I	YPO2696	phrB	3',5'-Cyclic-nucleotide phosphodiesterase
3665838	LSD001.A, LSD023.A, LVC	T	C	yes	Non-synonymous	aAa/aGa	K/R	YPO3285	tyrA	Bifunctional chorismate mutase/prephenate dehydrogenase
4416487	LSD001.A, LSD023.A, LVC	G	T	yes	Non-synonymous	atG/atT	M/I	YPO3929	ppc	Phosphoenolpyruvate carboxylase
4563794	LSD001.A, LSD023.A, LVC	G	A	yes	Intergenic	-	-	-	-	-
4633424	LSD001.A, LSD023.A, LVC	G	A	yes	Non-synonymous	aCg/aTg	T/M	YPO4112	-	Hypothetical protein
1806544	LSD001.A, LSD023.A*	A	C	yes	Non-synonymous	ttT/ttG	F/L	YPO1582	lacY	Galactoside permease
2715042	LSD001.A, LSD023.A	C	T	yes	Synonymous	caG/caA	Q/Q	YPO2414	lplA	Lipoate-protein ligase A
2801707	EDI	G	A	yes	Synonymous	acC/acT	T/T	YPO2493	-	Dioxygenase subunit alpha
463390	LSD001.A	C	T	yes	Synonymous	gcC/gcT	A/A	YPO0442	serB	Phosphoserine phosphatase
867563	LSD001.A	G	A	yes	Intergenic	-	-	-	-	-
2103712	LSD001.A	T	G	yes	Non-synonymous	Ttt/Gtt	F/V	YPO1856	-	Hypothetical protein
2272078	LSD001.A	G	T	yes	Non-synonymous	Caa/Aaa	Q/K	YPO1999	-	Decarboxylase
3111587	LSD001.A	C	T	yes	Non-synonymous	Ccc/Tcc	P/S	YPO2777	hisP	histidine/lysine/arginine/ornithine transporter subunit
3228793	LSD001.A	A	T	yes	Non-synonymous	ttA/ttT	L/F	YPO2887	yapB	Pseudogene

Chromosome										
Position	Genome	Ref	SNP	Tree	SNP type	Codon change	AA change	Gene ID	Gene name	Function
3295569	LSD001.A	G	T	yes	Upstream: 38 bases	-	-	YPO2949	-	Hypothetical protein
3996401	LSD001.A	C	A	yes	Intergenic	-	-	-	-	-
4066202	LSD001.A	G	A	yes	Downstream: 98 bases	-	-	-	-	-
82545	LSD023.A	T	G	yes	Non-synonymous	Atc/Ct	I/L	YPO0073	cpxA	Two-component sensor kinase
881946	LSD023.A	G	A	yes	Synonymous	agG/agA	R/R	YPO0801		Hypothetical protein
996250	LSD023.A	G	A	yes	Intergenic	-	-	-	-	-
1806674	LSD023.A	G	A	yes	Non-synonymous	gCc/gTc	A/V	YPO1582	lacY	Galactoside permease
2283822	LSD023.A	C	T	yes	Synonymous	ctG/ctA	L/L	YPO2011	pth	Peptidyl-tRNA hydrolase
2338880	LSD023.A	T	A	yes	Non-synonymous	Agt/Tgt	S/C	YPO2060	znuC	High-affinity zinc transporter ATPase
3657089	LSD023.A	A	C	yes	Synonymous	cgT/cgG	R/R	YPO3275	clpB	Protein disaggregation chaperone
2515458	LVC	C	T	yes	Non-synonymous	cCg/cTg	P/L	YPO2238	-	Hypothetical protein
1791420	PET	A	G	yes	Intergenic	-	-	-	-	-
3921330	PET	C	T	yes	Non-synonymous	cGc/cAc	R/H	YPO3510	-	Hypothetical protein
1361842	VAL	C	T	yes	Non-synonymous	gGt/gAt	G/D	YPO1209	tyrP	Tyrosine specific transport protein
2253178	VAL	C	A	yes	Non-synonymous	Cgc/Agc	R/S	YPO1984	-	Hypothetical protein
2911604	VAL	A	G	yes	Non-synonymous	Agt/Ggt	S/G	YPO2588	-	ABC transport protein
Plasmid pCD1										
Position	Genome	Ref	SNP	SNP type		Codon change	AA change	Gene ID	Gene name	Function
29959	AE, DIT, LSD, LVC, PET, UNT, VAL	A	G	Non-synonymous		aAc/aGc	N/S	YPCD1.41	yscO	Putative type III secretion protein
21278	LSD, LVC	G	A	Non-synonymous		gCa/gTa	A/V	YPCD1.29c	yopB	Yersinia outer membrane protein
51106	LSD001.A	G	A	Intergenic		-	-	-	-	-
Plasmid pMT1										
Position	Genome	Ref	SNP	SNP type		Codon change	AA change	Gene ID	Gene name	Function
59982	VAL	A	C	Non-synonymous		aTc/aGc	I/C	YPMT1.59C	-	Putative DNA-binding protein

Table S11: Table with all shared SNPs that were called in the First Pandemic genomes, giving the results of the SNP evaluation for each genome separately. Position is referring to the position in the reference genome CO92, followed by the reference call. For each genome, the call at the SNP position is given (0=not covered, N=heterozygous, lower case letters=less than 3 reads coverage). The classification as potential false or true positive is based on the preceding parameters regarding a 50 bp window surrounding the SNP: Only SNPs with no uncovered position, no heterozygous SNP and an equal mean coverage with high and low stringent mapping (ratio of 1.00) are classified as a candidates for true positive SNPs. SNPs that are supported by less than half of the genomes (<6, last column) are excluded as inconclusive (highlighted in orange). Although the WAG001.A sample was not included in this analysis due to low coverage, all positions were checked for a rough phylogenetic classification. Similarly, the pre-Justinianic DA101 sample was checked for all positions as well.

Position	Reference call	AE1175				DIT003.B				EDI001.A				EDI003.A			
		SNP Call	Uncovered positions in 50 bp	Heterozygous SNPs in 50 bp	Mean coverage ratio LS/HS	SNP Call	Uncovered positions in 50 bp	Heterozygous SNPs in 50 bp	Mean coverage ratio LS/HS	SNP Call	Uncovered positions in 50 bp	Heterozygous SNPs in 50 bp	Mean coverage ratio LS/HS	SNP Call	Uncovered positions in 50 bp	Heterozygous SNPs in 50 bp	Mean coverage ratio LS/HS
86824	A	G	0	0	1.00	0	43	0	1.00	G	0	0	1.00	g	12	0	1.00
189912	A	G	0	0	1.03	G	0	0	1.00	G	0	0	1.00	G	0	0	1.00
260148	C	T	0	0	1.00	T	0	0	1.00	T	0	0	1.02	T	0	0	1.00
271114	C	A	0	0	1.00	A	0	1	1.26	A	0	0	1.01	A	0	0	1.00
485976	C	T	0	0	1.00	T	0	0	1.00	T	0	0	1.02	T	9	0	1.00
557841	C	T	0	0	1.00	T	0	0	1.03	T	0	0	1.02	0	11	0	1.00
727741	G	A	0	0	1.00	A	0	0	1.00	A	0	0	1.00	a	0	0	1.00
779365	C	T	0	0	1.00	T	0	0	1.00	T	0	0	1.00	T	0	0	1.00
898980	A	T	0	0	1.00	t	0	0	1.00	T	0	0	1.00	t	0	0	1.00
1067966	C	A	0	0	1.00	A	0	0	1.00	A	0	0	1.01	A	0	0	1.00
1211729	A	C	0	0	1.00	C	0	0	1.00	C	0	0	1.01	C	0	0	1.00
1296743	C	T	0	0	1.00	T	0	0	1.08	T	0	0	1.00	T	0	0	1.00
1387701	C	T	0	0	1.00	t	9	0	1.00	T	0	0	1.00	t	0	0	1.00
1387756	A	G	0	0	1.00	0	42	0	1.00	G	0	0	1.00	G	0	0	1.00
1413031	C	A	0	0	1.00	A	0	0	1.00	A	0	0	1.01	A	0	1	1.00
1434752	C	A	0	0	1.03	a	0	0	1.00	A	0	0	1.00	A	0	0	1.00
1489055	C	T	0	0	1.00	T	0	0	1.00	T	0	0	1.00	T	0	0	1.00
1530658	C	A	0	0	1.00	A	0	0	1.00	A	0	0	1.00	a	0	0	1.00
1609461	T	C	0	0	1.00	C	0	0	1.10	C	0	0	1.01	0	27	0	1.00
1754708	C	T	0	0	1.00	T	0	0	1.00	T	0	0	1.04	t	0	0	1.33
1868678	G	T	0	0	1.00	T	0	0	1.15	T	0	0	1.04	0	7	0	1.00
1956162	T	C	0	0	1.00	0	41	0	1.00	C	0	0	1.00	c	0	0	1.00
2092152	C	T	0	0	1.00	t	0	0	1.00	T	0	0	1.00	t	9	0	1.00
2097520	G	T	0	0	1.00	T	0	0	1.00	T	0	0	1.00	T	0	0	1.00
2352174	T	G	0	0	1.00	G	0	0	1.00	G	0	0	1.04	G	0	0	1.00

Position	EDI004.A				LSD001.A				LSD023.A				LVC_merged			
	SNP Call	Uncovered positions in 50 bp	Heterozygous SNPs in 50 bp	Mean coverage ratio LS/HS	SNP Call	Uncovered positions in 50 bp	Heterozygous SNPs in 50 bp	Mean coverage ratio LS/HS	SNP Call	Uncovered positions in 50 bp	Heterozygous SNPs in 50 bp	Mean coverage ratio LS/HS	SNP Call	Uncovered positions in 50 bp	Heterozygous SNPs in 50 bp	Mean coverage ratio LS/HS
86824	G	0	0	1.00	g	8	0	1.00	g	11	0	1.00	0	48	0	1.00
189912	G	0	0	1.00	G	0	0	1.00	G	0	0	1.00	G	0	0	1.00
260148	T	0	0	1.00	0	20	0	1.00	T	0	0	1.00	t	6	0	1.29
271114	A	0	0	1.00	A	0	0	1.00	A	0	0	1.00	a	0	0	1.69
485976	T	0	0	1.00	T	0	0	1.00	T	0	0	1.00	T	0	0	1.00
557841	T	0	0	1.00	t	0	0	1.00	T	0	0	1.00	T	0	0	1.06
727741	A	0	0	1.00	0	25	0	1.15	a	0	0	1.00	A	0	0	1.00
779365	0	16	0	1.00	t	0	0	1.00	T	0	0	1.00	T	0	0	1.00
898980	t	0	0	1.00	T	0	0	1.00	t	0	0	1.00	T	0	0	1.00
1067966	A	0	0	1.00	A	0	0	1.00	A	0	0	1.00	A	0	0	1.00
1211729	C	0	0	1.05	C	0	0	1.00	C	0	0	1.00	C	0	0	1.00
1296743	T	0	0	1.00	T	0	0	1.00	T	0	0	1.00	T	0	0	1.04
1387701	T	0	0	1.00	T	7	0	1.00	T	0	0	1.00	0	31	0	1.00
1387756	0	4	0	1.00	0	50	0	NA	0	42	0	1.00	g	20	0	1.00
1413031	A	0	0	1.00	A	0	0	1.00	A	0	0	1.00	a	2	0	1.00
1434752	A	0	0	1.00	A	0	0	1.19	A	0	0	1.00	A	0	0	1.00
1489055	T	0	0	1.00	T	0	0	1.05	T	0	0	1.00	N	0	0	1.00
1530658	A	0	0	1.00	a	0	0	1.00	a	0	0	1.00	a	11	0	1.00
1609461	C	0	0	1.00	C	0	0	1.00	C	0	0	1.00	C	0	0	1.00
1754708	T	0	0	1.00	t	0	0	1.00	t	0	0	1.00	T	0	0	1.00
1868678	T	0	0	1.00	t	1	0	1.00	T	0	0	1.00	T	0	0	1.00
1956162	C	0	0	1.00	c	12	0	1.00	c	23	0	1.00	0	30	0	1.00
2092152	T	0	0	1.00	0	50	0	NA	T	0	0	1.00	0	12	0	1.00
2097520	T	0	0	1.00	T	7	0	1.00	T	0	0	1.00	N	7	1	1.00
2352174	g	0	0	1.00	G	0	0	1.07	g	0	0	1.00	G	0	0	1.00

Position	PET004.A				UNT003.A				UNT004.A				VAL001.B				DA101 call	WAG001.A call	No. of genomes passing criteria
	SNP Call	Uncovered positions in 50 bp	Heterozygous SNPs in 50 bp	Mean coverage ratio LS/HS	SNP Call	Uncovered positions in 50 bp	Heterozygous SNPs in 50 bp	Mean coverage ratio LS/HS	SNP Call	Uncovered positions in 50 bp	Heterozygous SNPs in 50 bp	Mean coverage ratio LS/HS	SNP Call	Uncovered positions in 50 bp	Heterozygous SNPs in 50 bp	Mean coverage ratio LS/HS			
86824	G	0	0	1.00	g	0	0	1.00	g	0	0	1.00	0	48	0	1.00	A	0	6
189912	G	0	0	1.00	G	0	0	1.00	G	0	0	1.00	G	0	0	1.00	A	0	11
260148	t	0	0	1.00	T	0	0	1.00	T	0	0	1.00	t	0	0	1.00	T	0	9
271114	A	0	0	1.00	A	0	0	1.00	A	0	0	1.23	A	0	0	1.00	C	a	8
485976	t	1	0	1.00	T	0	0	1.00	T	0	0	1.00	T	0	0	1.00	C	0	9
557841	t	0	0	1.00	T	0	0	1.00	T	0	0	1.00	T	0	0	1.00	C	0	8
727741	a	0	0	1.00	A	0	0	1.00	a	0	0	1.28	A	0	0	1.00	G	a	10
779365	T	0	0	1.00	T	0	0	1.00	T	0	0	1.00	T	0	0	1.00	C	0	11
898980	t	0	0	1.00	T	0	0	1.12	t	0	0	1.00	t	0	0	1.00	A	0	11
1067966	A	0	0	1.00	A	0	0	1.00	A	0	0	1.09	A	0	0	1.00	C	0	10
1211729	C	0	0	1.08	C	0	0	1.00	C	0	0	1.00	C	0	0	1.04	A	0	8
1296743	T	0	0	1.00	T	0	0	1.00	T	0	0	1.06	T	0	0	1.09	C	t	8
1387701	T	0	0	1.00	t	0	0	1.00	T	0	0	1.00	T	0	0	1.00	c	0	10
1387756	0	27	0	1.00	g	11	0	1.00	G	0	0	1.00	0	35	0	1.00	A	0	4
1413031	a	5	0	1.00	a	0	0	1.00	A	0	0	1.00	a	0	0	1.00	C	0	8
1434752	A	0	0	1.00	A	0	0	1.00	A	0	0	1.00	A	0	0	1.00	C	0	10
1489055	T	0	0	1.00	T	0	0	1.00	T	0	0	1.00	T	0	0	1.00	C	t	11
1530658	a	0	0	1.00	A	0	0	1.00	a	0	0	1.00	a	0	0	1.00	C	0	11
1609461	C	0	0	1.00	C	0	0	1.00	C	0	0	1.00	C	0	0	1.00	T	0	9
1754708	T	0	0	1.00	T	0	0	1.00	0	25	0	1.00	t	0	0	1.00	C	0	9
1868678	T	0	0	1.00	T	0	0	1.00	T	0	0	1.00	T	0	0	1.00	G	0	9
1956162	C	0	0	1.00	C	0	0	1.00	C	0	0	1.00	C	0	0	1.00	T	c	9
2092152	t	15	0	1.00	T	0	0	1.00	0	9	0	1.00	t	0	0	1.00	C	0	7
2097520	T	0	0	1.00	T	0	0	1.00	t	0	0	1.00	T	0	0	1.00	G	t	10
2352174	G	0	0	1.00	G	0	0	1.00	G	0	0	1.00	G	0	0	1.00	T	0	10

Position	Reference call	AE1175				DIT003.B				EDI001.A				EDI003.A				EDI004.A				LSD001.A			
		SNP Call	Uncovered positions in 50 bp	Heterozygous SNPs in 50 bp	Mean coverage ratio LS/HS	SNP Call	Uncovered positions in 50 bp	Heterozygous SNPs in 50 bp	Mean coverage ratio LS/HS	SNP Call	Uncovered positions in 50 bp	Heterozygous SNPs in 50 bp	Mean coverage ratio LS/HS	SNP Call	Uncovered positions in 50 bp	Heterozygous SNPs in 50 bp	Mean coverage ratio LS/HS	SNP Call	Uncovered positions in 50 bp	Heterozygous SNPs in 50 bp	Mean coverage ratio LS/HS	SNP Call	Uncovered positions in 50 bp	Heterozygous SNPs in 50 bp	Mean coverage ratio LS/HS
2419529	G	A	0	0	1.00	A	0	0	1.00	A	0	0	1.00	a	0	0	1.00	A	0	0	1.00	A	0	0	1.00
2495165	C	A	0	0	1.00	0	50	0	1.00	A	0	0	1.00	0	4	0	1.00	A	0	0	1.00	0	38	0	1.00
2725715	C	T	0	0	1.00	t	14	0	1.00	T	0	0	1.03	t	0	0	1.00	T	0	0	1.00	T	0	0	1.00
2753572	A	T	0	0	1.00	T	0	0	1.00	T	0	0	1.02	T	0	0	1.00	T	0	0	1.00	T	0	0	1.00
2977542	C	A	0	0	1.00	A	0	0	1.00	A	0	0	1.00	A	0	0	1.00	A	0	0	1.00	A	0	0	1.00
3078807	C	A	0	0	1.05	A	0	0	1.03	A	0	0	1.00	A	0	0	1.00	A	0	0	1.00	A	0	0	1.00
3274298	A	T	0	0	1.00	T	0	0	1.00	T	0	0	1.00	t	25	0	1.00	T	0	0	1.00	T	0	0	1.00
3360963	A	C	0	0	1.08	C	0	0	1.27	C	0	0	1.09	0	9	0	1.00	C	0	0	1.00	C	0	0	1.16
3360984	C	T	0	0	1.06	T	0	0	1.28	T	0	0	1.11	T	6	0	1.00	T	0	0	1.00	T	0	0	1.17
3398153	G	A	0	0	1.00	A	0	0	1.05	A	0	0	1.02	a	0	0	1.00	A	0	0	1.00	A	0	0	1.00
3409414	T	C	0	0	1.00	c	6	0	1.00	C	0	0	1.00	c	0	0	1.00	C	0	0	1.00	0	37	0	1.00
3500922	T	G	0	0	1.00	G	0	0	1.00	G	0	0	1.00	G	0	0	1.05	G	0	0	1.00	N	9	0	1.00
3535148	G	T	0	0	1.00	T	0	0	1.00	T	0	0	1.01	T	0	0	1.00	T	0	0	1.00	T	0	0	1.00
3560088	G	A	0	0	1.00	A	0	0	1.00	A	0	0	1.01	A	0	0	1.00	A	0	0	1.00	A	0	0	1.00
3568597	C	T	0	0	1.00	T	0	0	1.00	T	0	0	1.03	T	0	0	1.08	T	0	0	1.00	T	0	0	1.00
3755861	C	T	0	0	1.00	0	29	0	1.00	T	0	0	1.01	t	23	0	1.00	0	23	0	1.00	0	49	0	1.00
3843195	C	A	0	0	1.00	A	0	0	1.00	A	0	0	1.02	a	24	0	1.00	A	0	0	1.00	A	0	0	1.00
3892488	C	T	0	0	1.00	T	0	0	1.00	T	0	0	1.02	T	0	0	1.00	T	0	0	1.00	T	0	0	1.00
4066494	C	T	0	0	1.00	t	17	0	1.37	T	0	0	1.00	T	0	0	1.30	T	0	0	1.00	0	38	0	1.00
4307755	G	A	0	0	1.00	A	0	0	1.00	A	0	0	1.00	a	0	0	1.19	A	0	0	1.00	a	0	0	1.00
4423366	G	A	0	0	1.00	A	0	0	1.04	A	0	0	1.00	A	0	0	1.00	A	0	0	1.00	A	0	0	1.00
4460688	C	T	0	0	1.00	T	0	0	1.00	T	0	0	1.00	T	0	0	1.00	t	0	0	1.00	T	0	0	1.19
4465967	C	A	0	0	1.04	A	0	0	1.00	A	0	0	1.06	A	0	0	1.00	A	0	0	1.04	A	0	0	1.00
4628496	C	A	0	0	1.00	A	0	0	1.00	A	0	0	1.02	A	0	0	1.00	A	0	0	1.00	a	0	0	1.00
4629169	G	A	0	0	1.03	A	0	0	1.00	A	0	0	1.02	A	0	0	1.00	A	0	0	1.00	a	0	0	1.22

Position	LSD023.A				LVC_merged				PET004.A				UNT003.A				UNT004.A				VAL001.B				DA101 call	WAG001.A call	No. of genomes passing criteria
	SNP Call	Uncovered positions in 50 bp	Heterozygous SNPs in 50 bp	Mean coverage ratio LS/HS	SNP Call	Uncovered positions in 50 bp	Heterozygous SNPs in 50 bp	Mean coverage ratio LS/HS	SNP Call	Uncovered positions in 50 bp	Heterozygous SNPs in 50 bp	Mean coverage ratio LS/HS	SNP Call	Uncovered positions in 50 bp	Heterozygous SNPs in 50 bp	Mean coverage ratio LS/HS	SNP Call	Uncovered positions in 50 bp	Heterozygous SNPs in 50 bp	Mean coverage ratio LS/HS	SNP Call	Uncovered positions in 50 bp	Heterozygous SNPs in 50 bp	Mean coverage ratio LS/HS			
2419529	A	0	0	1.00	A	0	0	1.00	A	0	0	1.00	A	0	0	1.00	A	0	0	1.00	A	0	0	1.10	G	a	11
2495165	a	0	0	1.00	a	12	0	1.00	a	0	0	1.00	a	14	0	1.00	A	0	0	1.00	0	50	0	NA	C	0	6
2725715	t	0	0	1.00	T	0	0	1.00	T	0	0	1.00	T	0	0	1.00	T	0	0	1.00	T	0	0	1.00	T	0	10
2753572	T	0	0	1.00	T	0	0	1.00	T	0	0	1.07	t	0	0	1.00	T	0	0	1.00	T	0	0	1.00	A	0	10
2977542	A	0	0	1.10	A	0	0	1.07	A	0	0	1.00	A	0	0	1.00	A	0	0	1.00	A	0	0	1.08	A	0	9
3078807	A	0	0	1.00	A	0	0	1.00	A	0	0	1.00	A	0	0	1.00	A	0	0	1.00	A	0	0	1.00	C	0	10
3274298	T	0	0	1.00	T	0	0	1.00	T	0	0	1.00	T	0	0	1.00	T	0	0	1.00	T	0	0	1.00	t	0	11
3360963	C	0	0	1.08	C	0	0	1.12	C	0	0	1.00	0	16	0	1.56	0	43	0	1.00	C	0	0	1.07	A	0	2
3360984	T	0	0	1.18	T	0	0	1.12	T	0	0	1.00	t	16	0	1.63	t	22	0	1.00	T	0	0	1.17	C	0	2
3398153	A	0	0	1.00	a	0	0	1.00	A	0	0	1.00	A	0	0	1.00	a	1	0	1.00	A	0	0	1.00	G	0	9
3409414	0	31	0	1.00	0	12	0	1.05	0	26	0	1.00	C	0	0	1.00	c	7	0	1.00	0	26	0	1.00	T	0	5
3500922	G	0	0	1.00	G	0	0	1.00	G	0	0	1.00	G	0	0	1.00	g	0	0	1.00	G	0	0	1.00	T	0	10
3535148	T	0	0	1.00	T	0	0	1.00	T	0	0	1.00	T	0	0	1.00	T	0	0	1.00	T	0	0	1.00	G	0	11
3560088	A	0	0	1.16	A	0	0	1.00	A	0	0	1.00	A	0	0	1.00	A	0	1	1.00	A	0	0	1.00	G	0	9
3568597	T	0	0	1.00	T	0	0	1.00	T	0	0	1.00	T	0	0	1.05	T	0	0	1.00	T	0	0	1.00	C	0	9
3755861	t	12	0	1.00	t	22	0	1.00	t	1	0	1.00	t	15	0	1.00	t	7	1	1.00	0	37	0	1.00	C	0	1
3843195	A	0	0	1.00	A	0	0	1.00	A	0	0	1.00	A	0	0	1.00	A	0	0	1.00	A	0	0	1.00	C	0	10
3892488	T	0	0	1.00	T	0	0	1.00	T	0	0	1.19	T	0	0	1.00	T	0	0	1.00	T	0	0	1.00	C	0	10
4066494	t	0	0	1.00	T	2	0	1.24	0	16	0	1.00	T	0	0	1.00	t	0	0	1.00	T	0	0	1.00	C	0	7
4307755	A	0	0	1.00	A	0	0	1.00	A	0	0	1.00	A	0	0	1.00	A	0	0	1.00	A	0	0	1.00	G	0	11
4423366	A	0	0	1.00	A	0	0	1.11	A	0	0	1.07	A	0	0	1.00	A	0	0	1.00	A	0	0	1.00	G	0	9
4460688	T	0	0	1.00	T	0	0	1.00	T	0	0	1.00	T	0	0	1.00	T	0	0	1.00	T	0	0	1.00	T	0	11
4465967	A	0	0	1.03	A	0	0	1.00	A	0	0	1.00	A	0	0	1.04	A	0	0	1.00	A	0	0	1.00	c	0	7
4628496	A	0	0	1.00	A	0	0	1.00	A	0	0	1.00	A	0	0	1.00	a	0	0	1.00	A	0	0	1.00	C	0	11
4629169	A	0	0	1.00	A	0	0	1.16	A	0	0	1.00	A	0	0	1.00	A	0	0	1.12	A	0	0	1.00	G	0	7

Table S12: List of all modern and ancient *Y. pestis* genomes with accession number, sample origin and the corresponding publication.

**Modern strains**			
**Strain ID**	**Accession No.**	**Origin**	**Publication**
0.ANT1a_42013	ADPG00000000	China	Cui et al. 2013
0.ANT1b_CMCC49003	ADQX00000000	China	Cui et al. 2013
0.ANT1c_945	ADPV00000000	China	Cui et al. 2013
0.ANT1d_164	ADOW00000000	China	Cui et al. 2013
0.ANT1e_CMCC8211	ADRD00000000	China	Cui et al. 2013
0.ANT1f_42095	ADPJ00000000	China	Cui et al. 2013
0.ANT1g_CMCC42007	ADQV00000000	China	Cui et al. 2013
0.ANT1h_CMCC43032	ADQW00000000	China	Cui et al. 2013
0.ANT2_B42003004	AAYU00000000	China	Cui et al. 2013
0.ANT2a_2330	ADOY00000000	China	Cui et al. 2013
0.ANT3_231	JMUF00000000	FSU	Eroshenko et al. 2017
0.ANT3_790	CP006806	FSU	Zhgenti et al. 2015
0.ANT3_A-1486	LYMP00000000	FSU	Eroshenko et al. 2017
0.ANT3a_CMCC38001	ADQU00000000	China	Cui et al. 2013
0.ANT3b_A1956001	ADPX00000000	China	Cui et al. 2013
0.ANT3c_42082	ADPH00000000	China	Cui et al. 2013
0.ANT3d_CMCC21106	ADQP00000000	China	Cui et al. 2013
0.ANT3e_42091b	ADPI00000000	China	Cui et al. 2013
0.ANT5_262	QAGF00000000	FSU	Kutyrev et al. 2018
0.ANT5_5M	LYMQ00000000	FSU	Kutyrev et al. 2018
0.ANT5_A-1691	LYMQ00000000	FSU	Eroshenko et al. 2017
0.ANT5_A-1836	LYOL00000000	FSU	Eroshenko et al. 2017
0.PE2_1412	CP006783	FSU	Zhgenti et al. 2015
0.PE2_1413	CP006762	FSU	Zhgenti et al. 2015
0.PE2_14735	AYLS00000000	FSU	Zhgenti et al. 2015
0.PE2_1522	CP006758	FSU	Zhgenti et al. 2015
0.PE2_1670	CP006806	FSU	Zhgenti et al. 2015
0.PE2_3067	CP006754	FSU	Zhgenti et al. 2015
0.PE2_3544	LZNH00000000	FSU	Kutyrev et al. 2018
0.PE2_3551	MBSJ00000000	FSU	Kutyrev et al. 2018
0.PE2_3770	CP006751	FSU	Zhgenti et al. 2015
0.PE2_835_BPC	LYOJ00000000	FSU	Kutyrev et al. 2018
0.PE2_8787	CP006748	FSU	Zhgenti et al. 2015
0.PE2_C-197	LIYX00000000	FSU	Kislichkina et al. 2015
0.PE2_C-235	LIYY00000000	FSU	Kislichkina et al. 2015
0.PE2_C-267	LIYZ00000000	FSU	Kislichkina et al. 2015
0.PE2_C-290	LIYU00000000	FSU	Kislichkina et al. 2015
0.PE2_C-291	LIZC00000000	FSU	Kislichkina et al. 2015
0.PE2_C-346	LIZE00000000	FSU	Kislichkina et al. 2015
0.PE2_C-359	LIZB00000000	FSU	Kislichkina et al. 2015
0.PE2_C-370	MIDX00000000	FSU	Kislichkina et al. 2017
0.PE2_C-535	MIDY00000000	FSU	Kislichkina et al. 2017
0.PE2_C-537	LIYP00000000	FSU	Kislichkina et al. 2015
0.PE2_C-590	LIYQ00000000	FSU	Kislichkina et al. 2015
0.PE2_C-666	LIZF00000000	FSU	Kislichkina et al. 2015
0.PE2_C-678	MIDZ00000000	FSU	Kislichkina et al. 2017
0.PE2_C-700	MIEA00000000	FSU	Kislichkina et al. 2017
0.PE2_C-712	MTZW00000000	FSU	Kislichkina et al. 2017
0.PE2_C-739	MTZX00000000	FSU	Kislichkina et al. 2017
0.PE2_C-741	LPTX00000000	FSU	Kutyrev et al. 2018
0.PE2_C-746	MTZY00000000	FSU	Kislichkina et al. 2017
0.PE2_C-824	MTZZ00000000	FSU	Kislichkina et al. 2017

Modern strains			
**Strain ID**	**Accession No.**	**Origin**	**Publication**
0.PE2_KM874	LZTG00000000	FSU	Kutyrev et al. 2018
0.PE2_M-986	LYMO00000000	FSU	Kutyrev et al. 2018
0.PE2_PEST-F	NC_009381	FSU	Cui et al. 2013
0.PE2b_G8786	ADSG00000000	FSU	Cui et al. 2013
0.PE4_5307-Gis	LIYS00000000	FSU	Kislichkina et al. 2015
0.PE4_A-1804	LIYW00000000	FSU	Kislichkina et al. 2015
0.PE4_A-1807	LIYT00000000	FSU	Kislichkina et al. 2015
0.PE4_A-513	LIZA00000000	FSU	Kislichkina et al. 2015
0.PE4_I-3134	LIYR00000000	FSU	Kislichkina et al. 2015
0.PE4_I-3442	NHYH00000000	FSU	Kislichkina et al. 2018a
0.PE4_I-3443	MIED00000000	FSU	Kislichkina et al. 2018a
0.PE4_I-3446	NHYI00000000	FSU	Kislichkina et al. 2018a
0.PE4_I-3447	MIEE00000000	FSU	Kislichkina et al. 2018a
0.PE4_I-3455	LIYV00000000	FSU	Kislichkina et al. 2015
0.PE4_I-3515	NHYJ00000000	FSU	Kislichkina et al. 2018a
0.PE4_I-3516	NHMW00000000	FSU	Kislichkina et al. 2018a
0.PE4_I-3517	NHMX00000000	FSU	Kislichkina et al. 2018a
0.PE4_I-3518	NHMY00000000	FSU	Kislichkina et al. 2018a
0.PE4_I-3519	NHMZ00000000	FSU	Kislichkina et al. 2018a
0.PE4_M0000002	ADST00000000	China	Cui et al. 2013
0.PE4_Microtus91001	NC_005810	China	Cui et al. 2013
0.PE4a_B1313	LYMS00000000	FSU	Kutyrev et al. 2018
0.PE4a_I-2751-55	LYCL00000000	FSU	Kutyrev et al. 2018
0.PE4a_I-2998	LYMR00000000	FSU	Kutyrev et al. 2018
0.PE4Aa_12	ADOV00000000	China	Cui et al. 2013
0.PE4Ab_9	ADPT00000000	China	Cui et al. 2013
0.PE4Ba_PestoidesA	ACNT00000000	FSU	Cui et al. 2013
0.PE4Ca_CMCCN010025	ADRT00000000	China	Cui et al. 2013
0.PE4Cc_CMCC18019	ADQO00000000	China	Cui et al. 2013
0.PE4Cd_CMCC93014	ADRM00000000	China	Cui et al. 2013
0.PE4Ce_CMCC91090	ADRJ00000000	China	Cui et al. 2013
0.PE4h_A-1249	LYMN00000000	FSU	Eroshenko et al. 2017
0.PE4m_I-3086	LZNY00000000	Mongolia	Kutyrev et al. 2018
0.PE4t_A-1815	LPTY00000000	FSU	Eroshenko et al. 2017
0.PE5_I-2231	PVLX00000000	Mongolia	Kislichkina et al. 2018b
0.PE5_I-2236	PVLZ00000000	Mongolia	Kislichkina et al. 2018b
0.PE5_I-2238	PVLX00000000	Mongolia	Kislichkina et al. 2018b
0.PE5_I-2239	LIZD00000000	Mongolia	Kislichkina et al. 2015
0.PE5_I-2422a	LIZG00000000	Mongolia	Kislichkina et al. 2015
0.PE5_I-2422b	QANK00000000	Mongolia	Kutyrev et al. 2018
0.PE5_I-2457	PVMB00000000	Mongolia	Kislichkina et al. 2018b
0.PE5_I-3189	LIYO00000000	Mongolia	Kislichkina et al. 2015
0.PE5_I-3190	PVLY00000000	Mongolia	Kislichkina et al. 2018b
0.PE7b_620024	ADPM00000000	China	Cui et al. 2013
1.ANT1_Antiqua	NC_008150	Congo	Cui et al. 2013
1.ANT1_UG05-0454	AAYR00000000	Uganda	Cui et al. 2013
1.IN1a_CMCC11001	ADQK00000000	China	Cui et al. 2013
1.IN1b_780441	ADPS00000000	China	Cui et al. 2013
1.IN1c_K21985002	ADSS00000000	China	Cui et al. 2013
1.IN2a_CMCC640047	ADRA00000000	China	Cui et al. 2013
1.IN2b_30017	ADPC00000000	China	Cui et al. 2013
1.IN2c_CMCC31004	ADQR00000000	China	Cui et al. 2013
1.IN2d_C1975003	ADPZ00000000	China	Cui et al. 2013
1.IN2e_C1989001	ADQB00000000	China	Cui et al. 2013
1.IN2f_710317	ADPP00000000	China	Cui et al. 2013
1.IN2g_CMCC05013	ADQF00000000	China	Cui et al. 2013

Modern strains			
**Strain ID**	**Accession No.**	**Origin**	**Publication**
1.IN2h_5	ADPK00000000	China	Cui et al. 2013
1.IN2i_CMCC10012	ADQG00000000	China	Cui et al. 2013
1.IN2j_CMCC27002	ADQQ00000000	China	Cui et al. 2013
1.IN2k_970754	ADPW00000000	China	Cui et al. 2013
1.IN2l_D1991004	ADRX00000000	China	Cui et al. 2013
1.IN2m_D1964002b	ADRV00000000	China	Cui et al. 2013
1.IN2n_CMCC02041	ADQC00000000	China	Cui et al. 2013
1.IN2o_CMCC03001	ADQD00000000	China	Cui et al. 2013
1.IN2p_D1982001	ADRW00000000	China	Cui et al. 2013
1.IN2q_D1964001	ADRU00000000	China	Cui et al. 2013
1.IN3a_F1954001	ADSC00000000	China	Cui et al. 2013
1.IN3b_E1979001	AAYV00000000	China	Cui et al. 2013
1.IN3c_CMCC84038b	ADRF00000000	China	Cui et al. 2013
1.IN3d_YN1683	ADTD00000000	China	Cui et al. 2013
1.IN3e_YN472	ADTH00000000	China	Cui et al. 2013
1.IN3f_YN1065	ADTC00000000	China	Cui et al. 2013
1.IN3g_E1977001	ADRY00000000	China	Cui et al. 2013
1.IN3h_CMCC84033	ADRE00000000	China	Cui et al. 2013
1.IN3i_CMCC84046	ADRG00000000	China	Cui et al. 2013
1.ORI1_CA88	ABCD00000000	USA	Cui et al. 2013
1.ORI1_CO92	NC_003143	USA	Cui et al. 2013
1.ORI1a_CMCC114001	ADQL00000000	China	Cui et al. 2013
1.ORI1b_India195	ACNR00000000	India	Cui et al. 2013
1.ORI1c_F1946001	ADSB00000000	China	Cui et al. 2013
1.ORI2_F1991016	ABAT00000000	China	Cui et al. 2013
1.ORI2a_YN2179	ADTE00000000	Myanmar	Cui et al. 2013
1.ORI2c_YN2551b	ADTF00000000	China	Cui et al. 2013
1.ORI2d_YN2588	ADTG00000000	China	Cui et al. 2013
1.ORI2f_CMCC87001	ADRH00000000	China	Cui et al. 2013
1.ORI2g_F1984001	ADSD00000000	China	Cui et al. 2013
1.ORI2h_YN663	ADTI00000000	China	Cui et al. 2013
1.ORI2i_CMCCK100001a	ADRR00000000	China	Cui et al. 2013
1.ORI2i_CMCCK110001b	ADRS00000000	China	Cui et al. 2013
1.ORI3_IP275	AAOS00000000	Madagascar	Cui et al. 2013
1.ORI3_MG05-1020	AAYS00000000	Madagascar	Cui et al. 2013
1.ORI3a_EV76	ADSA00000000	Madagascar	Cui et al. 2013
2.ANT1_Nepal516	ACNQ00000000	Nepal	Cui et al. 2013
2.ANT1a_34008	ADPD00000000	China	Cui et al. 2013
2.ANT1b_34202	ADPE00000000	China	Cui et al. 2013
2.ANT2a_2	ADOX00000000	China	Cui et al. 2013
2.ANT2b_351001	ADPF00000000	China	Cui et al. 2013
2.ANT2c_CMCC347001	ADQS00000000	China	Cui et al. 2013
2.ANT2d_G1996006	ADSE00000000	China	Cui et al. 2013
2.ANT2e_G1996010	ADSF00000000	China	Cui et al. 2013
2.ANT2f_CMCC348002	ADQT00000000	China	Cui et al. 2013
2.ANT3_KM682	LPVG00000000	FSU	Kutyrev et al. 2018
2.ANT3a_CMCC92010	ADRL00000000	China	Cui et al. 2013
2.ANT3b_CMCC95001	ADRN00000000	China	Cui et al. 2013
2.ANT3c_CMCC96001	ADRO00000000	China	Cui et al. 2013
2.ANT3d_CMCC96007	ADRP00000000	China	Cui et al. 2013
2.ANT3e_CMCC67001	ADRB00000000	China	Cui et al. 2013
2.ANT3f_CMCC104003	ADQH00000000	China	Cui et al. 2013
2.ANT3g_CMCC51020	ADQY00000000	China	Cui et al. 2013
2.ANT3h_CMCC106002	ADQI00000000	China	Cui et al. 2013
2.ANT3i_CMCC64001	ADQZ00000000	China	Cui et al. 2013
2.ANT3j_H1959004	ADSI00000000	China	Cui et al. 2013

Modern strains			
**Strain ID**	**Accession No.**	**Origin**	**Publication**
2.ANT3k_5761	ADPL00000000	Russia	Cui et al. 2013
2.ANT3l_735	ADPR00000000	Russia	Cui et al. 2013
2.MED0_C-627	MBSI00000000	FSU	Kutyrev et al. 2018
2.MED1_1045	CP006794	FSU	Zhgenti et al. 2015
2.MED1_1116-D	LPXS00000000	FSU	Kutyrev et al. 2018
2.MED1_1240	LZNI00000000	FSU	Kutyrev et al. 2018
2.MED1_139	QAPA00000000	FSU	Kutyrev et al. 2018
2.MED1_173	LQAZ00000000	FSU	Kutyrev et al. 2018
2.MED1_1906	LYOM00000000	FSU	Kutyrev et al. 2018
2.MED1_244	LZND00000000	FSU	Kutyrev et al. 2018
2.MED1_261	LZNG00000000	FSU	Kutyrev et al. 2018
2.MED1_2944	CP006792	FSU	Zhgenti et al. 2015
2.MED1_44	LZNF00000000	FSU	Kutyrev et al. 2018
2.MED1_A-1763	LQAW00000000	FSU	Kutyrev et al. 2018
2.MED1_A-1809	LYMF00000000	FSU	Eroshenko et al. 2017
2.MED1_A-1825	LYCM00000000	FSU	Kutyrev et al. 2018
2.MED1_A-1920	LYCO00000000	FSU	Kutyrev et al. 2018
2.MED1_C-791	LQAU00000000	FSU	Kutyrev et al. 2018
2.MED1_K1973002	AAYT00000000	China	Cui et al. 2013
2.MED1_KIM10	NC_004088	Iran	Cui et al. 2013
2.MED1_KM816	LPXU00000000	FSU	Kutyrev et al. 2018
2.MED1_KM918	LPQY00000000	FSU	Kutyrev et al. 2018
2.MED1_M-1448	LYCN00000000	FSU	Kutyrev et al. 2018
2.MED1_M-1453	LQAY00000000	FSU	Kutyrev et al. 2018
2.MED1_M-1484	LQAV00000000	FSU	Kutyrev et al. 2018
2.MED1_M-1524	LYCP00000000	FSU	Kutyrev et al. 2018
2.MED1_M-1773	LYMG00000000	FSU	Kutyrev et al. 2018
2.MED1_M-1864	LOHR00000000	FSU	Kutyrev et al. 2018
2.MED1_M-519	LQAX00000000	FSU	Kutyrev et al. 2018
2.MED1_M-549	LQBA00000000	FSU	Kutyrev et al. 2018
2.MED1_M-595	LYOH00000000	FSU	Kutyrev et al. 2018
2.MED1_M-978	LPXT00000000	FSU	Kutyrev et al. 2018
2.MED1b_2506	ADPA00000000	China	Cui et al. 2013
2.MED1c_2654	ADPB00000000	China	Cui et al. 2013
2.MED1d_2504	ADOZ00000000	China	Cui et al. 2013
2.MED2b_91	ADPU00000000	China	Cui et al. 2013
2.MED2c_K11973002	AAYT00000000	China	Cui et al. 2013
2.MED2d_A1973001	ADPY00000000	China	Cui et al. 2013
2.MED2e_7338	ADPQ00000000	China	Cui et al. 2013
2.MED3a_J1963002	ADSP00000000	China	Cui et al. 2013
2.MED3b_CMCC125002b	ADQN00000000	China	Cui et al. 2013
2.MED3c_I1969003	ADSK00000000	China	Cui et al. 2013
2.MED3d_J1978002	ADSQ00000000	China	Cui et al. 2013
2.MED3f_I1970005	ADSL00000000	China	Cui et al. 2013
2.MED3g_CMCC99103	ADRQ00000000	China	Cui et al. 2013
2.MED3h_CMCC90027	ADRI00000000	China	Cui et al. 2013
2.MED3i_CMCC92004	ADRK00000000	China	Cui et al. 2013
2.MED3j_I2001001	ADSO00000000	China	Cui et al. 2013
2.MED3k_CMCC12003	ADQM00000000	China	Cui et al. 2013
2.MED3l_I1994006	ADSN00000000	China	Cui et al. 2013
2.MED3m_SHAN11	ADTA00000000	China	Cui et al. 2013
2.MED3n_SHAN12	ADTB00000000	China	Cui et al. 2013
2.MED3o_I1991001	ADSM00000000	China	Cui et al. 2013
2.MED3p_CMCC107004	ADQJ00000000	China	Cui et al. 2013
3.ANT1a_7b	ADPN00000000	China	Cui et al. 2013
3.ANT1b_CMCC71001	ADRC00000000	China	Cui et al. 2013

Modern strains			
**Strain ID**	**Accession No.**	**Origin**	**Publication**
3.ANT1c_C1976001	ADQA00000000	China	Cui et al. 2013
3.ANT1d_71021	ADPO00000000	China	Cui et al. 2013
3.ANT2a_MGJZ6	ADSX00000000	Mongolia	Cui et al. 2013
3.ANT2b_MGJZ7	ADSY00000000	Mongolia	Cui et al. 2013
3.ANT2c_MGJZ9	ADSZ00000000	Mongolia	Cui et al. 2013
3.ANT2d_MGJZ11	ADSU00000000	Mongolia	Cui et al. 2013
3.ANT2e_MGJZ3	ADSW00000000	Mongolia	Cui et al. 2013
4.ANT_1454	LZNC00000000	FSU	Kutyrev et al. 2018
4.ANT_338	LZNX00000000	FSU	Kutyrev et al. 2018
4.ANT_517	LYMH00000000	FSU	Kutyrev et al. 2018
4.ANT_KM932	LZNE00000000	FSU	Kutyrev et al. 2018
4.ANT_M-1944	LYOK00000000	FSU	Kutyrev et al. 2018
4.ANT1a_MGJZ12	ADSV00000000	Mongolia	Cui et al. 2013
**Ancient strains**			
**Strain ID**	**Accession No.**	**Origin**	**Publication**
DA101	PRJEB25891	Kyrgyzstan	Damgaard et al. 2018
Altenerding	PRJEB14851	Germany	Feldman et al. 2016
8124/8291/11972	SRR341961, SRR341962, SRR341963	United Kingdom	Bos et al. 2011
Ellwangen	PRJEB13664	Germany	Spyrou et al. 2016
Bolgar	PRJEB13664	Russia	Spyrou et al. 2016
OBS107	PRJEB12163	France	Bos et al. 2016
OBS110	PRJEB12163	France	Bos et al. 2016
OBS116	PRJEB12163	France	Bos et al. 2016
OBS124	PRJEB12163	France	Bos et al. 2016
OBS137	PRJEB12163	France	Bos et al. 2016

Table S13: Radiocarbon and archaeological dates of all individuals from plague-positive contexts in this study and of two published sites. Raw dates were calibrated with IntCal13 in OxCal v4.3.2. Archaeological dates are based on associated grave goods and cemetery stratigraphy. The probability plots for all dates are shown in SI Appendix, Fig. S8.

Site	Arch. ID	Lab ID	Radiocarbon dating lab ID	Skeletal element	Conventional Radiocarbon Age BP	$\delta^{13}C$ AMS [‰]	cal AD 2σ	Arch. Dating	Source
	Previously published individuals								
Altenerding	1175	AE1175	MAMS21886	Metacarpus	1541±19	-21.1	427–573	530–570	(3)
Altenerding	1176	AE1176	MAMS21887	Metacarpus	1563±20	-22.9	425–546	530–570	(3)
Aschheim	58	A58	MAMS15897	Rib	1563±18	-18.4	426–544	530–570	(19, 56)
Aschheim	76	A76	MAMS15898	Rib	1536±18	-21.6	428–580	530–570	(19)
Aschheim	120	A120	MAMS16593	Rib	1525±21	-17.2	430–599	525–550	(2)
	Individuals identified in this study								
Dittenheim	18A	DIT003	MAMS34551	Rib	1533±25	-31.2	428–595	500–600	This study
Edix Hill	Sk405	EDI001	MAMS38612	Rib	1497±24	-28.7	474–637	500–550	This study
Lunel-Viel	1	LVC001	MAMS34561	Femur	1492±23	-13.1	539–632	400–600	This study
Lunel-Viel	2	LVC002	MAMS34562	Femur	1474±24	-21.7	550–640	400–600	This study
Lunel-Viel	3A	LVC003	MAMS34563	Femur	1471±23	-19.6	555–641	400–600	This study
Lunel-Viel	4	LVC005	MAMS34554	Femur	1505+24	-24.9	434–618	400–600	This study
Lunel-Viel	5	LVC006	MAMS34565	Femur	1485 ±24	-17.6	543–635	400–600	This study
Lunel-Viel	6	LVC007	MAMS34566	Femur	1453±24	-25.1	567–648	400–600	This study
Petting	378	PET004	MAMS31028	Rib	1517±17	-22.3	434–600	570–600	This study
Petting	628	PET005	MAMS31033	Rib	1487±21	-23.8	542–630	530–730	This study
Petting	630	PET006	MAMS31034	Rib	1551±20	-19.6	427–558	570–600	This study
Saint-Doulchard	F206-40	LSD003	Ly-15568	Bone	1307±31	-20.6	656–770	650–800	(20)
Saint-Doulchard	F206-138	LSD027	Ly-15581	Bone	1235±30	-20.8	686–880	650–800	(20)
Saint-Doulchard	F206-145	LSD028	Ly-15625	Bone	1269±28	-20.2	665–853	650–800	(20)
Unterthürheim	129	UNT002	MAMS31035	Rib	1555±20	-19.0	427–553	550–600	This study
Unterthürheim	130	UNT003	MAMS31029	Rib	1520±17	-21.9	433–599	550–600	This study
Unterthürheim	131	UNT004	MAMS31036	Rib	1587±20	-20.9	419–537	550–600	This study
Unterthürheim	132	UNT005	MAMS34558	Rib	1505±24	-24.6	434–618	550–600	This study
Unterthürheim	134	UNT008	MAMS34568	Rib	1576±23	-23.2	421–542	550–600	This study
Unterthürheim	63	UNT006	MAMS31037	Rib	1507±20	-22.7	437–611	550–600	This study
Valencia	41/1657	VAL001	MAMS29429	Tooth	1512±22	-18.2	432–610	500–700	This study
Waging	199	WAG001	MAMS31030	Rib	1547±16	-15.7	427–563	500–550	This study

Table S14: Table giving all unique and shared SNPs that were called in the re-analyzed Aschheim genome (A120), showing the classification as assessed to the other genomes. The classification is based on the preceding parameters regarding a 50 bp window surrounding the SNP: Only SNPs with no uncovered position, no heterozygous SNP and an equal mean coverage with high and low stringent mapping (ratio of 1.00) would be classified as true positive SNPs (highlighted in green, and blue if the coverage is below 5).

Unique SNPs				
Position	SNP coverage	Uncovered pos.	Heterozygous SNPs	Mean coverage ratio LS/HS
22040	4	0	0	1.11
68401	4	6	1	1.11
90292	4	0	3	1.00
111916	3	24	0	1.27
118450	3	0	1	1.00
118467	5	0	1	1.06
119550	4	0	1	1.16
164957	4	5	1	1.24
206282	5	0	1	1.00
208068	4	0	2	1.00
208083	4	0	2	1.00
216157	8	0	1	3.93
221811	10	20	0	2.88
221849	11	0	2	2.76
222670	4	0	2	1.17
227200	4	0	2	1.09
228754	4	4	1	1.00
229308	4	8	0	2.08
229316	4	6	0	2.09
290016	4	0	2	1.00
318963	3	13	0	1.00
344159	4	0	0	1.00
352307	4	0	2	1.19
362357	4	17	0	5.11
363792	5	0	1	1.00
395774	37	0	0	4.65
439022	3	0	0	1.00
439036	3	0	0	1.00
473451	4	20	1	2.76
490767	4	7	1	1.24
500745	4	0	1	1.00
546706	3	0	0	1.03
567757	3	0	0	1.00
573139	4	0	1	0.99
573636	3	0	0	1.00
609900	5	13	1	2.54
610872	3	23	0	2.11
610908	6	19	1	2.13
705739	3	0	0	1.00
717675	4	24	2	1.44
723724	4	0	2	1.00
740842	3	6	0	1.15
788396	4	0	1	1.00
869369	4	0	2	1.26
912841	4	0	2	1.00
951452	4	0	1	1.00
1000454	4	2	1	1.00

Unique SNPs				
Position	SNP coverage	Uncovered pos.	Heterozygous SNPs	Mean coverage ratio LS/HS
1017104	4	0	0	1.00
1029706	5	1	1	1.00
1179456	4	0	1	1.00
1278057	3	22	1	1.30
1287721	5	0	1	1.00
1308293	37	0	1	1.75
1349463	4	0	2	1.00
1349472	4	0	2	1.00
1365939	4	0	1	1.00
1371025	10	2	0	3.22
1389874	3	0	1	1.09
1421032	27	0	2	1.75
1444672	4	0	1	1.15
1451459	4	0	2	1.27
1487354	4	0	1	1.30
1498866	3	0	0	1.00
1534269	4	0	1	1.00
1543236	4	10	2	1.00
1555873	4	0	2	1.00
1572689	3	12	0	12.78
1610886	3	9	0	1.00
1711880	3	0	0	1.27
1715867	5	0	0	1.20
1722937	6	0	1	1.21
1745586	4	0	1	1.13
1761696	4	0	1	1.00
1806990	4	7	2	1.06
1854844	4	14	1	1.28
1864422	4	0	2	1.00
1927370	3	8	0	1.00
1943523	6	7	1	1.57
1959365	4	0	1	1.00
2009537	4	1	1	1.00
2072340	4	0	1	1.33
2072914	5	0	1	2.38
2084670	4	0	1	1.10
2336322	3	12	0	1.00
2345720	5	0	1	1.00
2350460	4	0	1	1.26
2361259	4	5	2	1.46
2415931	5	0	1	1.00
2417705	4	0	1	1.13
2459674	4	0	1	1.00
2513785	4	0	2	1.00
2586708	4	0	1	1.00
2591088	4	0	3	2.86
2664231	4	0	2	1.00
2714188	5	0	1	1.37
2836744	4	6	1	1.00
2847001	4	0	1	1.36
2861901	3	21	0	1.00
2861902	3	20	0	1.00
2863957	4	0	2	1.00
2865494	3	3	0	3.19
2972201	4	0	1	1.00
3070014	44	0	3	1.68

Unique SNPs				
Position	SNP coverage	Uncovered pos.	Heterozygous SNPs	Mean coverage ratio LS/HS
3070065	56	0	1	1.38
3074956	3	23	0	1.16
3090759	4	0	1	1.00
3131243	3	15	0	1.00
3152585	4	0	3	1.39
3205018	4	0	3	1.15
3242622	3	2	0	1.21
3243987	4	0	1	1.41
3247821	4	0	0	1.00
3249954	4	1	2	1.00
3263455	4	0	1	1.24
3266505	3	10	1	1.31
3286005	3	0	1	1.00
3286017	4	0	1	1.00
3288015	4	0	2	1.00
3342782	4	6	1	1.00
3373238	5	0	2	1.00
3392897	18	2	1	3.16
3428541	4	0	1	1.00
3429285	6	0	1	3.25
3479274	4	0	2	1.00
3498406	4	0	1	1.03
3505837	4	0	1	1.00
3531163	4	0	1	1.40
3531197	4	0	1	1.14
3540557	4	0	3	1.87
3543444	3	0	1	1.00
3569690	4	0	1	1.25
3579698	4	0	1	1.32
3588681	3	8	0	1.00
3656870	4	0	1	1.11
3672205	8	0	1	1.35
3813424	7	23	0	2.51
3860098	4	0	1	1.07
3934112	3	7	0	1.03
3956001	9	0	2	2.96
3956018	4	0	5	3.46
4170791	45	0	1	1.80
4199220	30	0	0	6.01
4203620	23	0	1	6.18
4232217	3	8	0	1.00
4342798	4	12	1	1.78
4509055	26	0	0	2.46
4550310	3	0	1	1.26
4575345	33	0	0	4.52

Shared SNPs				
Position	SNP coverage	Uncovered pos.	Heterozygous SNPs	Mean coverage ratio LS/HS
86824	2	0	0	1.00
189912	0	23	0	1.00
260148	0	34	0	2.39
271114	3	0	0	1.48
485976	3	0	0	1.00
549767	2	0	0	1.00
557841	1	0	0	1.80
727741	5	0	2	1.53
779365	2	0	0	1.00
898980	5	0	1	1.16
1067966	6	0	0	1.11
1211729	1	0	0	1.00
1296743	1	0	0	1.58
1387701	2	0	0	1.00
1413031	0	24	0	4.23
1434752	2	6	1	1.00
1489055	2	1	0	1.42
1530658	1	16	0	1.38
1609461	2	0	2	1.29
1754708	2	0	1	1.50
1868678	3	0	1	1.00
1956162	5	0	1	1.24
2092152	2	0	0	1.85
2097520	1	0	0	1.00
2352174	1	0	0	1.21
2419529	0	24	0	1.00
2725715	3	0	0	1.00
2753572	1	0	0	1.00
2977542	0	15	0	1.00
3078807	1	18	0	1.48
3274298	3	0	1	1.00
3360963	0	41	0	4.04
3360984	0	50	0	NA
3398153	1	0	0	1.00
3409414	18	0	0	1.00
3500922	3	0	0	1.17
3535148	2	0	1	1.10
3560088	3	0	0	1.00
3568597	3	0	0	1.47
3750736	3	0	1	1.58
3843195	3	0	0	1.08
3892488	1	0	2	1.02
4066494	5	0	1	1.12
4307755	3	0	0	1.00
4412624	2	0	0	1.28
4423366	4	0	0	1.37
4460688	8	0	0	1.07
4465967	8	0	0	1.02
4628496	4	0	1	1.00
4629169	10	0	0	1.18

References

1. Renaud G, Hanghøj K, Willerslev E, Orlando L (2017) Sequence analysis gargammel: a sequence simulator for ancient DNA. *Bioin* 33:577–579.
2. Wagner DM, et al. (2014) Yersinia pestis and the Plague of Justinian 541–543 AD: A genomic analysis. *Lancet Infect Dis* 14(4):319–326.
3. Feldman M, et al. (2016) A High-Coverage Yersinia pestis Genome from a Sixth-Century Justinianic Plague Victim. *Mol Biol Evol* 33(11):2911–2923.
4. Deng Z, et al. (2015) TyrR, the regulator of aromatic amino acid metabolism, is required for mice infection of Yersinia pestis. *Front Microbiol* 6(110).
5. Chain PSG, et al. (2006) Complete genome sequence of Yersinia pestis strains antiqua and Nepal516: Evidence of gene reduction in an emerging pathogen. *J Bacteriol* 188(12):4453–4463.
6. Chain PSG, et al. (2004) Insights into the evolution of Yersinia pestis through whole-genome comparison with Yersinia pseudotuberculosis. *Proc Natl Acad Sci* 101(38):13826–13831.
7. Schmid A, et al. (2009) Cross-talk between type three secretion system and metabolism in Yersinia. *J Biol Chem* 284(18):12165–12177.
8. Coleman MA, et al. (2016) Expression and Association of the Yersinia pestis Translocon Proteins, YopB and YopD, Are Facilitated by Nanolipoprotein Particles. *PLoS One* 11(3):e0150166.
9. Deng W, et al. (2002) Genome Sequence of Yersinia pestis KIM. *J Bacteriol* 184(16):4601–4611.
10. Zhou D, et al. (2004) Genetics of Metabolic Variations between Yersinia pestis Biovars and the Proposal of a New Biovar, microtus. *J Bacteriol* 186(15):5147–5152.
11. Harper K (2017) *The Fate of Rome: Climate, Disease, and the End of an Empire* (Princeton University Press, Princeton).
12. Bos KI, et al. (2011) A draft genome of Yersinia pestis from victims of the Black Death. *Nature* 478(7370):506–10.
13. Spyrou MA, et al. (2016) Historical Y. pestis Genomes Reveal the European Black Death as the Source of Ancient and Modern Plague Pandemics. *Cell Host Microbe* 19(6):874–881.
14. McCormick M (2002) *Origins of the European Economy* (Cambridge University Press, Cambridge).
15. McCormick M (1998) Bateaux de vie, bateaux de mort. Maladie, commerce, transports annonaires et le passage économique du Bas-Empire au Moyen Âge. *Morfologie Sociali e Culturali in Europa* (Centro Italiano di Studi sull'Alto Medioevo, Spoleto), pp 35–118.
16. Benedictow OJ (2004) *The Black Death 1346–1353: The Complete History* (Boydell & Brewer, Woodsbridge).
17. Yue RPH, Lee HF, Wu CYH (2016) Navigable rivers facilitated the spread and recurrence of plague in pre-industrial Europe. *Sci Rep* 6(1):34867.
18. Roosen J, Curtis DR (2018) Dangers of noncritical use of historical plague data. *Emerg Infect Dis* 24(1):103–110.
19. Harbeck M, et al. (2013) Yersinia pestis DNA from Skeletal Remains from the 6th Century AD Reveals Insights into Justinianic Plague. *PLoS Pathog* 9(5):e1003349.
20. Maçon P, Durand R, Salin M, Dominin F (2011) *Le Pressoir - Rapport final d'opération de fouille préventive* (Bourges).
21. Reimer PJ, et al. (2013) IntCal13 and Marine13 Radiocarbon Age Calibration Curves 0–50,000 Years cal BP. *Radiocarbon* 55(4):1869–1887.
22. Ramsey CB (2017) Methods for Summarizing Radiocarbon Datasets. *Radiocarbon* 59(6):1809–1833.
23. Ascough PL, et al. (2010) Temporal and spatial variations in freshwater 14C reservoir effects: Lake Mývatn, northern Iceland. *Radiocarbon* 52(2–3):1098–1112.
24. Ascough P, Cook G, Dugmore A (2005) Methodological approaches to determining the marine radiocarbon reservoir effect. *Prog Phys Geogr* 4:532–547.
25. Barta P, Štolc S (2007) HBCO Correction: Its Impact on Archaeological Absolute Dating. *Radiocarbon* 49(2 "Proceedings of the 19th international Radiocarbon Conference, Keble

College, Oxford, England"):465–472.
26. Wamser L (2010) *Karfunkelstein und Seide. Neue Schätze aus Bayerns Frühzeit* (Pustet, Regensburg).
27. Grupe G, Harbeck M, McGlynn GC (2015) *Prähistorische Anthropologie* (Springer, Berlin Heidelberg).
28. Pöllath R (2002) *Karolingerzeitliche Gräberfelder in Nordostbayern: eine archäologisch-historische Interpretation mit der Vorlage der Ausgrabungen von K. Schwarz in Weismain und Thurnau-Alladorf* (München).
29. Leinthaler B (1988) Der Karolingisch-ottonische Ortsfriedhof Alladorf, Ldkr. Kulmbach. Die Grabungskampagne 1984. *Zeitschrift für Archäologie des Mittelalters* 16/17:7–122.
30. Christlein R (1971) *Das alamannische Gräberfeld von Dirlewang bei Mindelheim* (Kallmünz).
31. Haas-Gebhard B (1998) *Ein frühmittelalterliches Gräberfeld bei Dittenheim (D)* (Montagnac).
32. Malim T, Hines J (1998) *The Anglo-Saxon cemetery at Edix Hill (Barrington A), Cambridgeshire* (Council for British Archaeology, York).
33. Hines JA, Bayliss AL (2013) *Anglo-Saxon graves and grave goods of the Sixth and Seventh Centuries AD: A chronological framework.* (Society for Medieval Archaeology, Leeds). SMA Monogr.
34. Kropf E, Zintl S (2007) Eine frühmittelalterliche Vierfachbestattung aus Forchheim: Markt Pförring, Landkreis Eichstätt, Oberbayern. *Das archäologische Jahr Bayern* 2006:120–123.
35. von Freeden U (1983) Das frühmittelalterliche Gräberfeld von Grafendobrach in Oberfranken. *Bericht der Römisch-Germanischen Kommission* 64:417–507.
36. Pescheck C (1996) *Das fränkische Reihengräberfeld von Kleinlangheim, Lkr. Kitzingen/Nordbayern* (Mainz).
37. Daim F (1987) *Das awarische Gräberfeld von Leobersdorf, Niederösterreich* (Wien).
38. McCormick M (2016) Tracking mass death during the fall of Rome's empire (II): a first inventory of mass graves. *J Rom Archaeol* 29:1004–1046.
39. Raynaud C (2010) *Les nécropoles de Lunel-Viel (Hérault) de l'Antiquité au Moyen Âge. Revue archéologique de Narbonnaise Supplément 40* (Presses universitaires de la Méditerranée, Montpellier).
40. Barruol G, Garnotel A, Raynaud C (2017) Maguelone de l'Antiquité à nos jours: histoire, archéologie et environnement. *Palavas-Les-Flots: Les Compagnons de Maguelone*, pp 50–51.
41. Dannheimer H (1998) *Das baiuwarische Reihengräberfeld von Aubing, Stadt München* (Stuttgart).
42. Keller E (1979) *Das spätrömische Gräberfeld von Neuburg an der Donau* (Kallmünz). Materialhefte zur bayerischen Vorgeschichte
43. Bierbrauer V (2002) Neuburg. *Reallexikon Der Germanischen Altertumskunde, Vol. 21* (Berlin), pp 106–108.
44. von Freeden U, Lehmann D (2005) *Das frühmittelalterliche Gräberfeld von Peigen, Gem. Pilsting* (Landau a. d. Isar).
45. Haas-Gebhard B, Weindauer F (2013) Die Fibelgräber der frühmittelalterlichen Nekropole von Petting (Oberbayern). *Bayerische Vorgeschichtsblätter* 78:205–234.
46. Reimann D (1991) Byzantinisches aus dem Rupertiwinkel – Zum Ohrringpaar von Petting. *Das archäologische Jahr Bayern* (1991):143–145.
47. Loré F (2012) Gräber und kein Ende? Neue Grabungen im Großen Gräberfeld von Regensburg: Oberpfalz. *Das archäologische Jahr Bayern* 2011:96–98.
48. Menke H, Menke M (2013) *Das frühmittelalterliche Gräberfeld von Sindolvesdorf/Sindelsdorf, Lkr. Weilheim-Schongau* (Kallmünz).
49. Moosbauer G (2005) *Kastell und Friedhöfe der Spätantike in Straubing: Römer und Germanen auf dem Weg zu den ersten Bajuwaren* (Rahden). Passauer Universitätsschriften zur Archäologie.
50. Grünewald C (1988) *Das alamannische Gräberfeld von Unterthürheim, Bayerisch-Schwaben* (Kallmünz).
51. Alapont Martín L, Ribera i Lacomba AV (2009) Topografía y jerarquía funeraria en la Valencia tardo-antigua. *Morir en el Mediterráneo Medieval: Actas del III Congreso Internacional de Arqueología, Arte e Historia de La Antigüedad Tardía y Alta Edad Media*

Peninsular (Oxford), pp 59–88.
52. Ribera i Lacomba AV, Alapont Martín L (2006) Cementerios tardoantiguos de Valencia: arqueología y antropología. *An Arqueol Cordobesa* 17(2):161–194.
53. Ribera i Lacomba AV, Soriano R (1996) *Los cementerios de época visigoda* (València).
54. Knöchlein R (1998) *Das Reihengräberfeld von Waging am See* (Liliom, Waging am See).
55. Reiß R (1994) *Der merowingerzeitliche Reihengräberfriedhof von Westheim (Kreis Weißenburg-Gunzenhausen): Forschungen zur frühmittelalterlichen Landesgeschichte im südwestlichen Mittelfranken* (Nürnberg).
56. Haas-Gebhard B (2017) Die Pest des Frühen Mittelalters in der Münchner Schotterebene. *Bayerische Vorgeschichtsblätter* 82:191–210.
